# Isotopic constraints in methane inversions reveal larger trends in wetland emissions with improved linkage to terrestrial water storage

**DOI:** 10.1038/s41467-026-74777-4

**Published:** 2026-06-29

**Authors:** Youmi Oh, Lori Bruhwiler, Xin Lan, Santanu Halder, Ben Riddell-Young, Sourish Basu, John B. Miller, Sylvia Michel, Ken Schuldt, Arlyn Andrews, Sebastien Biraud, Łukasz Chmura, Tatiana Di lorio, Elise-Andree Guerette, Elena Kozlova, Licheng Liu, Simonetta Montaguti, Shinji Morimoto, Martin Steinbacher, Taku Umezawa, Irène Xueref-Remy, Giulia Zazzeri

**Affiliations:** 1 Cooperative Institute for Research in Environmental Science, University of Colorado, Boulder, CO USA; 2 Global Monitoring Laboratory, National Oceanic and Atmospheric Administration, Boulder, CO USA; 3 Earth System Science Interdisciplinary Center, University of Maryland, College Park, MD USA; 4 Institute for Arctic and Alpine Research, University of Colorado, Boulder, CO USA; 5 Department of Atmospheric and Oceanic Sciences, University of Colorado, Boulder, CO USA; 6 Climate & Ecosystem Sciences Division, Lawrence Berkeley National Laboratory, Berkeley, CA USA; 7 AGH University of Krakow, Faculty of Physics and Applied Computer Science, Krakow, Poland; 8 Institute of Meteorology and Water Management — National Research Institute, Warsaw, Poland; 9 Italian National Agency for New Technologies, Energy and Sustainable Economic Development (ENEA), Rome, Italy; 10 Environment, Commonwealth Scientific and Industrial Research Organisation (CSIRO), Aspendale, VIC Australia; 11 Department of Geography, University of Exeter, Exeter, UK; 12 Department of Bioproducts and Biosystems Engineering, University of Minnesota, St. Paul, MN USA; 13 National Research Council of Italy, Institute of Atmospheric Sciences and Climate (ISAC), Bologna, Italy; 14 Center for Atmospheric and Oceanic Studies, Graduate School of Science, Tohoku University, Sendai, Japan; 15 Empa, Swiss Federal Laboratories for Materials Science and Technology, Duebendorf, Switzerland; 16 National Institute for Environmental Studies, Tsukuba, Japan; 17 Institut Méditerranéen de Biodiversité et Ecologie Marine et Continentale (IMBE), Aix-Marseille Université, CNRS, Institut de Recherche pour le Développement (IRD), Avignon Université, Aix-en-Provence, France; 18 Research on the Energy System - RSE S.p.A., Milano, Italy

**Keywords:** Carbon cycle, Carbon cycle, Climate and Earth system modelling

## Abstract

Accurately separating the contributions of different sources to recent atmospheric methane (CH_4_) growth is crucial for better quantifying the present and future responses of CH_4_ emissions to changing climate and anthropogenic activity. Here, we run atmospheric inversions to assess global and regional CH_4_ emissions from microbial, fossil, and pyrogenic sources from 2000 to 2022, using measurements of atmospheric CH_4_ and its stable carbon isotope ratio (^13^C:^12^C, expressed relative to a standard as *δ*^13^C-CH_4_). We confirm that global total CH_4_ emissions has increased by 15% from 2000 to 2022, with dominated contribution from microbial emissions. Both microbial and fossil emissions increased during 2007–2013 relative to 2000–2006, with the largest contributions from temperate Asia. From 2014–2017, microbial emissions from tropical regions, particularly South America and Africa, were the dominant driver of the overall increase, while fossil emissions remained stable in Europe and North America. Between 2020 and 2022, microbial emissions surged in the African and Asian tropics, whereas fossil emissions declined across most industrial regions. Notably, inversions constrained only by CH_4_ observations did not capture the decline in fossil emissions during 2020–2022, a decline potentially related to the COVID-19 pandemic, policy-driven changes, and decreases in CH_4_ emissions intensity, highlighting the critical role of isotopic measurements in independently verifying changes in fossil emissions. Further, our inversion with isotopic constraints estimates a more prominent increase in wetland emissions that is 20–30% more strongly correlated with variations in terrestrial water storage during 2003–2022, demonstrating the importance of climate-driven natural sources in explaining long-term CH_4_ growth.

## Abstract (web summary)

This study shows that global methane growth from 2000–2022 was mainly driven by microbial sources, with isotopic data improving separation of microbial and fossil sources and revealing stronger links between wetland emissions and climate variability.

## Introduction

**Fig. 1:**
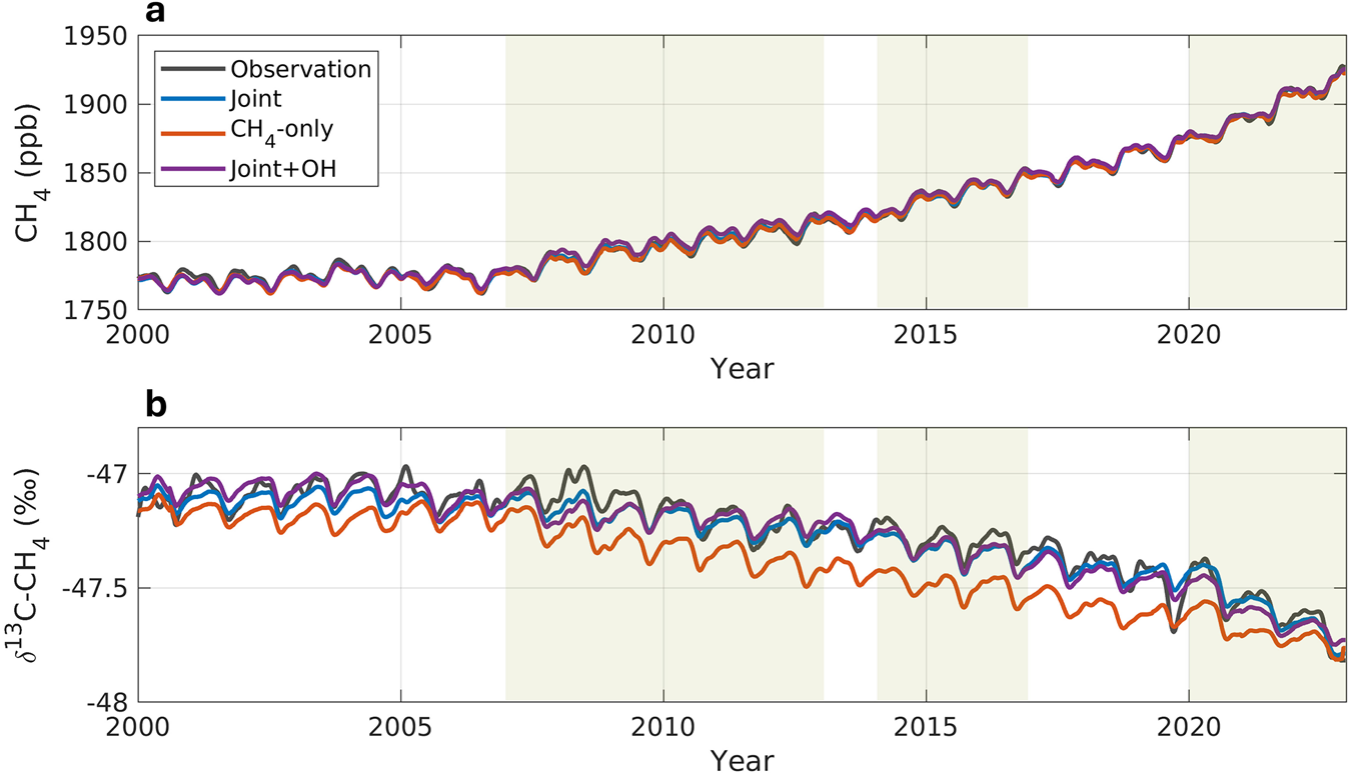
Observed and simulated long-term trends in atmospheric CH_4_ and *δ*^*1*3^C-CH_4_ during 2000–2022. Model-data comparison of long-term trends of **a** atmospheric CH_4_ (in ppb) and **b** *δ*^*1*3^C-CH_4_ (in ‰) by observation (black) and simulations from Joint (blue), CH_4_-only (red), and Joint+OH (purple) scenarios. The three green shaded regions denote the periods of rapid atmospheric CH_4_ increase examined in Figs. 2 and 3. Source data are provided as a Source Data file.

Measurements of global atmospheric methane mole fraction (CH_4_) exhibit a stable period of low growth from 1999 to 2006 followed by rapid growth: ~6 parts per billion (nanomoles of CH_4_ per mole dry air, or ppb) yr^−1^ between 2007 and 2013 and ~10 ppb yr^−1^ between 2014 and 2020^1^ (black line in Fig. 1a). In 2020–2022, atmospheric CH_4_ grew at the fastest rate (~15 ppb yr^−1^) recorded since NOAA’s systematic measurements started in 1983^1^. Observed global mean atmospheric CH_4_ reached 1930 ppb in 2024^1^, more than 250% higher than the pre-industrial level of 729 ppb^2,3^. Atmospheric CH_4_ levels are determined by the complex budget of sources and sinks^4^. Sources can be broadly categorized into anthropogenic microbial emissions, such as ruminants, rice cultivation, and wastewater/landfills; natural microbial sources, primarily wetlands; fossil/thermogenic emissions from oil, gas, coal, and geologic seeps; and pyrogenic sources. The primary sink is oxidation by tropospheric hydroxyl radicals (OH), with additional losses from reactions with Cl and O(^1^D) and from soil microbial uptake.

Many studies have explored possible drivers of the three rapid growth periods in atmospheric CH_4_ since the 2000s: 2007–2013, 2014–2017, and 2020–2022 (Supplementary Table S1). Changes in fossil fuel^4,5^, microbial^2,6–10^, and pyrogenic emissions as well as OH^11,12^ have been identified as potential contributors to the increase in atmospheric CH_4_ levels during 2007–2013. Studies incorporating satellite retrievals^13,14^ suggested that CH_4_ increases after 2014 could be linked to enhanced emissions from tropical wetlands^15–19^, increased anthropogenic emissions^5^, or changes in biomass burning and OH^20^. More recent studies have found that during the 2020–2022 COVID−19 pandemic, the unprecedented atmospheric growth was driven by a combination of increased tropical wetland emissions and decreased atmospheric OH sinks associated with reductions in chemical precursor emissions^21–24^, but the relative contributions of wetlands and reduced sinks vary among these studies. Consequently, the roles of CH_4_ sources and sinks in atmospheric growth from 2000 to 2022 remain uncertain, with studies reporting conflicting findings on the main drivers across different periods (Supplementary Table S1).

Measurements of atmospheric CH_4_ and its stable carbon isotope ratio (^13^C:^12^C, expressed relative to a standard as *δ*^13^C-CH_4_, Eq. (1)), in combination with measurements of isotopic signatures of sources and sinks, can be used to partition CH_4_ emissions among microbial, fossil, and pyrogenic sources^25,26^ (Supplementary Figs. S1-2). CH_4_ from microbial sources is more depleted in the heavy isotope (^13^C) (more negative *δ*^*1*3^C-CH_4_) than fossil and pyrogenic sources, which allows us to distinguish changes in atmospheric CH_4_ resulting from microbial or other emission categories. Atmospheric chemical and soil microbial CH_4_ sinks both favor oxidation of ^12^C, making the residual atmospheric CH_4_ relatively enriched in ^13^C (less negative *δ*^*1*3^C-CH_4_)^27,28^. Globally distributed observations show that as CH_4_ has increased since 2007, *δ*^*1*3^C-CH_4_ has declined (black line in Fig. 1b)^8^ after increasing for the last 200 years^2,28^. The atmospheric *δ*^*1*3^C-CH_4_ decrease was twice as fast in 2020–2022 than in 2008–2019. However, none of the 3-D atmospheric inversion studies that cover 2020–2022^18,21,24,29^ have yet used *δ*^*1*3^C-CH_4_ measurements as a constraint in their global and regional emissions estimation. This lack of isotopic constraints may lead to biased source partitioning and a higher sensitivity to prior assumptions when attributing changes among different sources.

Some studies have questioned the usefulness of atmospheric *δ*^13^C-CH_4_ measurements for constraining CH_4_ sources and sinks, citing large uncertainties in isotopic source signatures^30^. However, a more comprehensive uncertainty assessment based on an extensive isotopic signature inventory suggests otherwise^27^. Monte Carlo simulations using grid-level source signatures and emissions yield relatively small global mean uncertainties (<3‰) across different source types (Supplementary Table S2 and Supplementary Fig. S3). These uncertainties are substantially smaller than the typical isotopic differences between microbial, fossil, and pyrogenic sources (>15‰), indicating that *δ*^13^C-CH_4_ measurements can effectively differentiate between these sources. However, *δ*^13^C-CH_4_ on its own is not able to distinguish between microbial anthropogenic and natural emissions due to sparse atmospheric observations. While some recent studies attribute changes in atmospheric *δ*¹³C-CH_4_ to negative trends in the isotopic signature of fossil emissions^5,31^, our isotopic inventory shows that the fossil *δ*^13^C-CH_4_ shows positive trends^27^, consistent with the increased proportion of CH_4_ extracted from shale gas reserves^32^. Lastly, Basu et al.^7^ explored systematic uncertainties in source signatures by running inversions with alternative assumptions. They showed that these uncertainties have a relatively minor impact on source partitioning compared to other factors, such as changes in atmospheric OH.

The objective of this study is to estimate CH_4_ emissions from microbial, fossil, and pyrogenic sources using atmospheric inversion modeling and measurements of in-situ CH_4_ and *δ*^13^C-CH_4_ during 2000–2022 (Supplementary Fig. S4, Methods). The period starting in 2000 was selected due to the availability of *δ*^13^C-CH_4_ measurements. We use a four-dimensional variational (4DVar) flux optimization technique that adjusts prior (first guess) emissions at the monthly and grid-level scales to optimally fit globally distributed CH_4_ and *δ*^13^C-CH_4_ observations^7^. We used a comprehensive dataset of atmospheric CH_4_ measurements from ~350 sites and *δ*¹³C-CH_4_ measurements from ~70 sites for data assimilation and validation through collaboration with 30 laboratories worldwide (Methods, Supplementary Fig. S5)^33^. All data were rigorously quality-checked and intercompared across laboratories, then converted to common calibration scales^34^.

There are three inversion setups we compare in this paper (Methods, Fig. 1). First, the “CH_4_-only” scenario represents a conventional inversion approach that does not use information from *δ*^13^C-CH_4_ data (Supplementary Fig. S6a). Second, the “Joint (CH_4_ and *δ*^13^C-CH_4_)” scenario uses both CH_4_ and *δ*^13^C-CH_4_ to constrain CH_4_ budgets (Supplementary Fig. S6b). Lastly, the “Joint + OH” scenario is built upon the “Joint” scenario and adds long-term variability of tropospheric OH by setting a globally-applied tropospheric OH scaling factor to increase from 1 to 1.05 for 2000–2017 and then decrease to 1.02 for 2018–2022 (Supplementary Fig. S7a), based on results from the chemical transport model, LMDz-INCA^5,21,35^. More details on our prior sensitivity test, ensemble simulation, and correlation analyses can be found in Methods and Supplementary Information. Taken together, these experiments allow us to utilize *δ*^13^C-CH_4_ data and understand the impact of isotopic constraints on global and regional CH_4_ budgets.

## Results

### Model-data comparison of inversion scenarios

Posterior fluxes for all three scenarios largely eliminate the observed mismatch associated with prior fluxes for CH_4_ (Fig. 1, and Supplementary Figs. S8–11). The simulation using the prior emissions exhibited a pronounced hemispheric bias, with positive mismatches in the Northern Hemisphere and negative mismatches in the Southern Hemisphere (dark green in Supplementary Fig. S10), reflecting an inaccurate latitudinal distribution of prior fluxes. After data assimilation, this large mean bias and standard deviation (26 ± 42 ppb) were significantly reduced (−2 ± 32 ppb) (Supplementary Fig. S8a). Independent validation data not used in the inversion likewise showed a marked improvement, with mismatches decreasing from 24 ± 66 ppb for the prior to −4 ± 57 ppb (Supplementary Fig. S9a).

**Fig. 2:**
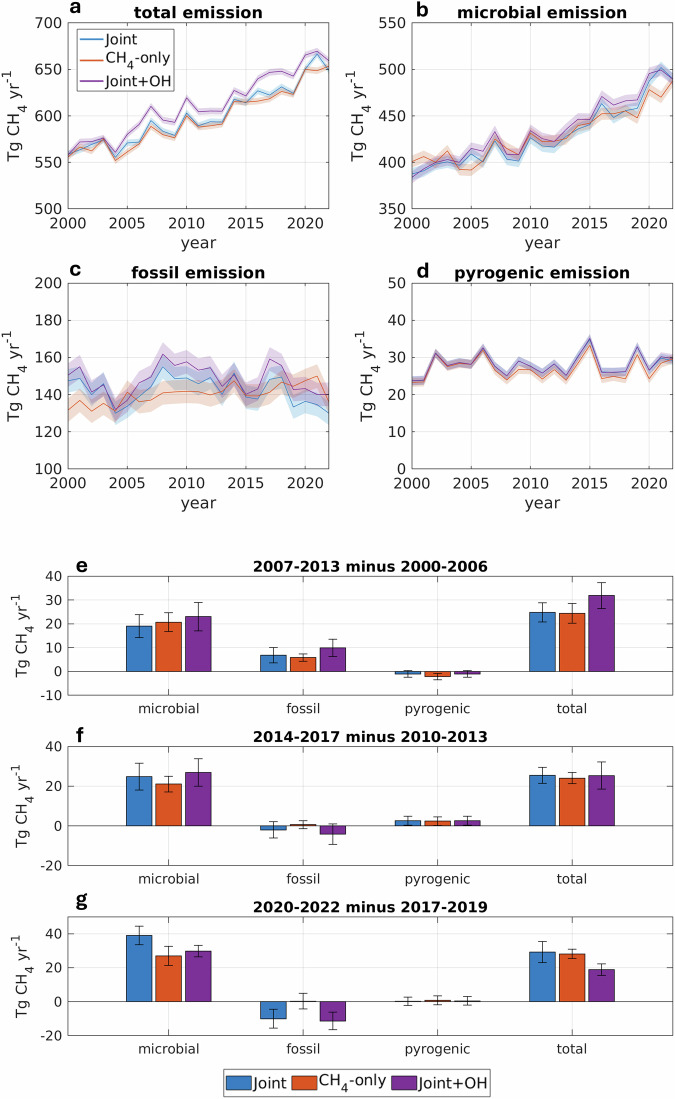
Trends and differences in global CH_4_ emissions during 2000–2022 for different scenarios. Global annual changes in CH_4_ emissions (in TgCH_4_ yr^−1^) from **a** total, **b** microbial, **c** fossil, and **d** pyrogenic sources for Joint (blue), CH_4_-only (red), and Joint+OH (purple) scenarios during 2000–2022. Differences in global CH_4_ emissions between **e** 2000–2006 and 2007–2013, **f** 2010–2013 and 2014–2017, and **g** 2017–2019 and 2020–2022 in TgCH_4_ yr^−1^ for Joint (blue), CH_4_-only (red), and Joint+OH (purple) scenarios. Uncertainty is estimated as one standard deviation of the ensemble simulation results (Supplementary Information). Source data are provided as a Source Data file.

For atmospheric *δ*^*1*3^C-CH_4_, prior fluxes produced negative (isotopically lighter) mismatches in the Northern Hemisphere and tropics (dark green in Supplementary Fig. S11) (mean and standard deviation −0.17 ± 0.19‰). Posterior fluxes from CH_4_-only exhibit a bias relative to *δ*^*1*3^C-CH_4_ measurements (red line in Fig. 1; −0.14 ± 0.20‰). The negative bias of the CH_4_-only posterior from observations is explained by its smaller mean fossil fluxes compared to the Joint scenario (Fig. 2c), as reported in previous studies^7,9^. However, posterior mismatches with observed *δ*^*1*3^C-CH_4_ were substantially reduced for the Joint and Joint+OH scenarios (0.01 ± 0.14 ‰, Supplementary Fig. S8b). Comparison with *δ*^*1*3^C-CH_4_ validation data similarly showed improved agreement, with mismatches declining from −0.17 ± 0.34‰ for the prior to −0.068 ± 0.33 and −0.064 ± 0.33‰ for the Joint and Joint+OH scenarios, respectively (Supplementary Fig. S9b).

### Source attribution of CH_4_ surges during 2000–2022

We estimate that CH_4_ emissions reached ~650 TgCH_4_ yr^−1^ in 2022, an increase of more than 15% since 2000 (Fig. 2). The total emission is larger than some other studies^18,29^ because our atmospheric lifetime of ~8.5 years is shorter than those studies (Supplementary Table S4). From 2000 to 2022, we observed an accelerating trend from 3.5 TgCH_4_ yr^−2^ over the 2000s to 6.0 TgCH_4_ yr^−2^ over the 2010s (Fig. 2).

**Fig. 3:**
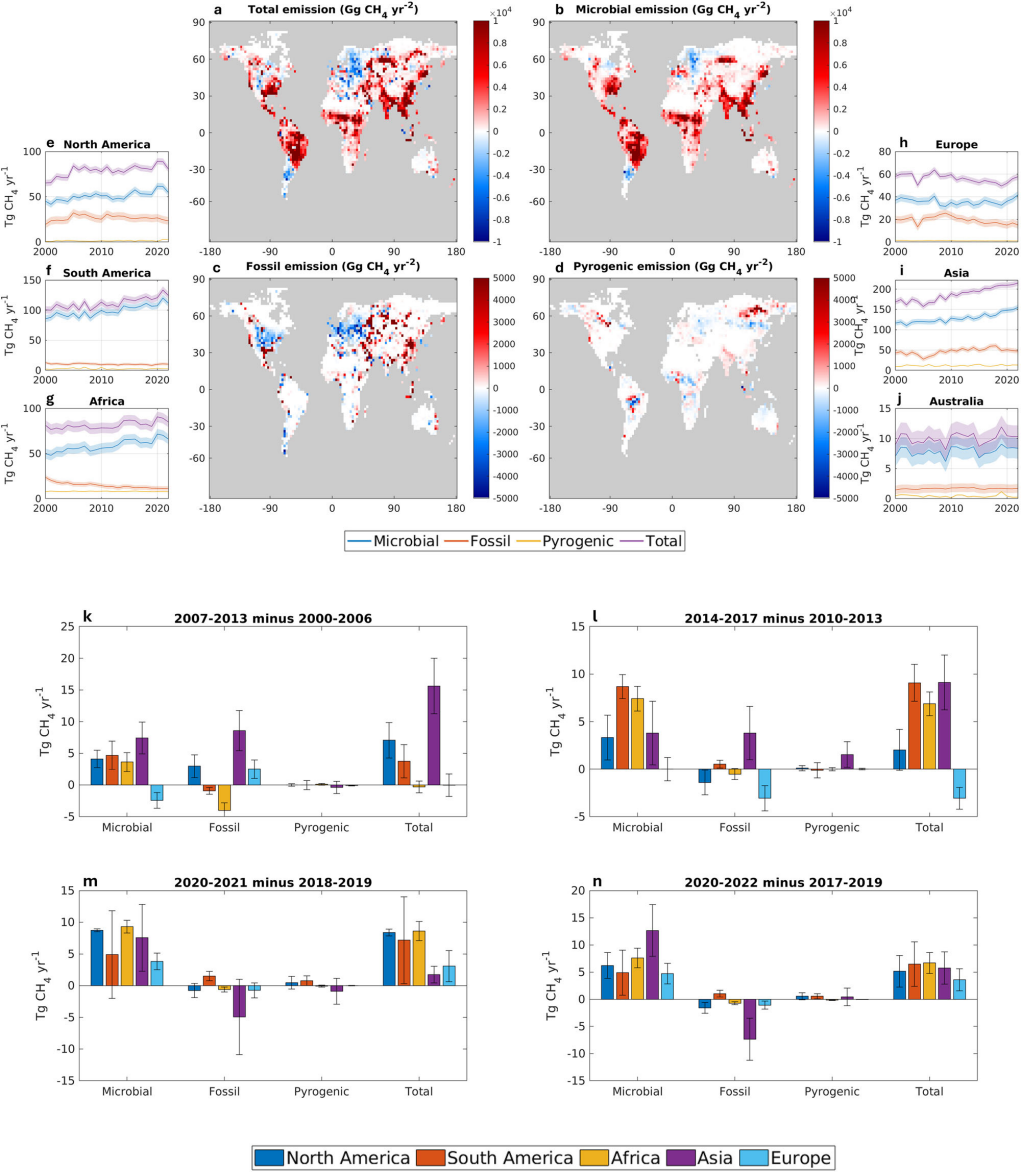
Trends and differences in continental CH_4_ emissions during 2000–2022 for the Joint Scenario. Spatial distribution of trends in long-term CH_4_ emissions in GgCH_4_ yr^−2^ from **a** total, **b** microbial, **c** fossil, and **d** pyrogenic sources during 2000–2022. **e**–**k** Long-term trends in regional CH_4_ emissions in TgCH_4_ yr^−1^ from **e** North America, **f** South America, **g** Africa, **h** Europe, **i** Asia, and **j** Australia from microbial (blue), fossil (red), pyrogenic (yellow), and total (purple) sources. Differences in regional CH_4_ emissions between **k** 2000–2006 and 2007–2013, **l** 2010–2013 and 2014–2017, **m** 2018–2019 and 2020–2021, and **n** 2017–2019 and 2020–2022 in TgCH_4_ yr^−1^ from North America (blue), South America (red), Africa (yellow), Asia (purple), and Europe (skyblue) continents. Uncertainty is estimated as one standard deviation of the ensemble simulation results (Supplementary Information). Source data are provided as a Source Data file.

We analyzed drivers of the three rapid growth periods since the 2000s: 2007–2013, 2014–2017, and 2020–2022 (Fig. 3). Based on the relative agreement with observations, in the following analyses, we refer only to the isotopically constrained emissions. During 2007–2013, the increase in global CH_4_ emissions (25 ± 4 TgCH_4_ yr^−1^) was driven by both microbial (19 ± 5 TgCH_4_ yr^−1^) and fossil (7 ± 3 TgCH_4_ yr^−1^) sources (Fig. 2e). Microbial emissions increased in most regions, but fossil fuel emissions increased mainly in Temperate Eurasia (8 ± 3 TgCH_4_ yr^−1^) (Fig. 3k), driven partly by increased coal production and use in China^36^. Coal production in China tripled between 2000 and 2013 and supplied 70% of China’s energy needs according to the international energy agency^37,38^. Despite significant investments in non-carbon energy sources, China’s total coal supply has increased by 268% since 2000^37,38^. Oil and gas production also rose during this period in parts of Central Asia, and although these emissions are estimated to be smaller than those from Chinese coal production, they could also contribute to our estimated trend^37^. These findings are consistent with studies highlighting increases in both microbial and fossil emissions^7,9^ but contrast with studies that attribute the CH_4_ rise to abrupt changes in biomass burning and atmospheric OH^11,12^ (Supplementary Table S1).

During 2014–2017, increases in both microbial (25 ± 7 TgCH_4_ yr^−1^) and biomass burning (3 ± 2 TgCH_4_ yr^−1^) emissions drove the atmospheric CH_4_ surge, while fossil emissions remained steady (−2 ± 4 TgCH_4_ yr^−1^) globally compared to 2010–2013 (Fig. 2f). The increases in microbial emissions were mainly from the tropics with only a minor increase from 30 to 60 °N (Fig. 3). Microbial emissions from tropical South America (9 ± 2 TgCH_4_ yr^−1^) and tropical Africa (7 ± 2 TgCH_4_ yr^−1^) drove the increase in our grid-scale inversion (Fig. 3l). Given that regions such as South America and Africa host extensive wetlands alongside growing populations, it is plausible that a combination of natural and anthropogenic processes has led to increases in tropical microbial emissions, consistent with previous studies^6,9^. During 2015–2016, biomass burning emissions increased by ~10 TgCH_4_ yr^−1^ due to large fires in mostly tropical Asia^39,40^. Our inverse model results are broadly consistent with independent estimates of fire emissions during 2015–2016 from tropical Asia^39,40^, and studies emphasizing the dominant role of tropical wetlands^15,17^. However, they contrast with those attributing the CH_4_ increase to an increase in fossil emissions^41^ or abrupt changes in OH^20^ (Supplementary Table S1).

From 2020 to 2022, global emissions increased by 29 ± 6 TgCH_4_ yr^−1^ with large increases from microbial emissions (39 ± 5 TgCH_4_ yr^−1^) and decreases from fossil emissions (−10 ± 6 TgCH_4_ yr^−1^) compared to 2017–2019 (Fig. 2g). The increase in microbial emissions appears to originate from North Africa (9 ± 2 TgCH_4_ yr^−1^), tropical Asia (8 ± 5 TgCH_4_ yr^−1^), and North America (6 ± 3 TgCH_4_ yr^−1^) (Fig. 3m, n). The decrease in fossil emissions was largest in temperate Eurasia (−7 ± 4 TgCH_4_ yr^−1^), followed by Europe (−2 ± 1 TgCH_4_ yr^−1^) and North America (−2 ± 1 TgCH_4_ yr^−1^). The 2020–2022 period includes reduced anthropogenic activities during the COVID-19 pandemic and the policy-driven decrease in fossil emissions in Eurasia^38,42–46^. The minor decreasing trends of fossil emissions in North America and Europe are consistent with decreased CH_4_ emissions intensity from the oil and gas industry and decreased coal emissions^46–48^. In conclusion, our isotopic inversion reveals an increasing impact of tropical microbial sources on atmospheric CH_4_ growth over time from 2000 to 2022, while fossil emissions showed no net or a negative contribution.

### Potential role of long-term trends in OH

Our Joint+OH sensitivity test shows the effects of  increases in tropospheric OH by a factor of 1.0 to 1.05 during 2000–2017, followed by a reduction to 1.02 during 2018–2022 (Supplementary Fig. S7)^5,21,35^. Inferred total emissions respond near-linearly to these OH perturbations (R^2^ of 0.9). Relative to the Joint scenario, the OH increase amplifies inferred CH_4_ emission growth between 2000–2006 and 2007–2013 by ~30% (25–32 TgCH_4_ yr^−1^), while the post-2017 OH reduction compresses inferred growth between 2017–2019 and 2020–2022 from 28 to 19 TgCH_4_ yr^−1^ (purple, Fig. 2; Supplementary Figs. S12-13). The elevated OH also shifts the spatial pattern of emissions, with the largest increases occurring in the tropics where OH concentrations are highest (Supplementary Figs. S7, S12- 13).

To remain consistent with observed *δ*^*1*3^C-CH_4_ trends, the higher OH during 2000–2013 requires a modest increase in the inferred fossil fuel contribution – from 27% to 31% of total emission growth over this period (Fig. 2e). This shift arises from a reduction in sink-weighted oxidative fractionation (Supplementary Table S4), with disequilibrium effects playing a negligible role (viz. Eq. (S5) of Lan et al.^27,49^). Conversely, during the post-2017 period where OH is reduced, a larger contribution from isotopically lighter microbial sources is required, driven by an increase in sink-weighted oxidative fractionation, again with negligible disequilibrium effects (Fig. 2). Overall, this sensitivity test demonstrates that while multi-year OH variability of up to 5% can meaningfully affect total inferred CH_4_ emissions, the impact on microbial-fossil partitioning remains small, shifting source attribution by less than 1% (Supplementary Fig. S14).

### Separating fossil and microbial emissions with isotopic constraints

Our CH_4_-only scenario, which lacks isotopic constraints, captures the changes in total emissions that are consistent with the Joint scenario (red in Fig. 2), as both inversions align with the observed changes in atmospheric CH_4_ (Fig. 1a). However, the scenarios differ in the partitioning of microbial and fossil emissions: the CH_4_-only scenario generally follows its prior (Supplementary Fig. S6a) and exhibits long-term increases in fossil emissions during 2000–2022 (Fig. 2c and Supplementary Figs. S15-16), consistent with previous CH_4_-only inversion studies^4^. The averaged fossil emissions are ~140 TgCH_4_ yr^−1^, which is larger than estimates of conventional CH_4_-only inversions^4^ of ~110 TgCH_4_ yr^−1^, as we used larger geologic seeps as priors^50^ for this paper. During 2020–2022, the CH_4_-only scenario exhibits a small change in fossil emission (0 ± 5 TgCH_4_ yr^−1^) while our isotopic inversions show a decrease of −10 ± 6 TgCH_4_ yr^−1^ (Figs. 3 and 2g). This could be related to reduced anthropogenic activities globally during the COVID-19 pandemic and policy-driven decreases in fossil emissions in Eurasia^38,42–46^, decreased CH_4_ emissions intensity from the oil and gas industry^46–48^, and decreased coal emissions from developed countries^48^. While previous studies have investigated the drivers of CH_4_ growth using satellite and in-situ data during this period^21–23^ (Supplementary Table S1), they have lacked isotopic constraints. Some other CH_4_-only inversions simulate an increase in fossil emissions during 2020–2022^4,29^, possibly because they rely more strongly on a prior inventory information to partition total fluxes. Our 2020–2022 results are driven by the large observed drop in atmospheric *δ*^*1*3^C-CH_4_ over this time^8,32^ (Fig. 1b). This demonstrates that isotopic inversions can independently quantify variations in microbial and fossil sources that CH_4_-only inversions do not always capture. Our uncertainty analysis (Supplementary Fig. S17a) shows that the introduction of *δ*^13^C-CH_4_ as a constraint reduces uncertainties for fossil and microbial emissions by 30% and 40%, respectively, at the global scale as well as reducing anti-correlation at continental scales (Supplementary Fig. S17b).

### Linking changes in wetland CH_4_ emissions to environmental changes with isotopic constraints

**Fig. 4:**
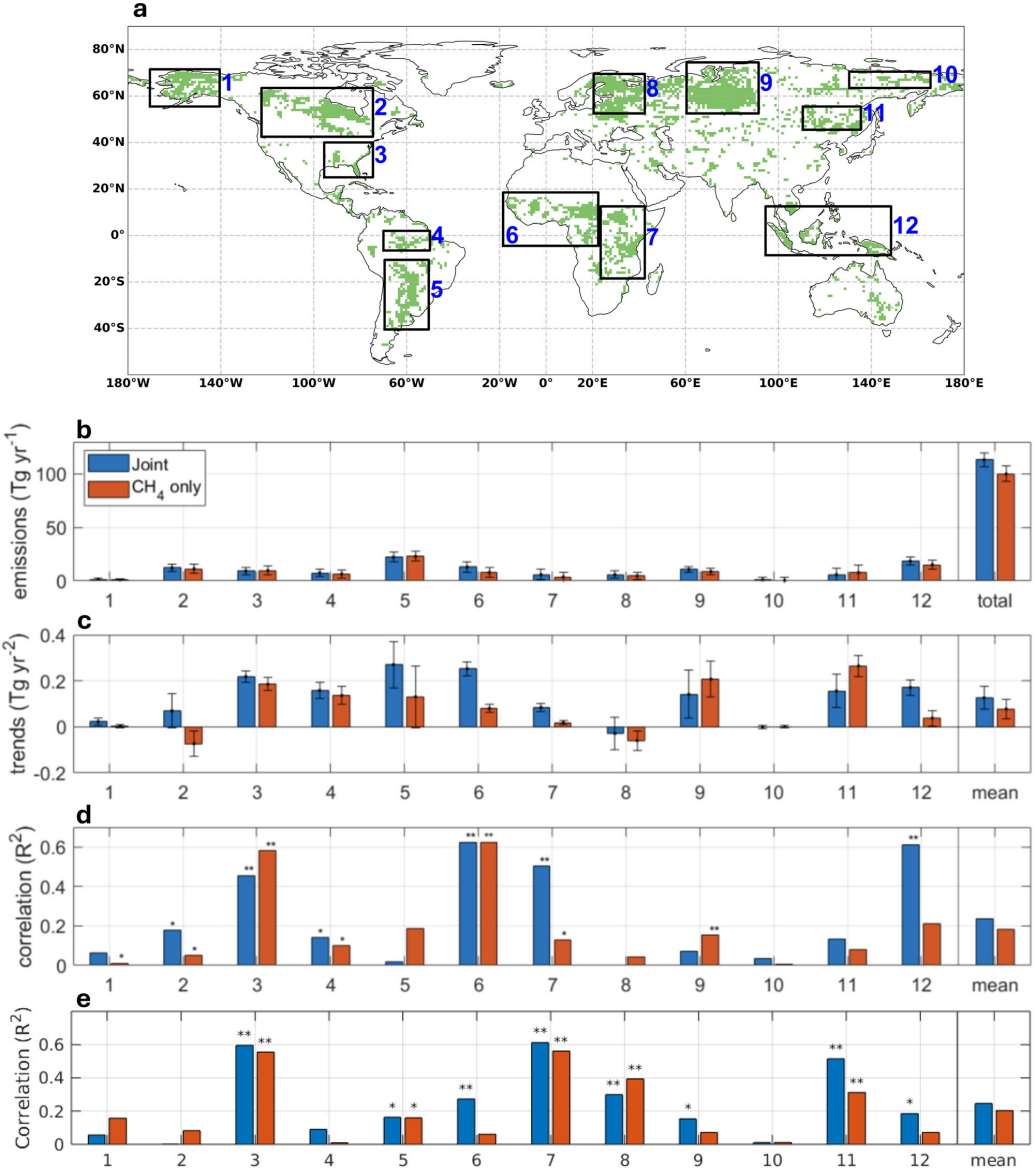
Mean and trends of optimized wetland CH_4_ emissions and their correlation with terrestrial water storage from 2003 to 2022. **a** Spatial map of major wetland regions (green): 1 = Alaska, 2 = Hudson Bay, 3 = Southeast US, 4 = Amazon, 5 = South America, 6 = West Africa, 7 = Southeast Africa, 8 = Europe, 9 = West Siberia, 10 = East Siberia, 11 = China, 12 = Southeast Asia. **b** Mean wetland emissions in TgCH_4_ yr^−1^ from wetland regions (panel **a**) and total (rightmost) from inversions with (blue) and without (red) isotopic constraints. Uncertainty is estimated as one standard deviation of the ensemble simulation results (Supplementary Information). **c** Emission trends in TgCH_4_ yr^−2^ by wetland regions (panel **a**) and the average (rightmost) for inversions with (blue) and without (red) isotopic constraints. Uncertainty from 1 standard deviation of the trends. **d**, **e** Coefficient of determination (R²) between regional wetland CH_4_ emissions and terrestrial water storage (cm) from major wetlands (panel **a**) and their R^2^ mean (rightmost) for inversions with (blue) and without (red) isotopic constraints using wetland priors from (**d**) static inundation and (**e**) dynamic inundation. Asterisks indicate significance (* <0.1, ** <0.05). Source data are provided as a Source Data file.

To better understand the potential drivers for the increase in microbial emissions we derive with atmospheric observations, we consider correlations between optimized wetland fluxes and environmental variables including temperature, precipitation, and terrestrial water storage (TWS) from 2003 to 2022 (Supplementary Information and Supplementary Figs. S18–20). We restricted our analysis to 12 major wetland regions based on the inundated wetland area (Fig. 4a)^51,52^, and focused on the period after 2003 due to the availability of TWS data^53^. We separated wetland and anthropogenic microbial sources by assuming that posterior microbial emissions are partitioned between wetlands and anthropogenic sources in the same proportion as the prior for each grid cell and each month (Supplementary Fig. S18), consistent with previous studies^17,22^. To assess the effect of the prior assumptions, we performed additional inversions using alternative wetland priors from a process-based model with dynamic inundation fractions^54^ (Supplementary Figs. S21–23). Although our optimized microbial CH_4_ emissions include both natural and anthropogenic sources, which *δ*^*1*3^C-CH_4_ alone cannot fully separate, wetland emissions should respond more directly to environmental drivers^54^. In contrast, anthropogenic sources such as ruminants, wastewater/landfills, and rice paddies are more likely to be influenced by management practices^55,56^.

We found that wetland emissions derived from the CH_4_-only scenarios are more sensitive to assumptions about wetland priors. Wetland emissions summed across the 12 major wetland regions are ~110 TgCH_4_ yr^−1^ when using static inundation and ~130 TgCH_4_ yr^−1^ when using the dynamic inundation product (Red in Fig. 4b and Supplementary Fig. S21b). The inversion with isotopic constraints produced less variation between 110 and 120 TgCH_4_ yr^−1^ with the alternate prior, highlighting the reduced sensitivity on prior assumptions when using *δ*^*1*3^C-CH_4_ data (Blue in Fig. 4b and Supplementary Fig. S21b).

Irrespective of prior, the Joint scenario shows larger trends in wetland emissions (Fig. 4c and Supplementary Fig. S21c). During 2003–2022, trends increased by an average of 0.13 and 0.14 TgCH_4_ yr^−2^ for isotopic inversions and 0.08 and 0.10 TgCH_4_ yr^−2^ for CH_4_-only inversions using the static and dynamic inundation priors, respectively. We observed the largest trend increases in tropical wetland regions in the Amazon, South America, West Africa, and Southeast Asia (regions 4, 5, 6, and 12, respectively). These larger trends in wetland emissions using isotopic constraints reflect the fact that the isotopic inversions attribute more of the atmospheric CH_4_ rise to microbial sources (Fig. 2).

The larger trends of wetland emission in the isotopic inversions correlate better with the interannual variability of TWS in major wetland regions, for both wetland priors (Fig. 4d, e, Supplementary Fig. S21d and Supplementary Table S5). For static-inundation priors, the average correlation between optimized wetland emissions and TWS increases by 30% in the isotopic inversions relative to the CH_4_-only case (Fig. 4d), and the overall improvement was consistent (20%) when using dynamic-inundation priors (Fig. 4e). In particular, the Amazon, Southeast Africa, North China, and Southeast Asia display consistently stronger correlations with TWS in the isotopic inversions compared to the CH_4_-only scenario (regions 4, 7, 11, 12 in Fig. 4d, e). Statistical tests using the cocor framework and Williams’ test^57^ further confirm that these improvements are robust, demonstrating significantly stronger correlations with TWS when isotopic constraints are included (Supplementary Fig. S23). Arctic wetland regions in general exhibit weaker correlations with TWS but stronger correlations with precipitation or temperature (regions 1, 9, 10 in Fig. 4d and Supplementary Table S5), likely due to complex hydrological dynamics influenced by glacier melt^58^ and permafrost thaw^59^, and the limited ability of the GRACE satellite that measures TWS to detect signals at high latitudes^60^.

We show that incorporating isotopic constraints improves the correlation between long-term wetland CH_4_ emissions and TWS, highlighting the value of isotopic data in source attribution largely independent of prior assumptions. The strong observed correlations between optimized wetland CH_4_ fluxes and TWS suggest that wetland emissions are a major component of atmospheric CH_4_ growth, especially for hot and humid regions. Specifically, our isotopic inversion indicates that ~40% of the global CH_4_ increase between 2003–2005 and 2020–2022 can be associated with increased natural microbial emissions (Supplementary Fig. S18b).

## Discussion

Attributing the recent CH_4_ increase to different sources using atmospheric measurements is crucial for understanding the anthropogenic forcing and natural climate feedback response of CH_4_ emissions. Specifically, natural microbial emissions remain the most uncertain source of global CH_4_, and these have been estimated to be ~30% of the global CH_4_ budget (Supplementary Fig. S1)^61^. Increasing temperature and CO_2_ increase may accelerate natural CH_4_ emissions, accounting for a portion of the atmospheric CH_4_ increase^59,62^. Recent studies also emphasize the role of the intensification of natural climate forcing in the large CH_4_ increase during 2020–2022^8,22,54^. Isotopic data provides valuable information on the drivers of atmospheric CH_4_ increases, allowing us to understand the fractional contribution of different sources to the growth in atmospheric CH_4_, independently of estimates based on emission inventories.

In this study, we analyzed the causes of three rapid growth periods during 2007–2013, 2014–2017, and 2020–2022 using our dual tracer inversion constrained by in-situ CH_4_ and *δ*^*1*3^C-CH_4_ measurements from 2000 to 2022. Our findings indicate an acceleration of CH_4_ emissions from 2000 to 2022. Inversions run with enhanced OH abundance and variability showed proportionally greater emissions during this period, but the relative partitioning between microbial and fossil emissions was insensitive to OH. During 2007–2013, both microbial and fossil emissions increased, with the largest contribution from temperate Eurasia. During 2014–2017, microbial emissions drove the increase in our simulation, mostly from the tropics in South America and Africa. From 2020 to 2022, microbial emissions surged while fossil emissions declined, a decrease not captured by inversions without isotopic constraints. Isotopic inversions also show larger trends over wetland regions during 2003–2022 and strong correlations with TWS, supporting the attribution of total microbial emissions, at least in part, to climate-driven natural microbial emissions.

There are limitations to this study. Improving our ability to understand CH_4_ sources and sinks using atmospheric observations will require better representation of different ecosystems, especially the tropics and Arctic (Supplementary Fig. S5). Incorporating satellite retrievals will significantly enhance the spatial and temporal coverage of observations, although CH_4_ remote sensing is limited in the tropics and the Arctic due to extensive cloud cover and low sun angles^29,63^. While we used prior information to differentiate between natural and anthropogenic microbial sources, which *δ*^*1*3^C-CH_4_ cannot fully separate^26^, measurements of co-emitted species with CH_4_, such as ethane and ammonia, could provide useful additional constraints on sources^30,64^. Moreover, measurements of the hydrogen, radiocarbon, and clumped isotopes of CH_4_ could further help separate sources as well as atmospheric sinks by providing independent constraints on the CH_4_ budget because they are sensitive to different components of sources and sinks^31,65^. Lastly, limited understanding of OH variability and its long-term trend remain a key uncertainty in attributing CH_4_ growth, underscoring the need for further studies using independent tracers^66,67^.

## Methods

This work contributes to the ongoing development and improvement of NOAA’s atmospheric CH_4_ assimilation system, the CarbonTracker-CH_4_^68,69^ (Supplementary Fig. S4), but the specific scenarios we present in this paper and the analyses on sensitivity and correlation have not been published elsewhere. This inversion system incorporates (1) prior estimates of microbial, fossil, and pyrogenic emissions and their isotopic signatures together with atmospheric chemical and soil sinks (Methods 1), (2) in situ observations of atmospheric CH_4_ and *δ*^13^C-CH_4_ (Methods 2), and (3) a 4DVar numerical inversion framework based on TM5 atmospheric transport model (TM5 4DVar) that optimizes surface fluxes to reconcile model predictions with the observations at the monthly and grid-level scales (Methods 3). More details on our prior sensitivity test, ensemble simulation, and correlation analyses can be found in Supplementary Information.

As is typical, *δ*^13^C-CH_4_ is defined as *δ*^13^C = (*R*_*sam*_/*R*_*std*_ - 1)×1000 ‰ (Eq. (1)), where the ratio of the heavy isotope to the light isotope in the sample (R_sam_ = (^13^C/^12^C)_sam_) relative to a known standard ratio of the standard, R_std_, which is for Vienna Pee Dee Belemnite (VPDB).

### Prior source and sink setup

Prior estimates of emissions from microbial, fossil, and pyrogenic sources are based on emissions inventories and process-based models at 1-degree resolution (Supplementary Figs. S1, S6, Supplementary Table S3). For anthropogenic microbial emissions, we use gridded emissions from EDGAR 8.0 as prior estimates for emissions from rice agriculture, enteric fermentation, animal waste management, wastewater, and landfills^70^. For wetland emissions, we use the gridded wetland emissions of CH_4_ from a process-based model, a Terrestrial Ecosystem Model (TEM)^71,72^, with global static wetland distribution^52^. Prior fossil fuel-emissions are from EDGAR 8.0 gridded at 1-degree with annual resolution^70^ and geologic fossil emissions, also at 1-degree annual resolution, are from Etiope et al.^50^. Biomass and biofuel burning emissions, come from the Global Fire Emission Database (GFED) 4.1 s for 1997–2022^73^. The spatial and temporal variability of *δ*^13^C-CH_4_ isotopic signatures from emissions are as in Basu et al. and Lan et al.^7,27^ (Figs. S2, 3 and Tables S2-3).

We prescribe (and do not optimize) atmospheric CH_4_ oxidation via OH and Cl throughout the atmosphere, by O(^1^D) in the stratosphere, and via microbes in aerobic soils (Supplementary Table S4 and Supplementary Fig. S1). For tropospheric OH, we use the monthly OH climatology of Spivakovsky et al.^74^ after scaling by 0.9 to match the declining atmospheric abundance of methyl chloroform in the early 2000s (Supplementary Fig. S7b)^7,75^. For the Joint+OH scenario, we then applied an annual scaling factor, increasing tropospheric OH to ~1.05 from 2000 to 2017, followed by a decrease to ~1.02 from 2018 to 2022, based on LMDz-INCA (Supplementary Fig. S7a)^5,21,35^. Monthly climatological CH_4_ loss rates in the stratosphere due to OH, Cl, and O(^1^D) were constructed from a run of the ECHAM5/MESSy1 chemistry transport model^76^. Loss due to tropospheric Cl is simulated using a model-derived estimate of tropospheric Cl^77^. We use the gridded soil oxidation of CH_4_ from the Terrestrial Ecosystem Model (TEM)^72,78^. All CH_4_ oxidants react preferentially ^12^CH_4_ relative to ^13^CH_4_ (Supplementary Fig. S2). The ratio of rate constants for these reactions is modeled using *k*_*12*_*/k*_*13*_ = *1/α* = *Ce*^*D/T*^ (Eq. (2)), where *T* is air temperature (K), and the coefficients *C* and *D* used in the model are listed in Supplementary Table S4.

### Observations

To maximize the spatiotemporal coverage of atmospheric data, we used a database of CH_4_ mole fraction measurements from the NOAA Global Greenhouse Gas Reference Network and University of Colorado Institute for Arctic and Alpine Research (INSTAAR) harmonized with those from 30 other laboratories around the world (Supplementary Fig. S5)^33^. These data have been quality-checked and converted to the World Meteorological Organization (WMO) X2004A scale^79^.

We used *δ*^13^C-CH_4_ data from INSTAAR as well as other isotope laboratories making precise measurements of atmospheric CH_4_ with isotope ratio mass spectrometers^80^. Although all of these laboratories report their measurements relative to VPDB, their standardization approach varies and may not agree to better than 0.1 per mil. Thus, we applied empirical offsets^34^ to bring the *δ*^13^C-CH_4_ data onto the INSTAAR realization of VPDB. The total measurement uncertainty in assimilated *δ*^13^C-CH_4_ datasets was typically less than 0.15 ‰. For the data selection and filtering, we follow the same rules as Basu et al.^7^.

We set the model-data mismatch for CH_4_ based on Bruhwiler et al.^81^. Specifically, the model–data mismatch errors for CH_4_ were based on the evaluation of forward simulation with prior fluxes by selecting the one standard deviation of prior bias (prior - observed) for each surface site. For aircraft profiles, model-data mismatch error was determined using the one standard deviation of the prior bias at every 1 km vertical interval. The inversion closely matches remote marine background sites due to its small model-data mismatch while some urban sites were given a very large model–data mismatch because they are likely influenced by strong local sources, therefore exhibiting large standard deviation of prior bias (Supplementary Table S6).

The model-data mismatch errors of *δ*^13^C-CH_4_ were calculated as the quadrature sum of measurement errors and model errors following Basu et al.^7,82^. We calculated model error using the spatial gradient of prior-simulated *δ*^13^C-CH_4_ at the sites, using horizontally and vertically adjacent model grid cells. More information on the participating laboratories and their datasets, as well as site-specific model–data mismatches and the selection of assimilation and validation sites for atmospheric CH_4_ and *δ*^13^C-CH_4_ data, is provided in Supplementary Table S6.

### TM5 4DVar optimization

We use the TM5 4DVar inversion framework^83^, which has been used to estimate surface fluxes of CO, CO_2_, and CH_4_^84–87^ in single-tracer inversions, as well as source-specific CO_2_ fluxes in multi-tracer inversions^88,89^. Two tracers are simulated in our inversion: total CH_4_ or *C* and the artificial tracer *C x δ’*, where *δ’* = (^13^CH_4_/CH_4_)/R_std_-1 (Eq. (3)), following Basu et al.^7^.

TM5 4DVar minimizes a cost function *J(x)* = 1/2 (*Hx*−*y*)^T^*R*^−1^(*Hx*−*y*) + 1/2(*x*−*x*_*0*_)^T^*B*^−1^(*x*−*x*_*0*_) (Eq. (4)), as a function of state vector *x* (posterior fluxes) by balancing fits to atmospheric observations *y* with deviations from the prior fluxes *x*_0_, where *H* is the transport, chemistry, and observation operator connecting surface fluxes with atmospheric measurements, and *R* and *B* are the error covariances of *Hx* − *y* and prior fluxes, respectively; *R* was set based on the model-data mismatch calculations described above. The state vector x (posterior fluxes) is composed of microbial, fossil, and pyrogenic sources in each 3° × 2° land grid cell, with a monthly resolution. Our prior error covariance matrix (B) follows that of Basu et al.^7^. The cost function *J* is minimized over 100 iterations by an M1QN3 minimizer^5^.

Due to the long computation time for inversion, we split our simulation periods into 8 inversions that were run in parallel. Eight 5-year inversions were run simultaneously with 2 years of overlap (first and last years) between inversions, starting in 1997, 2000, 2003, 2006, 2009, 2012, 2015, and 2018. After all eight inversions finished, the fluxes from the middle 3-year period of each inversion were considered for analysis. For simulating prior and posterior mole fractions, fluxes from the non-overlapping periods were stitched together and a single forward run was done with those long-term posterior fluxes^7^. To quantify the effect of stitching, we ran the inversion with climatological priors in sequence by repeating a single annual cycle of emissions in 2006 during 2006–2022 (Supplementary Figs. S24-25).

### Scenarios

We compare three scenarios in this paper. First, the “CH_4_-only” scenario represents the conventional inversion approach that does not use atmospheric δ^13^C-CH_4_ data. The priors for this conventional scenario, we used source and sink estimates as they were from inventory and process-based models (Supplementary Fig. S6a and Supplementary Tables S3-4). When optimizing fluxes using observations and Eq. (3), we did not assimilate δ^13^C-CH_4_ data but instead used them for evaluation.

Second, the “Joint (CH_4_ and δ^13^C-CH_4_)” scenario incorporates both CH_4_ and δ^13^C-CH_4_ data to constrain global CH_4_ budgets. To prevent spin-up artifacts associated with isotope ratios^90^, the priors for the Joint scenario satisfy global mass balance requirements for CH_4_ and δ^13^C-CH_4_, ensuring consistency with the global annual growth of atmospheric CH_4_ and the long-term mean of δ^13^C-CH_4_ (Supplementary Fig. S6b and Supplementary Information)^27^. When optimizing fluxes, we assimilated both CH_4_ and δ^13^C-CH_4_, and we evaluated performance using unassimilated (validated) CH_4_ and δ^13^C-CH_4_ observations (Supplementary Fig. S9).

Lastly, the “Joint + OH” scenario is designed to investigate long-term changes and interannual variability in OH. It builds upon the setup of the “Joint” scenario, with the additional inclusion of annual variability in tropospheric OH based on the chemical transport model, LMDz-INCA^5,21,35^ (Supplementary Fig. 7a).

### Reporting summary

Further information on research design is available in the Nature Portfolio Reporting Summary linked to this article.

## Supplementary information

Supplementary Information

Reporting Summary

Transparent Peer Review file

## Source data

Source Data Figure 1

Source Data Figure 2

Source Data Figure 3

Source Data Figure 4

## Data Availability

The CH_4_ and *δ*^13^C-CH_4_ data used for the atmospheric inversion modeling can be downloaded from 10.15138/T4MZ-2Z29 (Lan et al.)^33^. The isotopic signatures can be downloaded from 10.15138/qn55-e011 (Sherwood et al.)^27,65^. The EDGAR v8.0 time series of country-level emissions and sector-specific grid maps are from: https://edgar.jrc.ec.europa.eu/dataset_ghg80^70^. The ERA5 reanalysis data are from: https://www.ecmwf.int/en/forecasts/dataset/ecmwf-reanalysis-v5^91^. The monthly fire emissions from Global Fire Emissions Database version 4.1 are from: https://www.geo.vu.nl/~gwerf/GFED/GFED4/^92^. The global water storage/height anomalies from GRACE and GRACE-FO are from: 10.5067/TEMSC-3JC63^53^. Source data are provided with this paper.

## Code availability

The TM5 4DVar code for performing the atmospheric inversion is publicly available at https://sourceforge.net/p/tm5/cy3_4dvar/ci/default/tree/. We provide TM5 4DVar user guide in Supplementary Note 1.
