# Supplementary material for: Isotopic constraints in methane inversions reveal larger trends in wetland emissions with improved linkage to terrestrial water storage
Source: Nat Commun. 2026 Jun 29;17:8112. doi: 10.1038/s41467-026-74777-4 (PMC13458832; doi:10.1038/s41467-026-74777-4)
Supplement: Supplementary file 1 [file 41467_2026_74777_MOESM1_ESM.pdf]

Supplementary Information for

Isotopic constraints in methane inversions reveal larger trends in wetland emissions with improved linkage to terrestrial water storage

Youmi Oh*[1,2], Lori Bruhwiler*[2], Xin Lan[1,2], Santanu Halder[1,2], Ben Riddell-Young[1,2], Sourish Basu[3], John B. Miller[2], Sylvia Michel[4,5], Ken Schuldt[1,2], Arlyn Andrews[2], Sebastien Biraud[6], Łukasz Chmura[7,8], Tatiana Di Iorio[9], Elise-Andree Guerette[10], Elena Kozlova[11], Licheng Liu[12], Simonetta Montaguti[13], Shinji Morimoto[14], Martin Steinbacher[15], Taku Umezawa[14,16], Irène Xueref-Remy[17], Giulia Zazzeri[18]

[1] Cooperative Institute for Research in Environmental Science, University of Colorado, Boulder, CO, USA
[2] Global Monitoring Laboratory, National Oceanic and Atmospheric Administration, Boulder, CO, USA
[3] Earth System Science Interdisciplinary Center, University of Maryland, College Park, MD, USA
[4] Institute for Arctic and Alpine Research, University of Colorado, Boulder, CO, USA
[5] Department of Atmospheric and Oceanic Sciences, University of Colorado, Boulder, CO, USA
[6] Climate & Ecosystem Sciences Division, Lawrence Berkeley National Laboratory, Berkeley, CA, USA
[7] AGH University of Krakow, Faculty of Physics and Applied Computer Science, Krakow, Poland
[8] Institute of Meteorology and Water Management — National Research Institute, Warsaw, Poland
[9] Italian National Agency for New Technologies, Energy, and Sustainable Economic Development (ENEA), Rome, Italy
[10] Environment, Commonwealth Scientific and Industrial Research Organisation (CSIRO), Aspendale, VIC, Australia
[11] Department of Earth System Science, University of Exeter, Exeter, UK
[12] Department of Bioproducts and Biosystems Engineering, University of Minnesota, St. Paul, MN, USA
[13] National Research Council of Italy, Institute of Atmospheric Sciences and Climate (ISAC), via Gobetti 101, I-40129, Bologna, Italy
[14] Center for Atmospheric and Oceanic Studies, Graduate School of Science, Tohoku University, Sendai, Japan
[15] Empa, Swiss Federal Laboratories for Materials Science and Technology, Duebendorf, Switzerland
[16] National Institute for Environmental Studies, Tsukuba, Japan
[17] Institut Méditerranéen de Biodiversité et Ecologie Marine et Continentale (IMBE), Aix-Marseille Université, CNRS, Institut de Recherche pour le Développement (IRD), Avignon Université, 13290 Aix-en-Provence, France
[18] Research on the Energy System - RSE S.p.A., Via R. Rubattino 54, 20134 Milano, Italy

*Authors for correspondence:

Youmi Oh
Email: youmi.oh@noaa.gov

Lori Bruhwiler
Email: lori.bruhwiler@noaa.gov

Contents of this file: Supplementary Methods S1-3, Supplementary Figures 1-25, Supplementary Tables 1-6, references, and Supplementary Note 1.

Supplementary Methods

1. Prior Global Mass Balance and Sensitivity tests

Modeling δ^{13}C-CH_4 in the atmosphere requires special care to spin up the model to a quasi-steady state to avoid initial condition artifacts during the analysis period[1]. The time required for atmospheric δ^{13}C-CH_4 to reach a steady state can be significantly longer than even the CH_4 lifetime of ~9 years, depending on the size of the atmospheric CH_4 burden and how far off the initial δ^{13}C-CH_4 is. Thus, we spun up our model for 14 years from 1984 to 1999, and selected 2000–2022 as the analysis period. To further avoid the initial condition artifacts, we adjust our prior to satisfy global mass balance requirements for CH_4 and δ^{13}C-CH_4 for **Joint** and **Joint+OH** scenarios[2] (Fig. S6b). Specifically, we scale the prior fossil emissions to reach a total of ~145 TgCH_4 yr^{-1} from all fossil sources, and impose a linear trend on microbial emissions to achieve an increase of ~70 TgCH_4 yr^{-1} in total 2022 emissions compared to 1999–2006. This ensures that our global CH_4 and δ^{13}C-CH_4 budgets approximate the long-term trends in atmospheric CH_4 and δ^{13}C-CH_4 during our simulation period.

To evaluate the effects of prior assumptions, we also conducted **CH_4-only** and **Joint** sensitivity simulations using climatological priors to run the inversion in sequence (Fig. S24). These priors assumed the same spatial and temporal prior distribution from 2006 for all subsequent years (2007–2021). The climatological priors progressively deviated from observations over time (Fig. S25), as they did not account for the significant changes in atmospheric CH_4 and δ^{13}C-CH_4 after 2007. The model-data comparisons with climatological priors diverge from isotope observations (0.15‰ for Joint and 0.4‰ for CH_4-only scenarios) (Fig. S25). As such, if prior emissions do not follow atmospheric trends, one must consider long adjustment times needed for δ^{13}C-CH_4 to reach a quasi-steady state[1,2]. We addressed this issue by ensuring that our prior global total emissions were consistent with global atmospheric trends of CH_4 and that the fraction of fossil and microbial sources matched the averaged δ^{13}C-CH_4 from observations (Fig. S6), unlike other isotope inversion studies[3,4]. This allows the inversion to use the observations to allocate emissions from different sources spatially and temporally.

2. Ensemble simulation for posterior uncertainty and correlation analysis between fossil and microbial emissions

We estimated the uncertainty of our simulation using 100 ensemble members with different random uncertainties in prior flux and observations[5]. The random uncertainties are associated with components of the inversion system whose errors are assumed to be zero on average. We construct the ensembles with prior fluxes and observations perturbed according to the covariances specified by B and R, respectively. With 100 ensemble members, our estimate is expected to be within 10 % of the exact analytical solution.

Previous studies showed that additional isotope information can disentangle different sources, but this depends on the isotope measurement coverage and atmospheric transport connecting the emissions to the simulated concentrations[5]. To quantify this, we evaluated the posterior correlation between microbial and fossil at regional, global, and annual scales, calculated from our 100-member ensemble of independent inversions. Our correlation analysis further confirms that Joint inversion is stronger at separating fossil and microbial sources at continental scales (Fig. S17b), where we have a relatively large number of isotope measurements (Fig. S5). Because uncertainties in annual-mean emissions are very small (Fig. S17a), correlations that vary within these uncertainties can yield only modest apparent improvements over the tropic regions with sparse measurements. Nonetheless, within 30 °S - 30 °N, we include 30 sites that provide regular, high-precision $\delta^{13}C\text{-}CH_4$ observations (Table S6). These sites contribute approximately 15,000 in-situ surface measurements between 2000 and 2022, about one-third of the total $\delta^{13}C\text{-}CH_4$ dataset used here. Therefore, despite uneven spatial coverage, the $\delta^{13}C\text{-}CH_4$ dataset we use provides substantial information for source attribution in the tropics, particularly for long-term trends. This is reflected in our correlation analyses with terrestrial water storage: regardless of wetland priors, isotopic inversions yield larger wetland emission trends and stronger correlations with terrestrial water storage (Figs. 4 and S19–23).

3. Correlation between optimized wetland emissions and environmental parameters

We have performed a correlation analysis to investigate the relationship between monthly wetland CH_4 emissions, terrestrial water storage (TWS) anomalies, and climate parameters (such as 2m temperature and total precipitation) during 2003-2022 over major wetland-dominated regions (Fig. 4a).

We have obtained a monthly global map of land TWS anomalies from NASA's Gravity Recovery and Climate Experiment (GRACE during 2002-2017) and GRACE Follow On (GRACE-FO; afterward) version RL06.3Mv04 for the time period 2003-2021[6]. GRACE detects small variations in Earth's gravity due to changes in the surface mass by a paired satellite located at 220 km and 330 km, respectively. TWS anomalies represent a measure of the vertical distribution of land water storage, including several components (such as snow, surface water, soil moisture, and groundwater). Monthly TWS anomalies at each grid cell were derived as a deviation from a temporal average during 2004-2009.

The monthly re-analysis global spatial map of climate parameters i.e., 2m temperature and total precipitation, is obtained from the European Centre for Medium-Range Weather Forecasts (ECMWF) during 2003-2021[7,8]. 2m temperature is a post-process product obtained from a non-linear interpolation that is calculated between the forecasted model lowest level temperature and the model-forecasted temperature at the earth's surface. The total precipitation is calculated using a data-driven ECMWF system.

Further, we have aggregated wetland CH_4 emissions, TWS, and climate parameters over major wetland-dominated regions at an annual scale (Fig. S19-20). Pearson correlation coefficients were calculated

between wetland CH_4 emissions, TWS, and climate parameters (Table S5), and linear regression was applied to estimate trends and their associated uncertainties. These analyses were first conducted using wetland priors with a static inundation map (Figs. S19–20) and were then repeated using an alternative wetland prior with the LPJ process-based model and dynamic inundation[9] (Figs. S21–22) to assess the sensitivity of regional relationships to assumptions about wetland distribution. To evaluate whether correlations differed significantly between the Joint and CH_4-only inversion scenarios, we applied formal statistical tests for dependent correlations using the cocor framework and Williams' test (Fig. S23)[10,11].

Supplementary Figures

Figure S1 Pie chart of global source and sink estimation over 2000-2022. (a) Pie chart of global mean CH_4 emissions from anthropogenic and natural microbial, fossil, and pyrogenic source sectors based on the results from the "Joint" inversion presented in this study. (b) Pie chart of global mean CH_4 oxidation from tropospheric, stratospheric, and terrestrial sink sectors. The percentage of source and sink sectors is based on our prior setup (Table S3-4).

Figure S2 Global mean isotopic signature of sources and fractionation of sinks. (a) Global mean flux-weighted CH_4 isotopic signature ($\delta^{13}C$-CH_4) of microbial (green), fossil (yellow), and pyrogenic (red) sources. Approximate values of global mean source signatures are shown (Table S2), and the size of each bar reflects the relative flux from each source category. The flux-weighted source signature across all sources is estimated to be ~—56‰, and the difference between this value and the atmospheric signal (~—47‰; "Atm. $\delta^{13}C$-CH_4") is attributed to process-weighted sink fractionation (~9‰), as illustrated in panel (b). (b) The global mean process-weighted isotopic signature of atmospheric chemical sinks from OH (dark blue), Cl (dark magenta), and O^1D (blue), as well as soil sinks (orange). The size of each bar approximates the relative sink strength, although the OH sink is much larger than the other sinks (Fig. S1).

Figure S3 Flux-weighted probability distribution of source signatures. Histogram of flux-weighted probability distribution function of $\delta^{13}C$-CH_4 from microbial (blue), fossil (yellow), and pyrogenic (green) sources.

Figure S4. Schematic diagram of CarbonTracker-CH_4 atmospheric inversion system. The goal of the CarbonTracker-CH_4 is to produce quantitative estimates of methane (CH_4) emissions at the Earth's surface that are consistent with observed patterns of CH_4 and its stable carbon isotope ratios ($\delta^{13}C$-CH_4) in the atmosphere. (Methods 1) Prior information on sector-specific CH_4 source fluxes and their signatures and isotopic fractionation by chemical and soil sinks and their sink size is prescribed. (Method 2) In-situ observations of atmospheric CH_4 and $\delta^{13}C$-CH_4 provide the observational constraints. (Method 3) These priors and observations are combined within a dual-tracer inversion system using the TM5 atmospheric transport model (TM5 4DVar), which simulates transport driven by meteorology and optimizes prior surface emissions from microbial, fossil, and pyrogenic sources. Differences between modeled and observed atmospheric fields are attributed to errors in prior emissions and are minimized through the inversion to produce posterior flux estimates. The optimized outputs include global three-dimensional atmospheric CH_4 mole-fraction fields and monthly posterior flux estimates for microbial, fossil, and pyrogenic sources.

Figure S5. Global distribution of in-situ CH_4 and $\delta^{13}C$-CH_4 used in this study. Mean locations of measurements for CH_4 (blue) and $\delta^{13}C$-CH_4 (red) from surface flask samples (circle), surface hourly and continuous high-frequency data from tower (square), and flask and continuous high-frequency data from aircraft platforms (diamond). More information on the participating laboratories and their datasets, as well as site-specific model–data mismatches and the selection of assimilation and validation sites for atmospheric CH_4 and $\delta^{13}C$-CH_4 data, is provided in Table S6.

Figure S6 Global annual emissions of sources. Long-term annual emissions (in $TgCH_4\ yr^{-1}$) of total microbial, fossil, and pyrogenic sources from each source sector (a) before and (b) after the global mass balance of atmospheric CH_4 and $\delta^{13}C$-CH_4 (Supplementary Information and Table S3).

Figure S7 Annual scaling factor and distribution of surface OH. (a) Annual scaling factor of tropospheric OH for the Joint-OH scenario during 2000-2022. The estimated scaling factor is based on an atmospheric chemistry model, LMDz-INCA[3,12,13]. (b) Global distribution of OH at the surface level from

Spivakovsky et al. (2000)[14] after scaling by 0.9 to match the declining atmospheric abundance of methyl chloroform in the early 2000s[5,15].

Figure S8 Summary of residuals of assimilated CH_4 and $\delta^{13}C$-CH_4. Residuals for (a) CH_4 and (b) $\delta^{13}C$-CH_4 of assimilated data that are used to optimize CH_4 flux during the atmospheric inversion. Simulated minus observed values (CH_4 in nmol mol^{-1} (ppb), $\delta^{13}C$-CH_4 in ‰) are as a function of time and latitude for prior (top left) and posterior estimation from Joint (top right), Joint+OH (bottom left), and CH_4-only (bottom right scenarios). Each dot represents the time and location of an observation, and the legend shows mean bias (μ), standard deviation of bias (σ), and the chi-squared value (χ^2). The χ^2 value is defined as the average squared bias normalized by the squared model-data mismatch and provides a measure of goodness of fit, with values close to 1 indicating the model is not over- or under-fitting.

Figure S9 Summary of residuals of unassimilated CH_4 and $\delta^{13}C$-CH_4. Residuals for (a) CH_4 and (b) $\delta^{13}C$-CH_4 of unassimilated data that are not used to optimize CH_4 flux during the atmospheric inversion for model validation. Simulated minus observed values (CH_4 in nmol mol^{-1} (ppb), $\delta^{13}C$-CH_4 in ‰) are as a function of time and latitude for prior (top left) and posterior estimation from Joint (top right), Joint+OH (bottom left), and CH_4-only (bottom right scenarios). Each dot represents the time and location of an observation, and the legend shows mean bias (μ) and standard deviation of bias (σ).

Figure S10 Regional observed and simulated long-term trends in atmospheric CH_4. Observed and simulated averaged CH_4 in ppb for Arctic (a), tropics (b), and polar southern hemisphere (c) regions in 2000-2022. For each sub-plot in (a)-(c), (top) time series of averaged values and (bottom) difference between simulated and observed values for observed (black), prior (dark green), and posterior from Joint (blue), CH_4-only (red), and Joint+OH (purple) scenarios.

Figure S11 Regional observed and simulated long-term trends in atmospheric $\delta^{13}C$-CH_4. Observed and simulated averaged CH_4 in ppb for Arctic (a), tropics (b), and polar southern hemisphere (c) regions in 2000-2022. For each sub-plot in (a)-(c), (top) time series of averaged values and (bottom) difference between simulated and observed values for observed (black), prior (dark green), and posterior from Joint (blue), CH_4-only (red), and Joint+OH (purple) scenarios.

Figure S12 Global and regional trends in emissions during 2000-2022 for Joint+OH Scenario. (a-d) Spatial distribution of trends in long-term CH_4 emissions in Gg yr^{-2} from (a) total, (b) microbial, (c) fossil, and (d) pyrogenic sources during 2000-2022 for Joint+OH scenario. (e-k) Long-term trends in regional CH_4 emissions from (e) North America, (f) South America, (g) Africa, (h) Europe, (i) Asia, and (j) Australia from microbial (Blue), fossil (red), pyrogenic (yellow), and total (purple) sources. Uncertainty is estimated from the ensemble simulation (Supplementary Information).

Figure S13 Differences in regional CH_4 emissions for the Joint+OH Scenario. Differences in regional CH_4 emissions between (a) 2000-2006 and 2007-2013, (b) 2010-2013 and 2014-2017, (c) 2018-2019 and 2020-2021, and (d) 2017-2019 and 2020-2022 in $TgCH_4\ yr^{-1}$ from North America (blue), South America (red), Africa (yellow), Asia (purple), and Europe (skyblue) continents. Uncertainty is estimated from the ensemble simulation (Supplementary Information).

Figure S14 Emission ratios for Joint and Joint+OH scenarios. The ratio of fossil emissions to total emissions (light blue) and microbial emissions to total emissions (green) in the Joint (solid lines) and Joint+OH scenarios (dashed lines). Changes in emission ratios between the two scenarios never exceeds 1% of total emissions for any year.

Figure S15 Global and regional trends in emissions during 2000-2022 for CH_4-only Scenario. (a-d) Spatial distribution of trends in long-term CH_4 emissions in $GgCH_4\ yr^{-2}$ from (a) total, (b) microbial, (c)

fossil, and (d) pyrogenic sources during 2000-2022 for CH_4-only scenario. (e-k) Long-term trends in regional CH_4 emissions from (e) North America, (f) South America, (g) Africa, (h) Europe, (i) Asia, and (j) Australia from microbial (Blue), fossil (red), pyrogenic (yellow), and total (purple) sources. Uncertainty is estimated from the ensemble simulation (Supplementary Information).

Figure S16 Differences in regional CH_4 emissions for the CH_4-only Scenario. Differences in regional CH_4 emissions between (a) 2000-2006 and 2007-2013, (b) 2010-2013 and 2014-2017, (c) 2018-2019 and 2020-2021, and (d) 2017-2019 and 2020-2022 in $TgCH_4\ yr^{-1}$ from North America (blue), South America (red), Africa (yellow), Asia (purple), and Europe (skyblue) continents. Uncertainty is estimated from the ensemble simulation (Supplementary Information).

Figure S17 Spatial uncertainty and correlation between microbial and fossil emissions. (a) Global and regional uncertainty of total, microbial, fossil, and pyrogenic emissions from ensemble simulations (Supplementary Information). (b) Posterior correlation between global annual microbial and fossil methane emissions for Scenarios Joint (Blue) and CH_4-only (Red) for global and regional scales (North America, US, South America, Africa, Asia, East Asia, Australia, and Europe).

Figure S18 Global annual emissions of sources from prior and posterior simulation for the Joint scenario. Long-term annual emissions (in $TgCH_4\ yr^{-1}$) of microbial, fossil, and pyrogenic sources from each source sector from (a) mass-balanced priors (same as Fig. S6b) and (b) the inversion posterior.

Figure S19 Temporal Variations between optimized wetland emissions and terrestrial water storage (TWS) from 2003 to 2022. A correlation analysis between regional optimized wetland CH_4 emissions (in $TgCH_4\ yr^{-1}$) and TWS (in cm) from major wetland regions (Fig. 4a) for CH_4-only (red dashed) and Joint scenario (red solid). Each panel includes the long-term trend (slope, m) for each parameter and the correlation coefficients (R^2) between wetland emissions and TWS in the legend.

Figure S20 Temporal Variations between optimized wetland emissions and temperature, precipitation, and terrestrial water storage (TWS) from 2003 to 2022. A correlation analysis between regional optimized wetland CH_4 emissions ($TgCH_4\ yr^{-1}$) from major wetland regions (panel a in Fig. 4a) for CH_4-only (red dashed) and Joint (red solid) scenarios, soil TWS (blue, cm), 2-m air temperature (skyblue, K), and precipitation (green, mm mon^{-1}). Each panel includes the long-term trend (slope, m) for each parameter and the correlation coefficients (R^2) between wetland emissions and TWS, temperature, and precipitation, in that order, as shown in the legend.

Figure S21 Mean and trends of optimized wetland CH_4 emissions using wetland priors with dynamic inundation and their correlation with terrestrial water storage from 2003 to 2022. (a) Spatial map of major wetland regions (green): 1 = Alaska, 2 = Hudson Bay, 3 = Southeast US, 4 = Amazon, 5 = South America, 6 = West Africa, 7 = Southeast Africa, 8 = Europe, 9 = West Siberia, 10 = East Siberia, 11 = North China, 12 = Southeast Asia. (b) Mean wetland emissions in $TgCH_4\ yr^{-1}$ from wetland regions (panel a) and total (rightmost) from inversions with (blue) and without (red) isotopic constraints. Uncertainty is estimated from the ensemble simulation (Supplementary Information). (c) Emission trends in $TgCH_4\ yr^{-2}$ by wetland regions (panel a) and the average (rightmost) for inversions with (blue) and without (red) isotopic constraints. Uncertainty from 1 standard deviation of the trends. (d) Coefficient of determination (R^2) between regional wetland CH_4 emissions and terrestrial water storage (cm) from major wetlands (panel a) and their R^2 mean (rightmost) for inversions with (blue) and without (red) isotopic constraints. Asterisks indicate significance (* < 0.1, ** < 0.05).

Figure S22 Temporal Variations between optimized wetland emissions with dynamic inundation and terrestrial water storage (TWS) from 2003 to 2022. A correlation analysis between regional optimized wetland CH_4 emissions using wetland priors with dynamic inundation (in $TgCH_4\ yr^{-1}$) and

TWS (in cm) from major wetland regions (Fig. 4a) for CH_4-only (red dashed) and Joint scenario (red solid). Each panel includes the long-term trend (slope, m) for each parameter and the correlation coefficients (R^2) between wetland emissions and TWS in the legend.

Figure S23 Differences in correlations between terrestrial water storage and optimized wetland CH_4 emissions in Joint and CH_4-only scenarios. Differences in correlation coefficients (Δr) between terrestrial water storage (TWS) and regional optimized wetland CH_4 emissions from the Joint scenario (r(Joint,TWS)) and the CH_4-only scenario (r(CH_4-only,TWS)) are shown using wetland priors derived from (a) static inundation and (b) dynamic inundation across the 12 major wetland regions (Fig. 4a). Star symbols indicate statistically significant differences in Δr ($p < 0.05$).

Figure S24 Global annual emissions of sources using climatological priors. (a-d) Long-term changes in CH_4 emissions from (a) total, (b) microbial, (c) fossil, and (d) pyrogenic sources for prior (Green), Joint+climatological prior (Blue), and CH_4-only +climatological priors (Red) scenarios during 2006-2021.

Figure S25 Global observed and simulated long-term trends in atmospheric CH_4 and $\delta^{13}C$-CH_4. Observed and simulated averaged (a) CH_4 in ppb and (b) $\delta^{13}C$-CH_4 in ‰ in 2000-2021. For each sub-plots in (a)-(b), (top) time series of averaged values and (bottom) difference between simulated and observed values for observed (black) and posterior from Joint (blue), Joint+climatological priors (skyblue), and CH_4-only+climatological priors (magenta) scenarios.

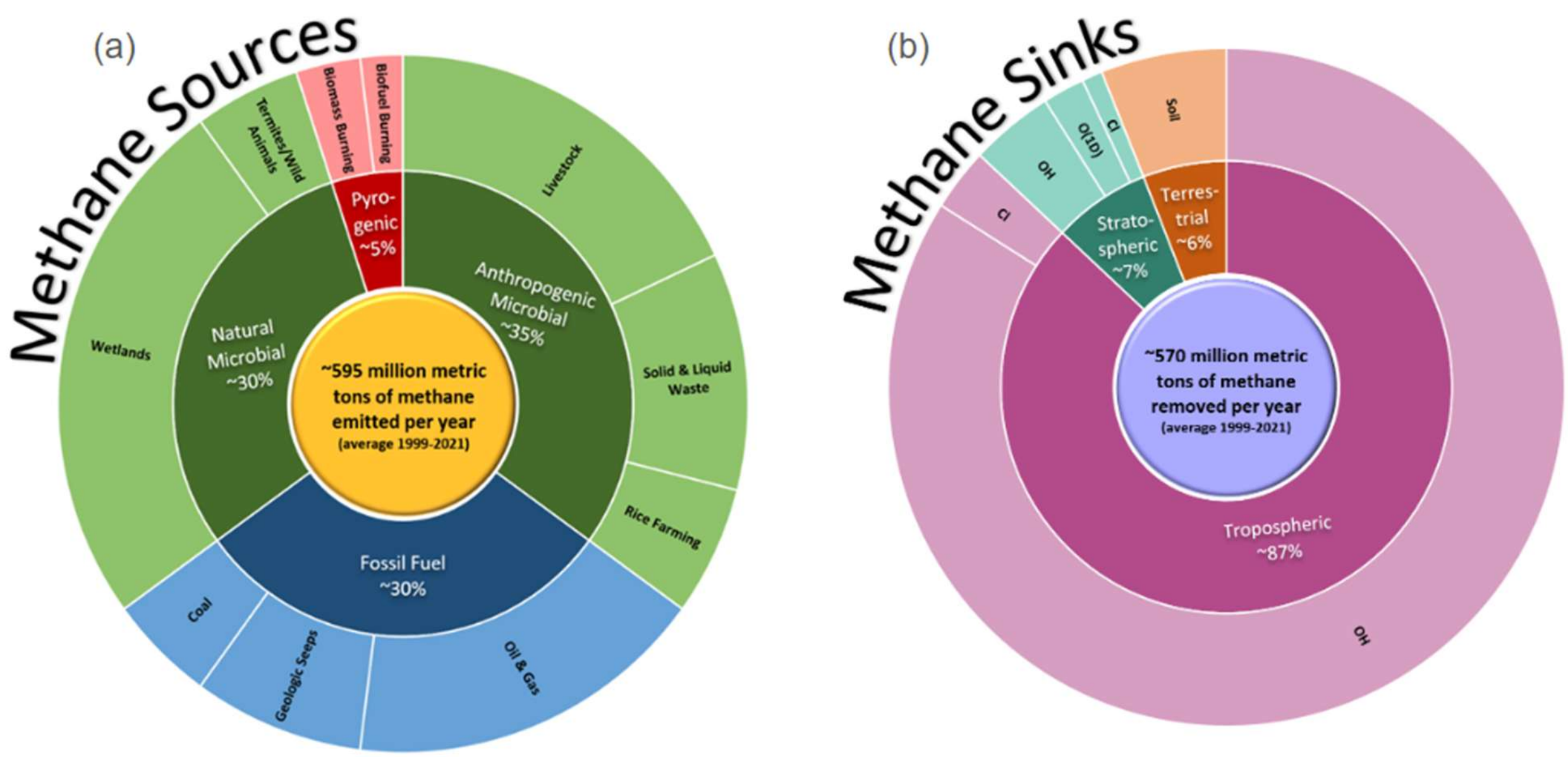

Figure S1 Pie chart of global source and sink estimation over 2000-2022. (a) Pie chart of global mean CH_4 emissions from anthropogenic and natural microbial, fossil, and pyrogenic source sectors based on the results from the "Joint" inversion presented in this study. (b) Pie chart of global mean CH_4 oxidation from tropospheric, stratospheric, and terrestrial sink sectors. The percentage of source and sink sectors is based on our prior setup (Table S3-4).

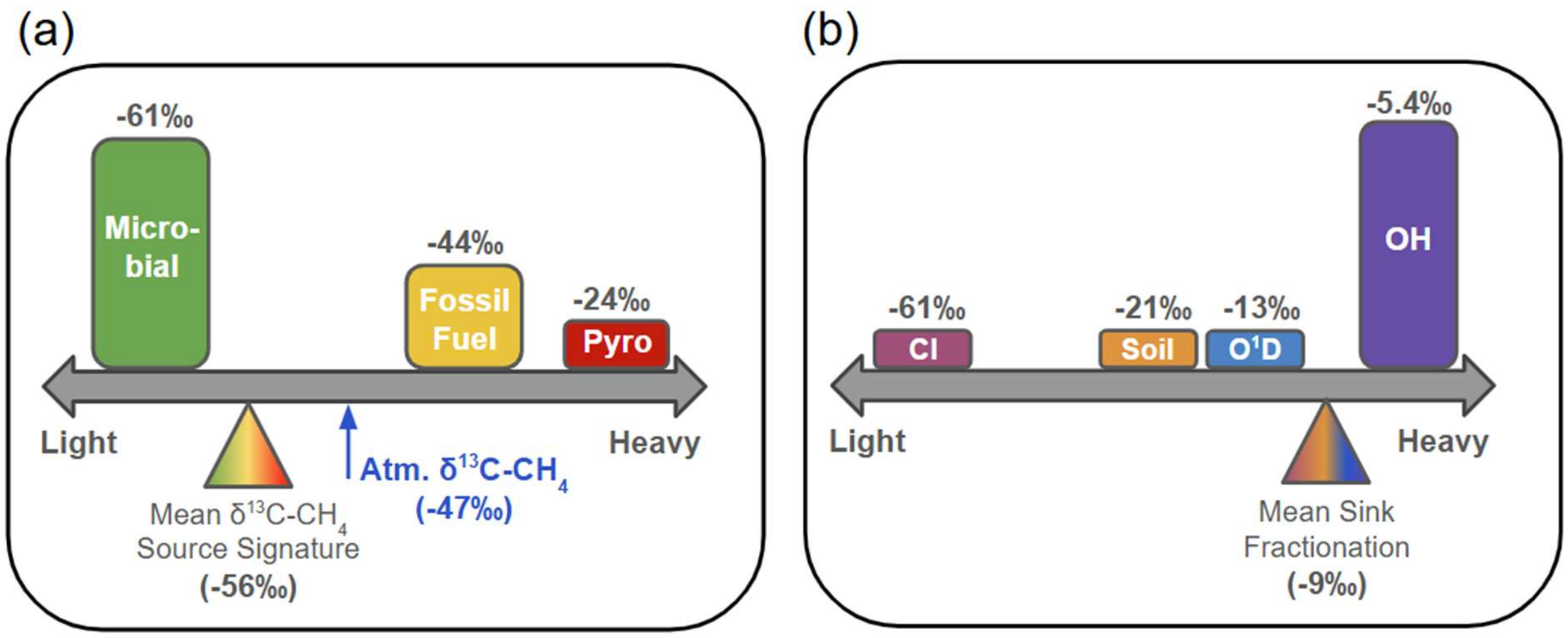

Figure S2 Global mean isotopic signature of sources and fractionation of sinks. (a) Global mean flux-weighted CH_4 isotopic signature ($\delta^{13}C$-CH_4) of microbial (green), fossil (yellow), and pyrogenic (red) sources. Approximate values of global mean source signatures are shown (Table S2), and the size of each bar reflects the relative flux from each source category. The flux-weighted source signature across all sources is estimated to be ~–56‰, and the difference between this value and the atmospheric signal (~–47‰; "Atm. $\delta^{13}C$-CH_4") is attributed to process-weighted sink fractionation (~9‰), as illustrated in panel (b). (b) The global mean process-weighted isotopic signature of atmospheric chemical sinks from OH (dark blue), Cl (dark magenta), and O^1D (blue), as well as soil sinks (orange). The size of each bar approximates the relative sink strength, although the OH sink is much larger than the other sinks (Fig. S1).

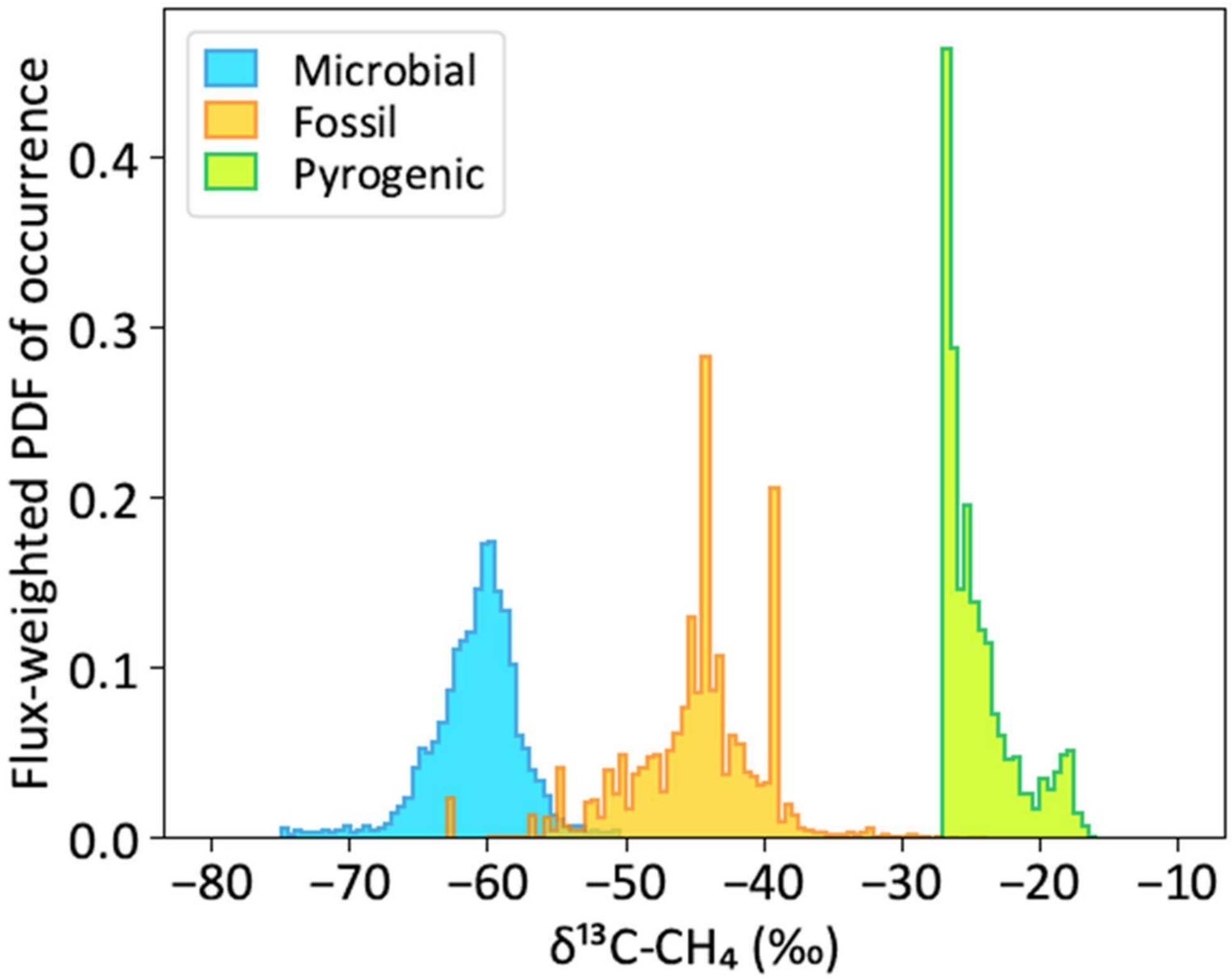

Figure S3 Flux-weighted probability distribution of source signatures. Histogram of flux-weighted probability distribution function of δ^{13}C-CH_4 from microbial (blue), fossil (yellow), and pyrogenic (green) sources.

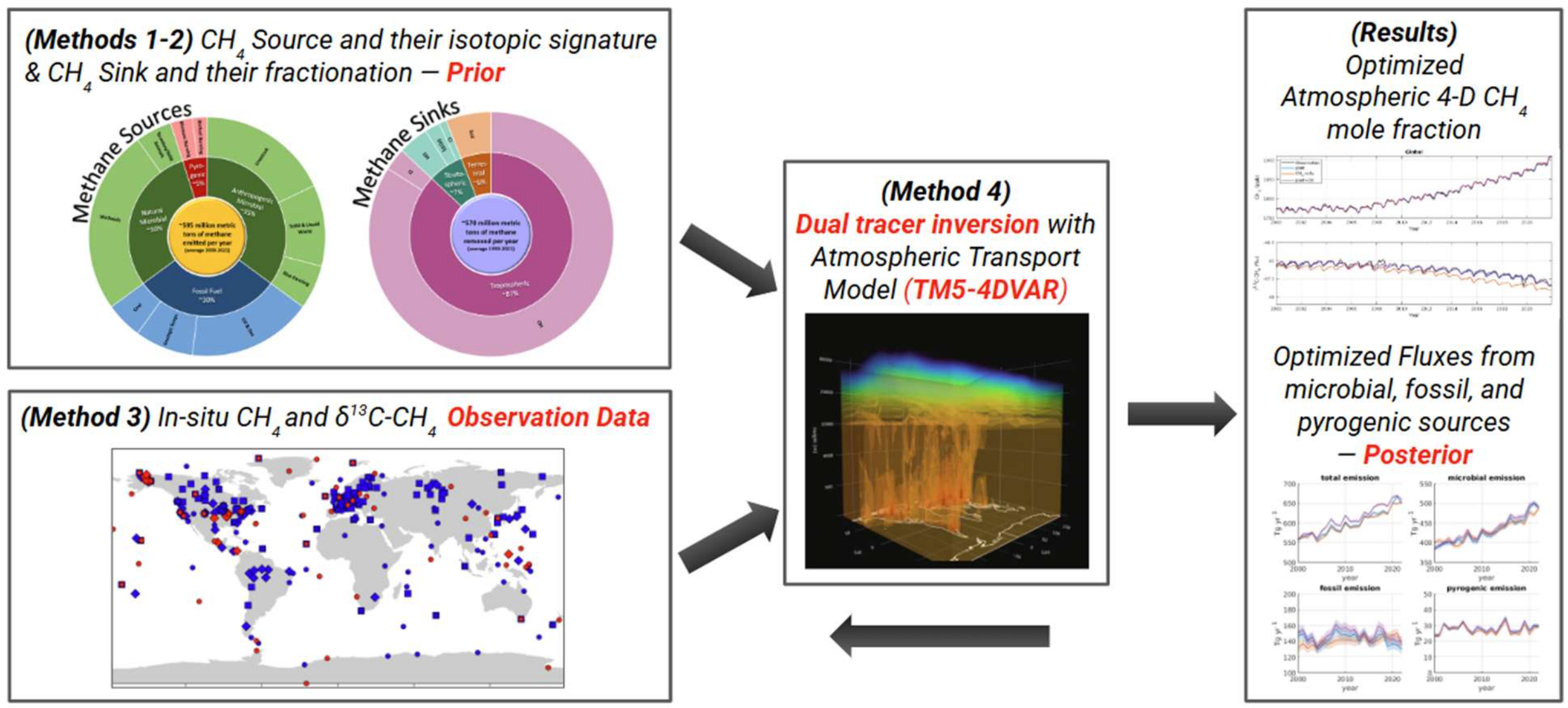

Figure S4. Schematic diagram of CarbonTracker-CH_4 atmospheric inversion system. The goal of the CarbonTracker-CH_4 is to produce quantitative estimates of methane (CH_4) emissions at the Earth's surface that are consistent with observed patterns of CH_4 and its stable carbon isotope ratios ($\delta^{13}C$-CH_4) in the atmosphere. (Methods 1) Prior information on sector-specific CH_4 source fluxes and their signatures and isotopic fractionation by chemical and soil sinks and their sink size is prescribed. (Method 2) In-situ observations of atmospheric CH_4 and $\delta^{13}C$-CH_4 provide the observational constraints. (Method 3) These priors and observations are combined within a dual-tracer inversion system using the TM5 atmospheric transport model (TM5 4DVar), which simulates transport driven by meteorology and optimizes prior surface emissions from microbial, fossil, and pyrogenic sources. Differences between modeled and observed atmospheric fields are attributed to errors in prior emissions and are minimized through the inversion to produce posterior flux estimates. The optimized outputs include global three-dimensional atmospheric CH_4 mole-fraction fields and monthly posterior flux estimates for microbial, fossil, and pyrogenic sources.

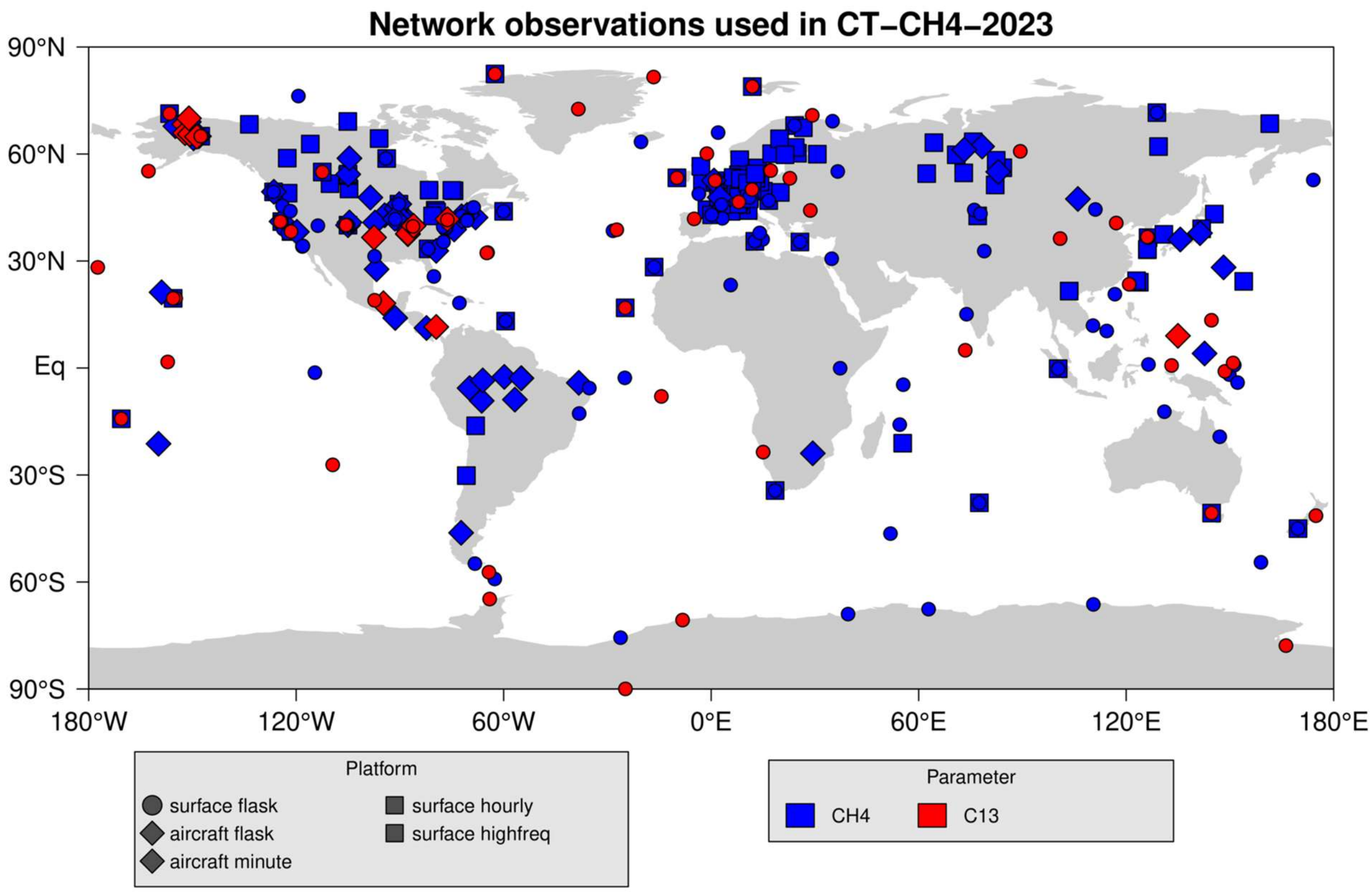

Figure S5. Global distribution of in-situ CH_4 and $\delta^{13}C$-CH_4 used in this study. Mean locations of measurements for CH_4 (blue) and $\delta^{13}C$-CH_4 (red) from surface flask samples (circle), surface hourly and continuous high-frequency data from tower (square), and flask and continuous high-frequency data from aircraft platforms (diamond). More information on the participating laboratories and their datasets, as well as site-specific model–data mismatches and the selection of assimilation and validation sites for atmospheric CH_4 and $\delta^{13}C$-CH_4 data, is provided in Table S6.

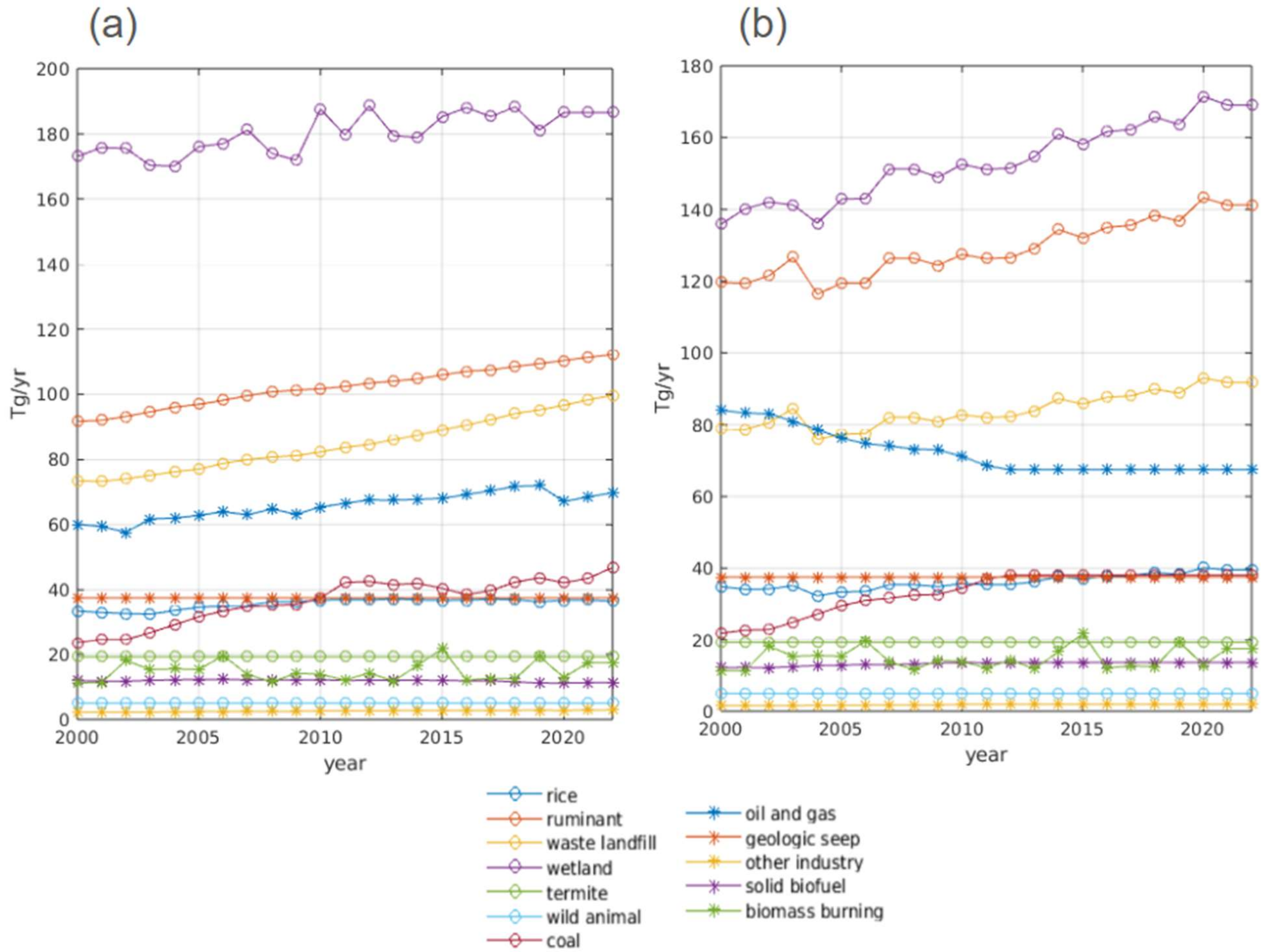

Figure S6 Global annual emissions of sources. Long-term annual emissions (in $TgCH_4$ yr^{-1}) of total microbial, fossil, and pyrogenic sources from each source sector (a) before and (b) after the global mass balance of atmospheric CH_4 and $\delta^{13}C$-CH_4 (Supplementary Information and Table S3).

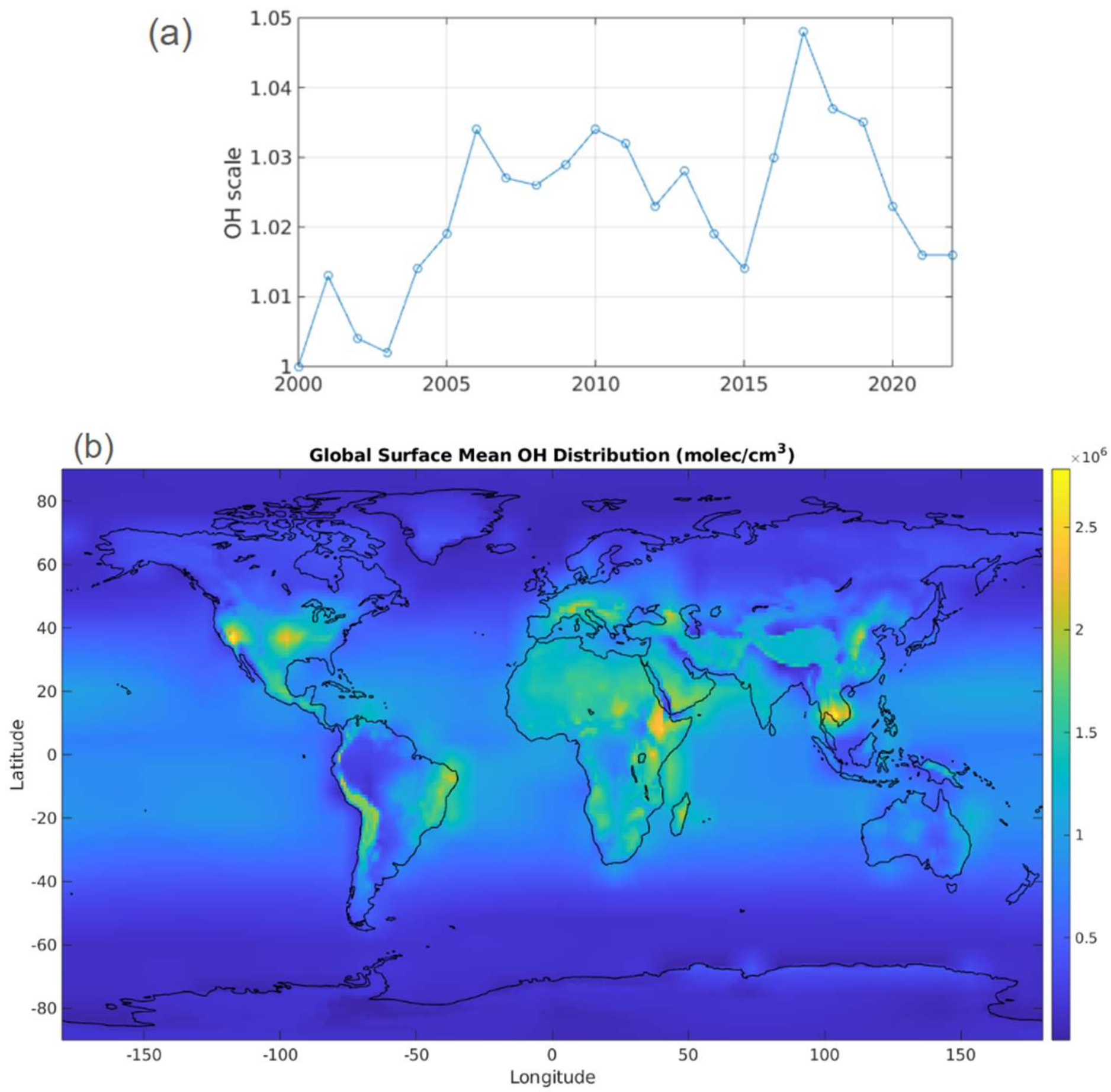

Figure S7 Annual scaling factor and distribution of surface OH. (a) Annual scaling factor of tropospheric OH for the Joint+OH scenario during 2000-2022. The estimated scaling factor is based on an atmospheric chemistry model, LMDz-INCA[3,12,13]. (b) Global distribution of OH at the surface level from Spivakovsky et al. (2000)[14] after scaling by 0.9 to match the declining atmospheric abundance of methyl chloroform in the early 2000s[5,15].

(a)

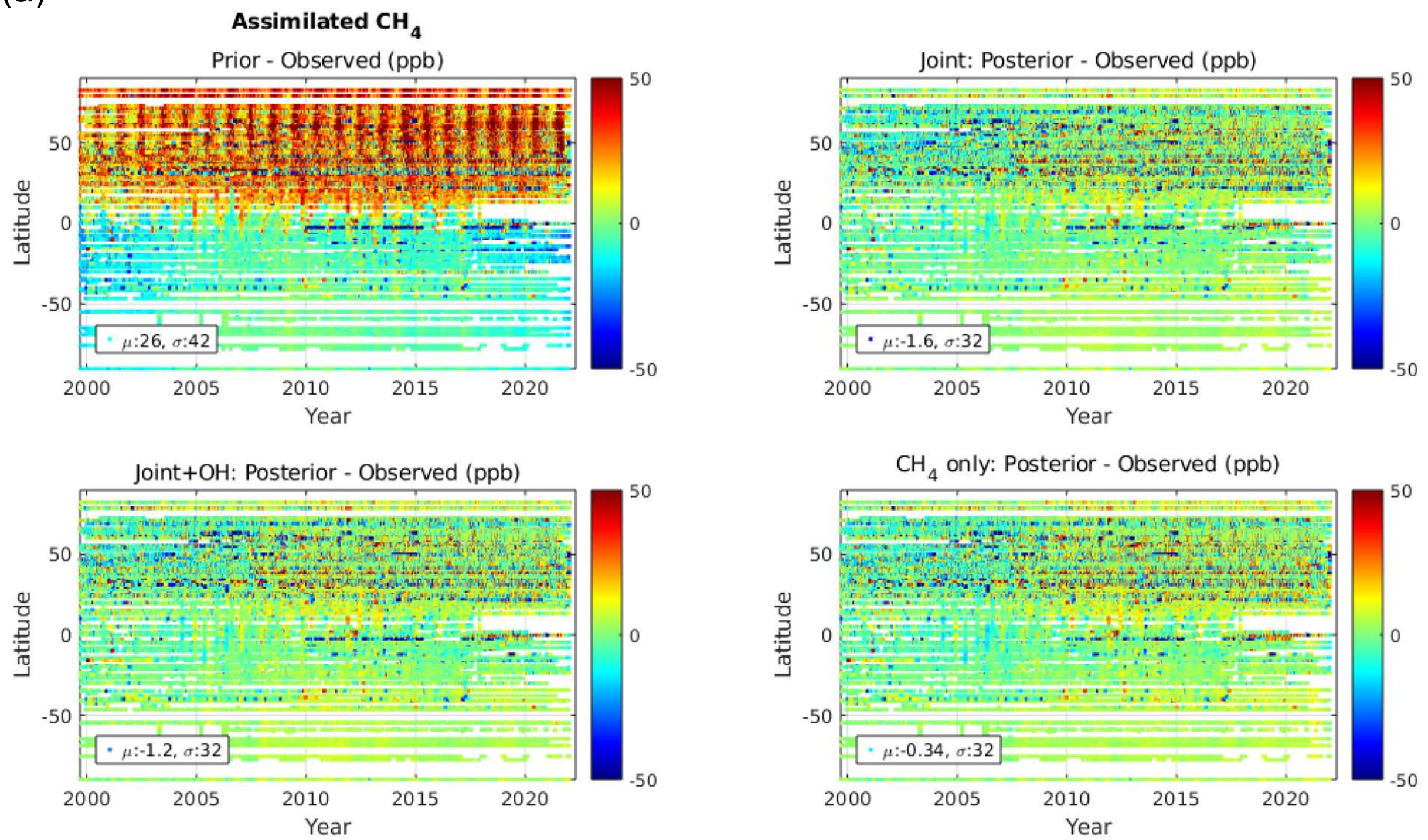

(b)

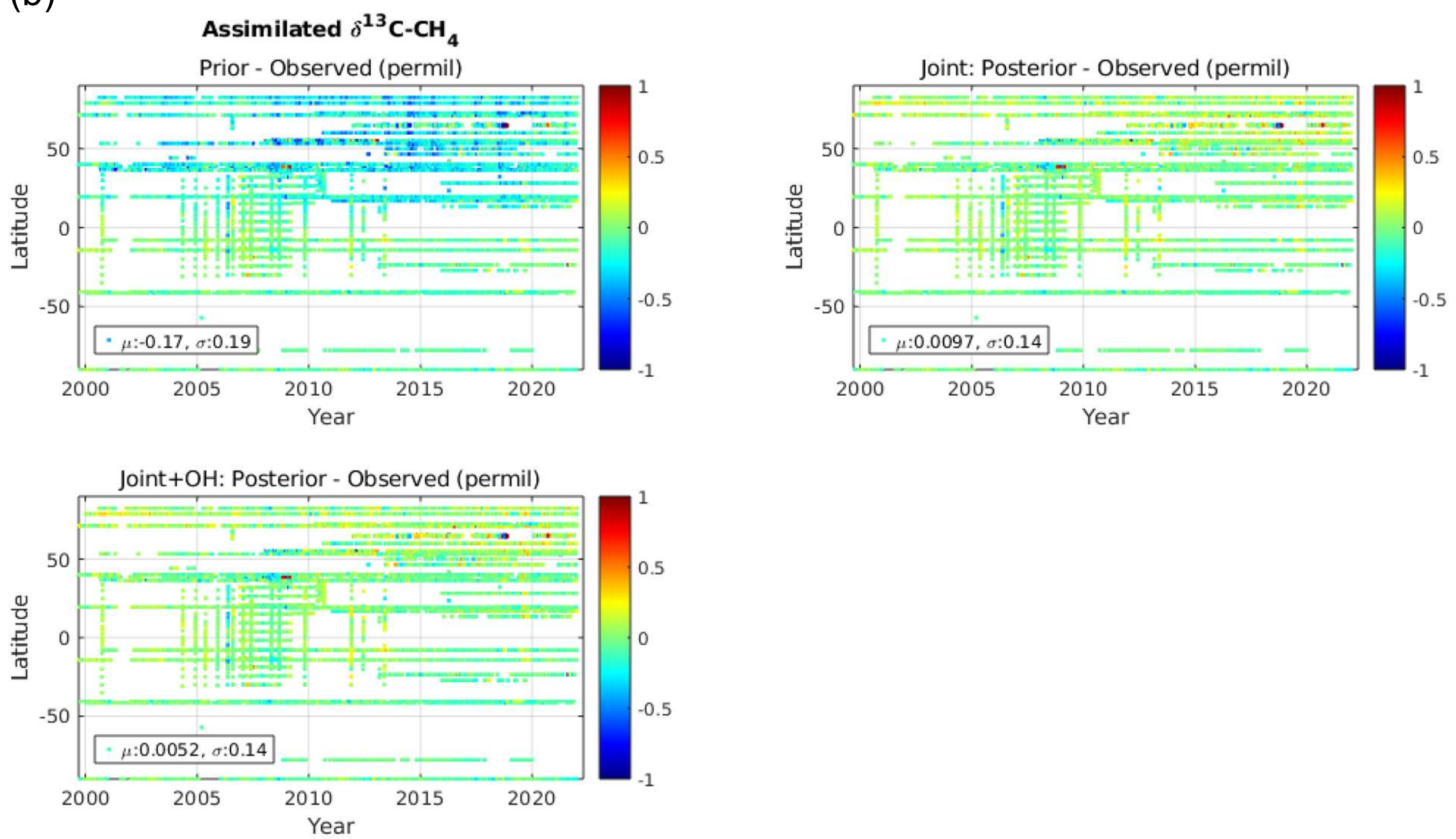

Figure S8 Summary of residuals of assimilated CH_4 and δ^{13}C-CH_4. Residuals for (a) CH_4 and (b) δ^{13}C-CH_4 of assimilated data that are used to optimize CH_4 flux during the atmospheric inversion. Simulated minus observed values (CH_4 in nmol mol^{-1} (ppb), δ^{13}C-CH_4 in ‰) are as a function of time and latitude for prior (top left) and posterior estimation from Joint (top right), Joint+OH (bottom left), and CH_4-only (bottom right scenarios). Each dot represents the time and location of an observation, and the legend shows mean bias (μ) and standard deviation of bias (σ).

(a)

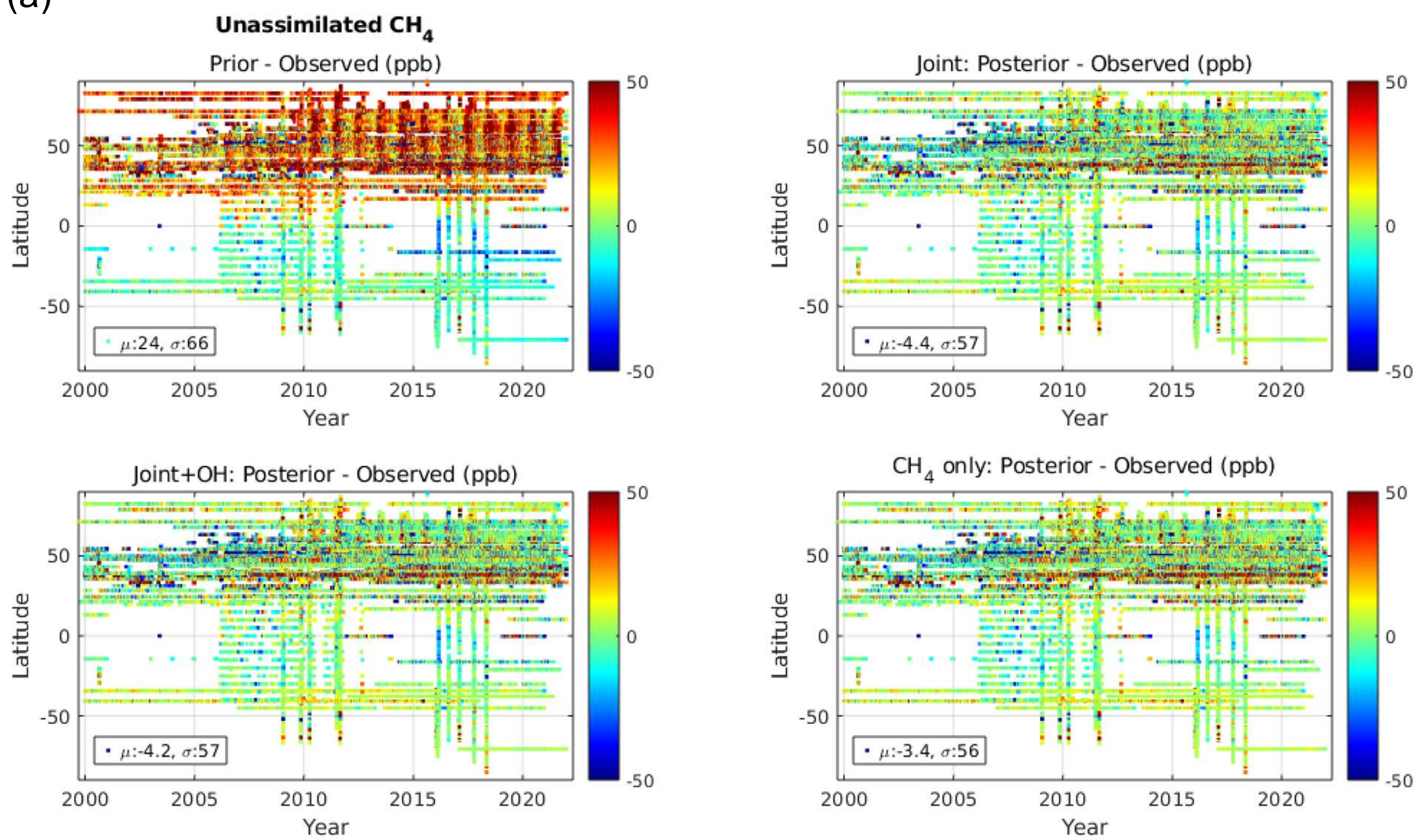

(b)

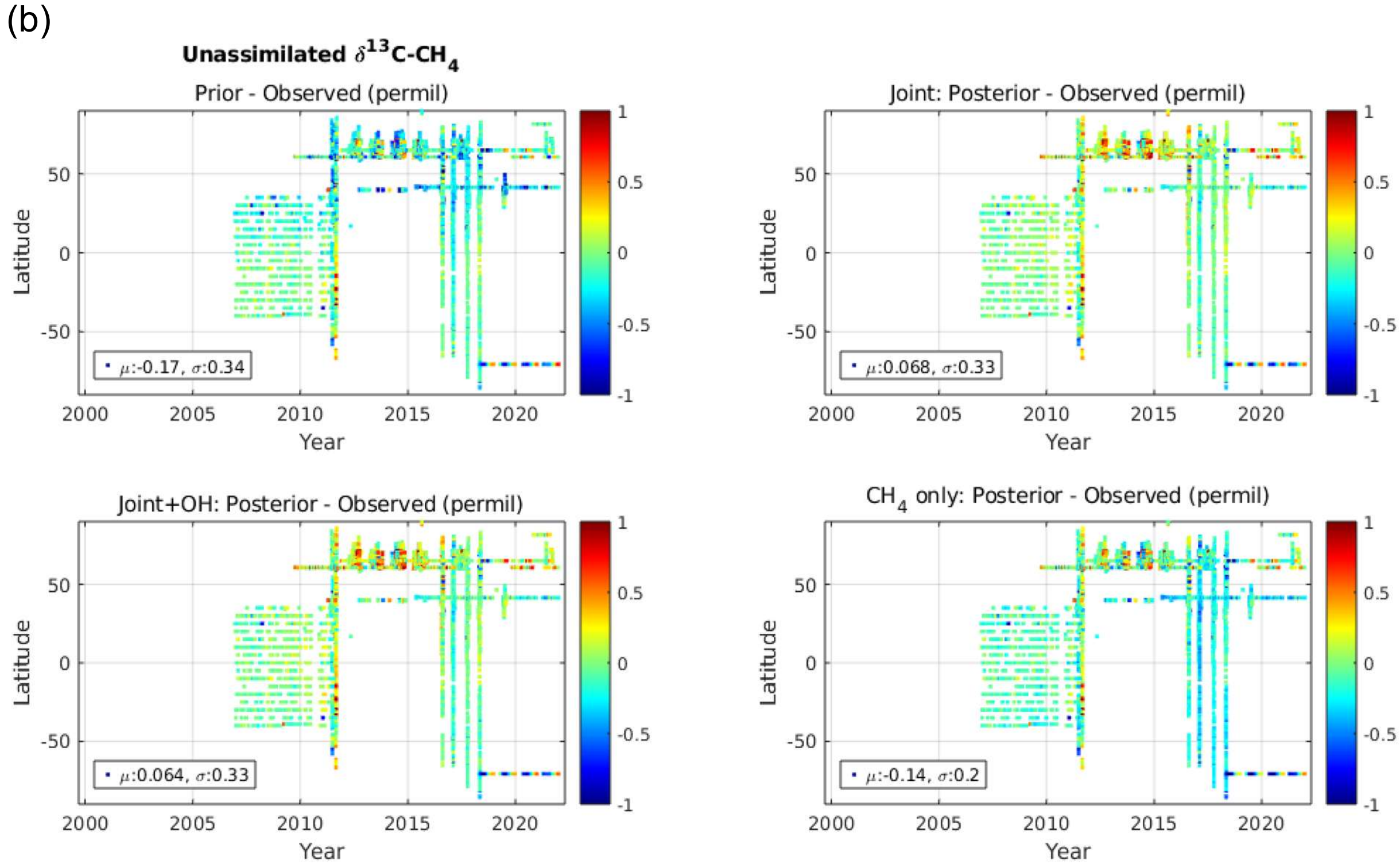

Figure S9 Summary of residuals of unassimilated CH_4 and $\delta^{13}C$-CH_4. Residuals for (a) CH_4 and (b) $\delta^{13}C$-CH_4 of unassimilated data that are not used to optimize CH_4 flux during the atmospheric inversion for model validation. Simulated minus observed values (CH_4 in nmol mol^{-1} (ppb), $\delta^{13}C$-CH_4 in ‰) are as a function of time and latitude for prior (top left) and posterior estimation from Joint (top right), Joint+OH (bottom left), and CH_4-only (bottom right scenarios). Each dot represents the time and location of an observation, and the legend shows mean bias (μ) and standard deviation of bias (σ).

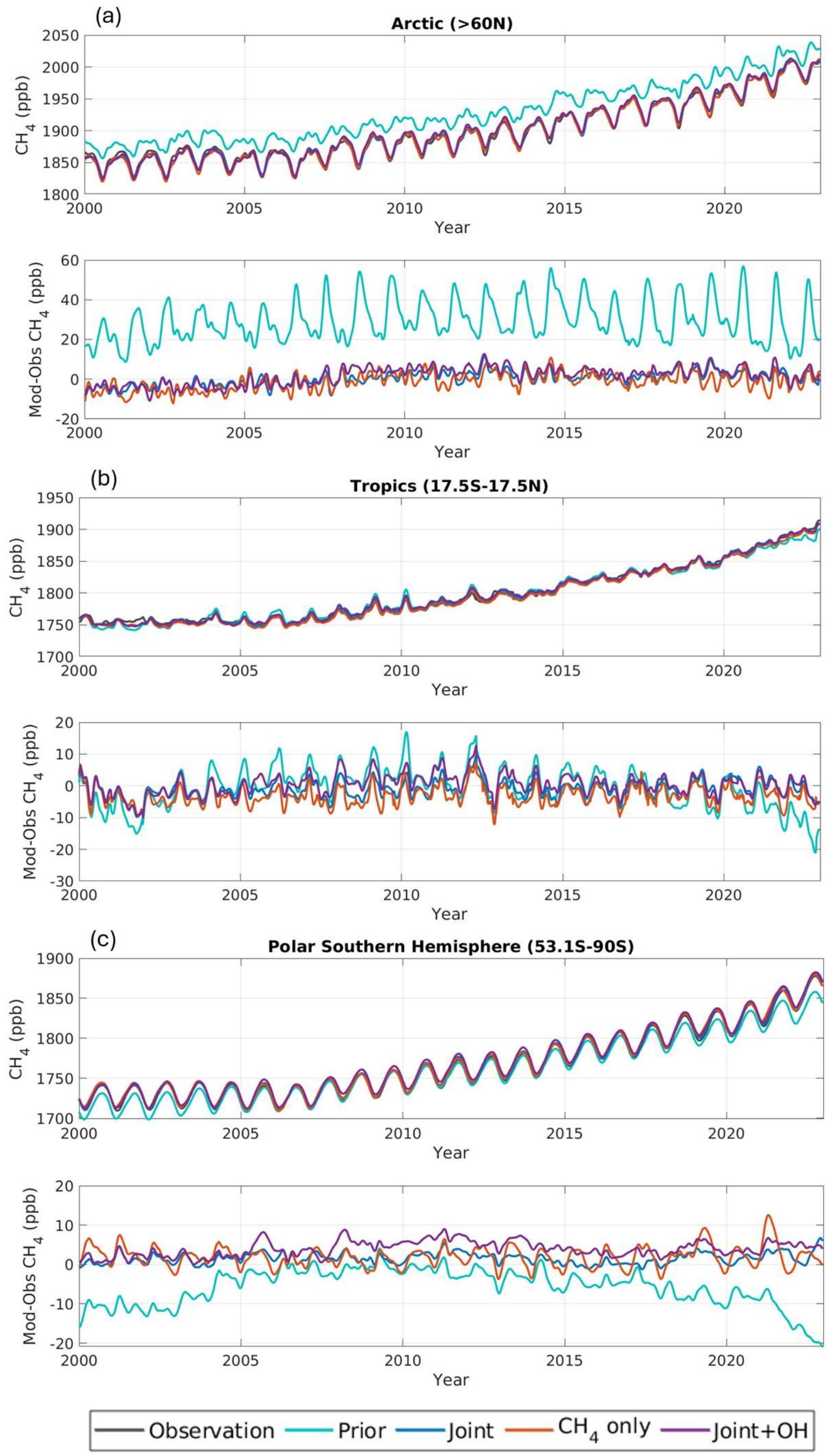

Figure S10 Regional observed and simulated long-term trends in atmospheric CH_4. Observed and simulated averaged CH_4 in ppb for Arctic (a), tropics (b), and polar southern hemisphere (c) regions in 2000-2022. For each sub-plot in (a)-(c), (top) time series of averaged values and (bottom) difference between simulated and observed values for observed (black), prior (dark green), and posterior from Joint (blue), CH_4-only (red), and Joint+OH (purple) scenarios.

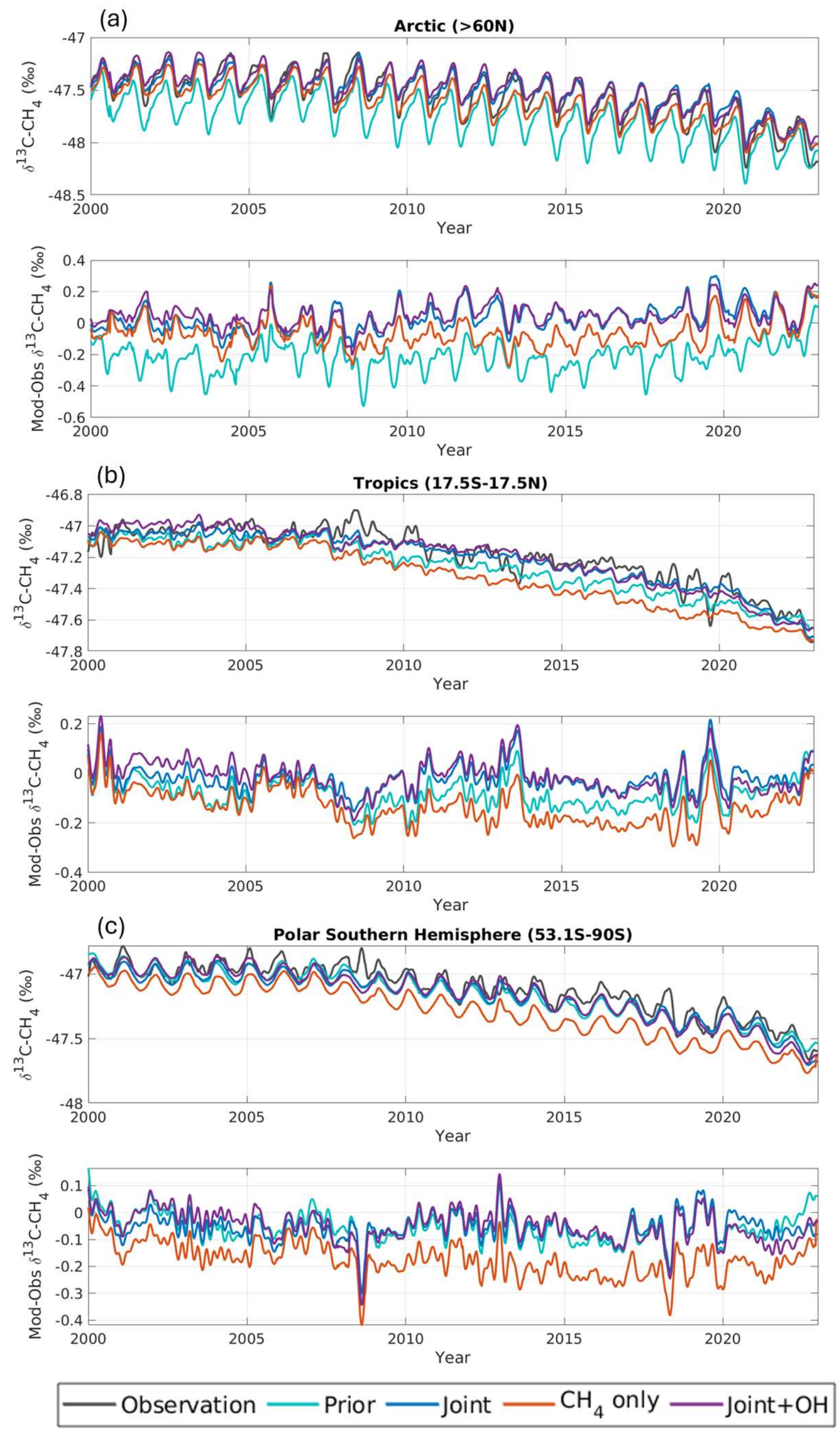

Figure S11 Regional observed and simulated long-term trends in atmospheric δ^{13}C-CH_4. Observed and simulated averaged CH_4 in ppb for Arctic (a), tropics (b), and polar southern hemisphere (c) regions in 2000-2022. For each sub-plot in (a)-(c), (top) time series of averaged values and (bottom) difference between simulated and observed values for observed (black), prior (dark green), and posterior from Joint (blue), CH_4-only (red), and Joint+OH (purple) scenarios.

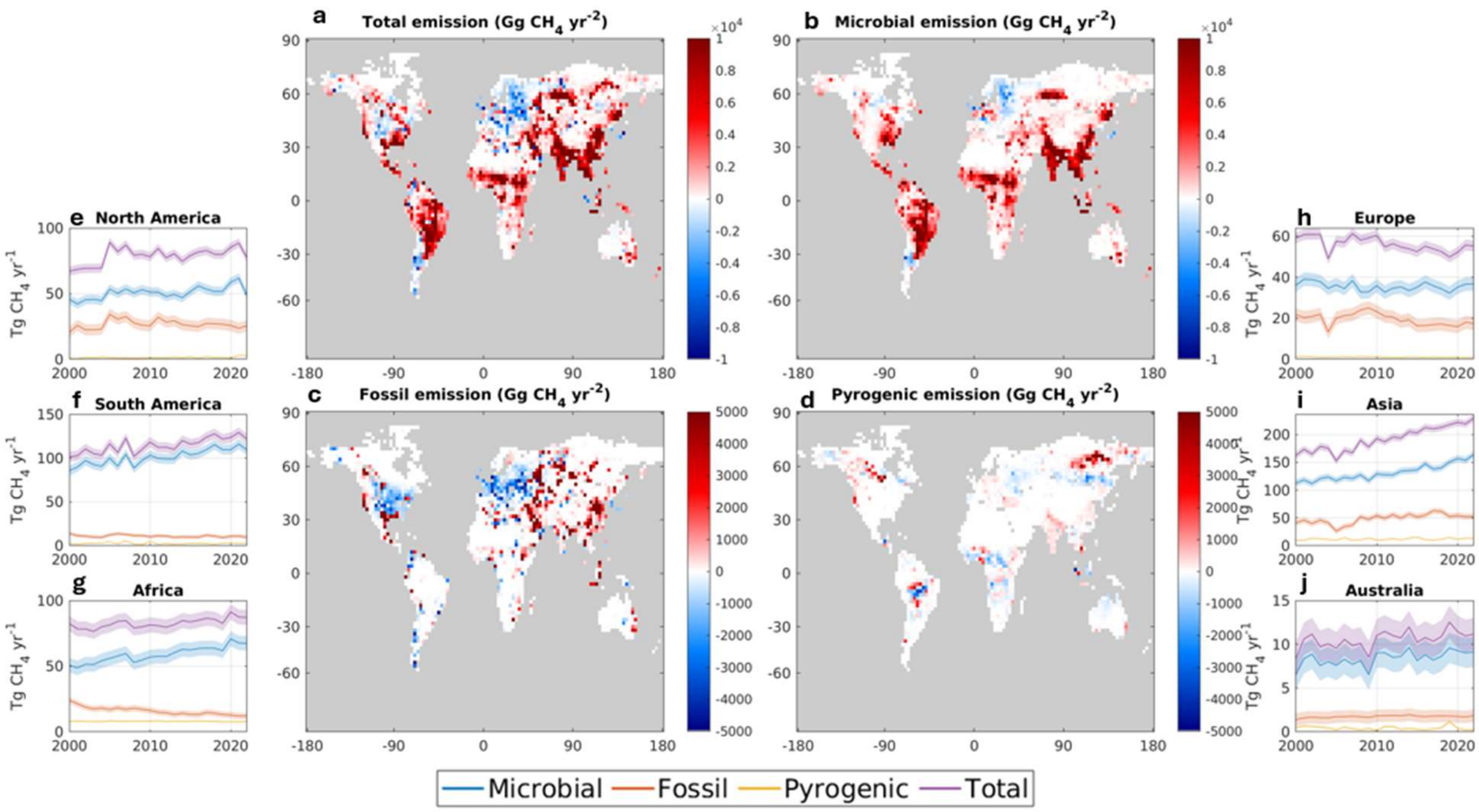

Figure S12 Global and regional trends in emissions during 2000-2022 for Joint+OH Scenario. (a-d) Spatial distribution of trends in long-term CH_4 emissions in $GgCH_4$ yr^{-2} from (a) total, (b) microbial, (c) fossil, and (d) pyrogenic sources during 2000-2022 for Joint+OH scenario. (e-j) Long-term trends in regional CH_4 emissions from (e) North America, (f) South America, (g) Africa, (h) Europe, (i) Asia, and (j) Australia from microbial (Blue), fossil (red), pyrogenic (yellow), and total (purple) sources. Uncertainty is estimated from the ensemble simulation (Supplementary Information).

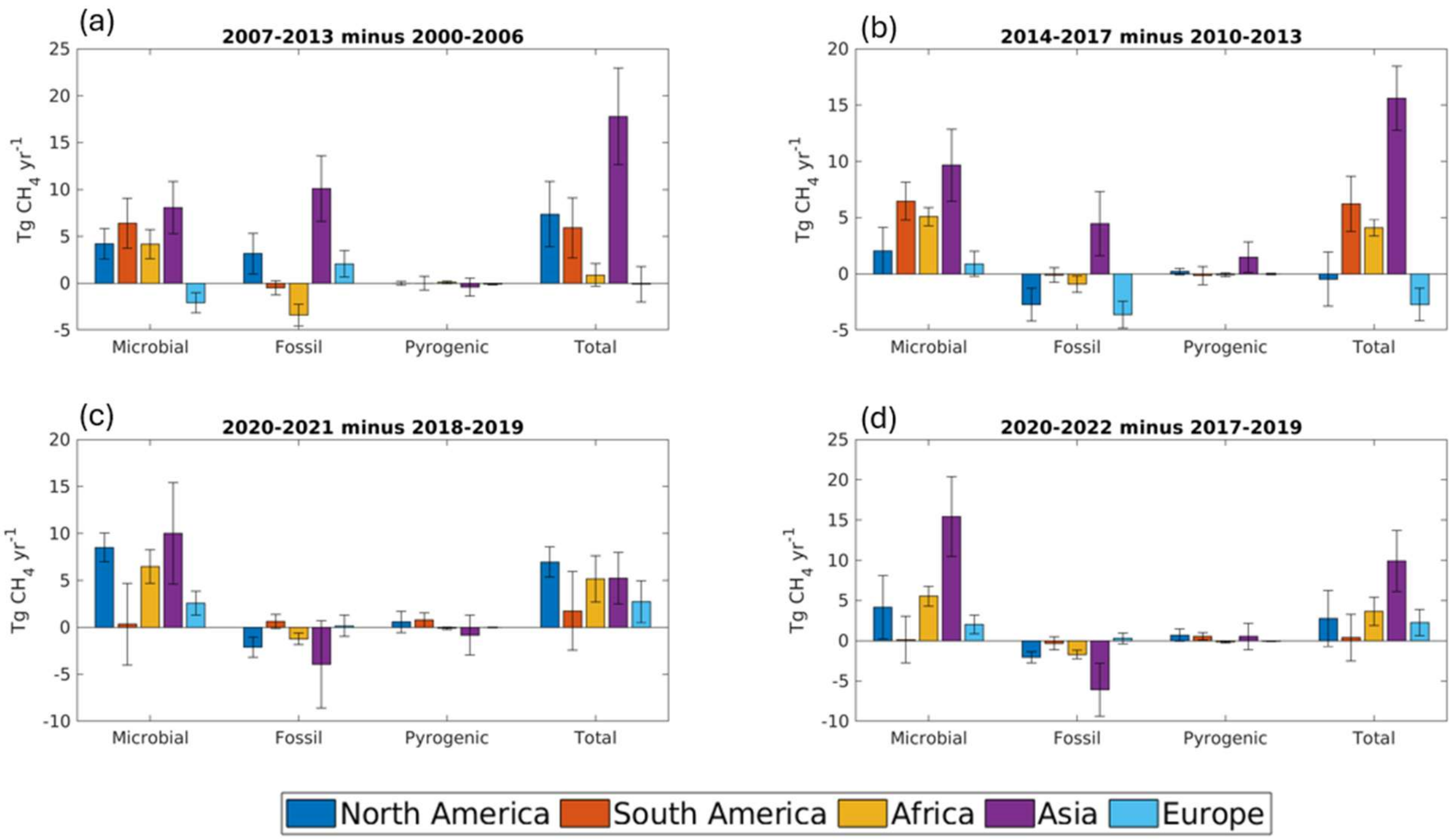

Figure S13 Differences in regional CH_4 emissions for the Joint+OH Scenario. Differences in regional CH_4 emissions between (a) 2000-2006 and 2007-2013, (b) 2010-2013 and 2014-2017, (c) 2018-2019 and 2020-2021, and (d) 2017-2019 and 2020-2022 in TgCH_4 yr^{-1} from North America (blue), South America (red), Africa (yellow), Asia (purple), and Europe (skyblue) continents. Uncertainty is estimated from the ensemble simulation (Supplementary Information).

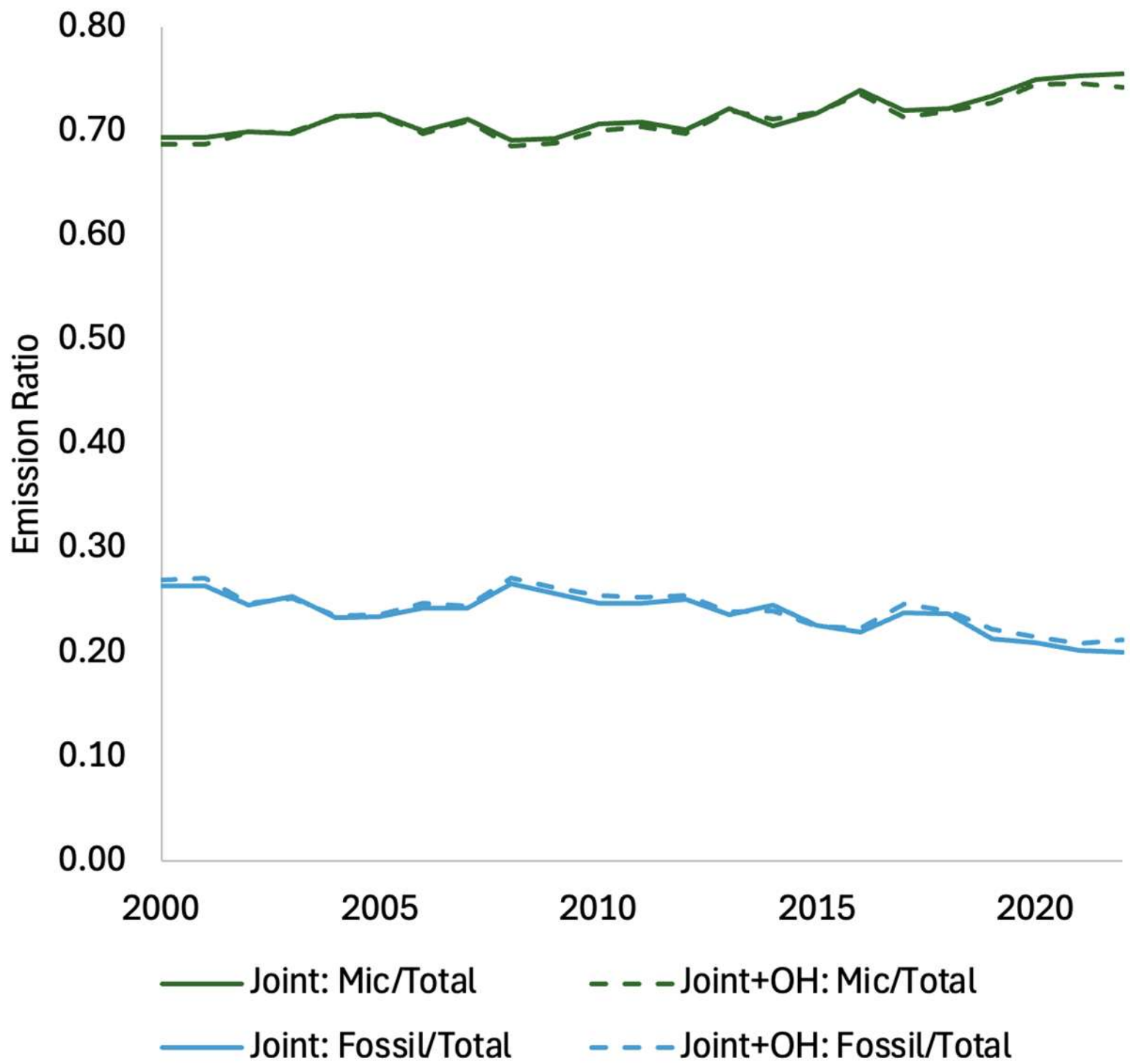

Figure S14 Emission ratios for Joint and Joint+OH scenarios. The ratio of fossil emissions to total emissions (light blue) and microbial emissions to total emissions (green) in the Joint (solid lines) and Joint+OH scenarios (dashed lines). Changes in emission ratios between the two scenarios never exceeds 1% of total emissions for any year.

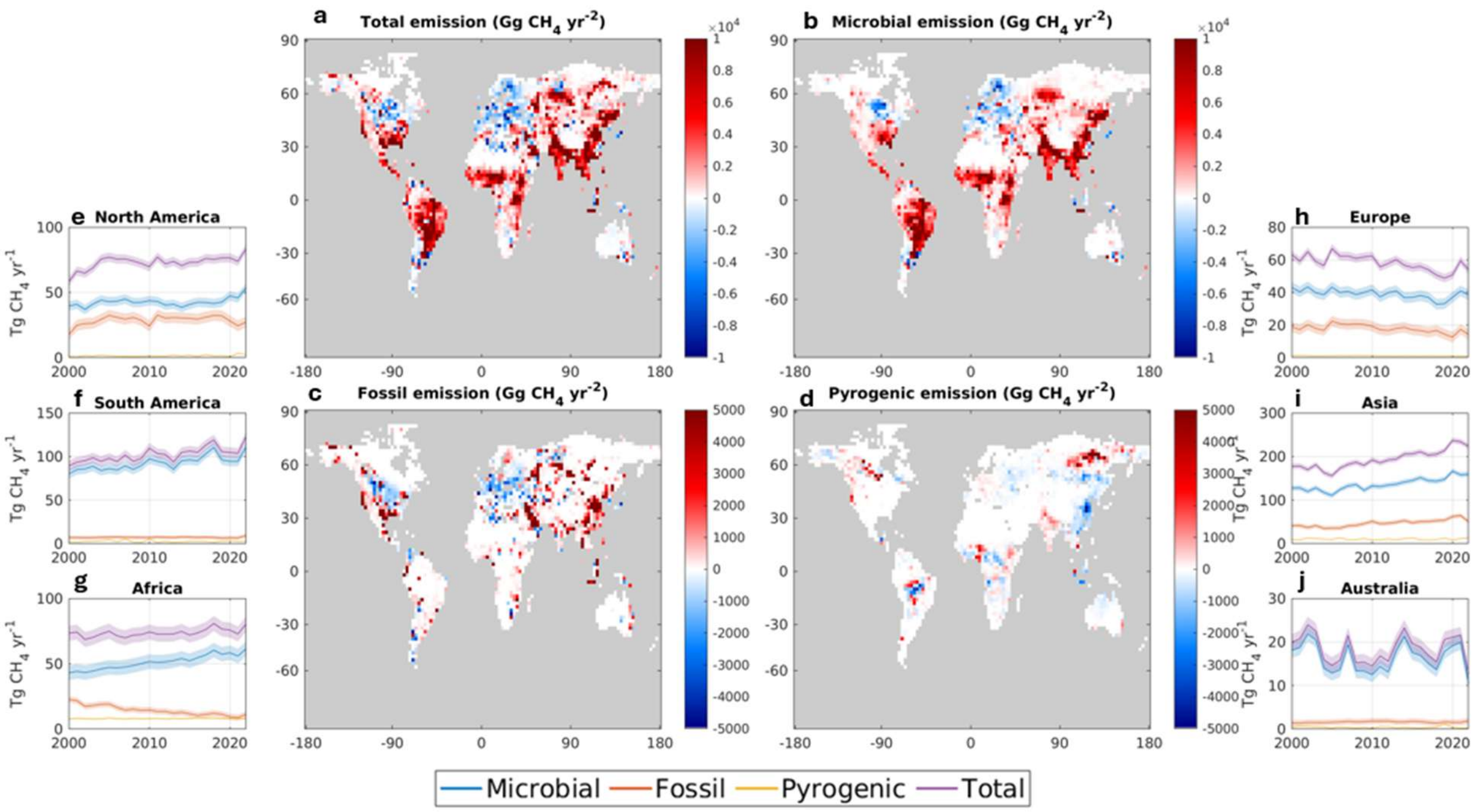

Figure S15 Global and regional trends in emissions during 2000-2022 for CH_4-only Scenario. (a-d) Spatial distribution of trends in long-term CH_4 emissions in $GgCH_4\ yr^{-2}$ from (a) total, (b) microbial, (c) fossil, and (d) pyrogenic sources during 2000-2022 for CH_4-only scenario. (e-k) Long-term trends in regional CH_4 emissions from (e) North America, (f) South America, (g) Africa, (h) Europe, (i) Asia, and (j) Australia from microbial (Blue), fossil (red), pyrogenic (yellow), and total (purple) sources. Uncertainty is estimated from the ensemble simulation (Supplementary Information).

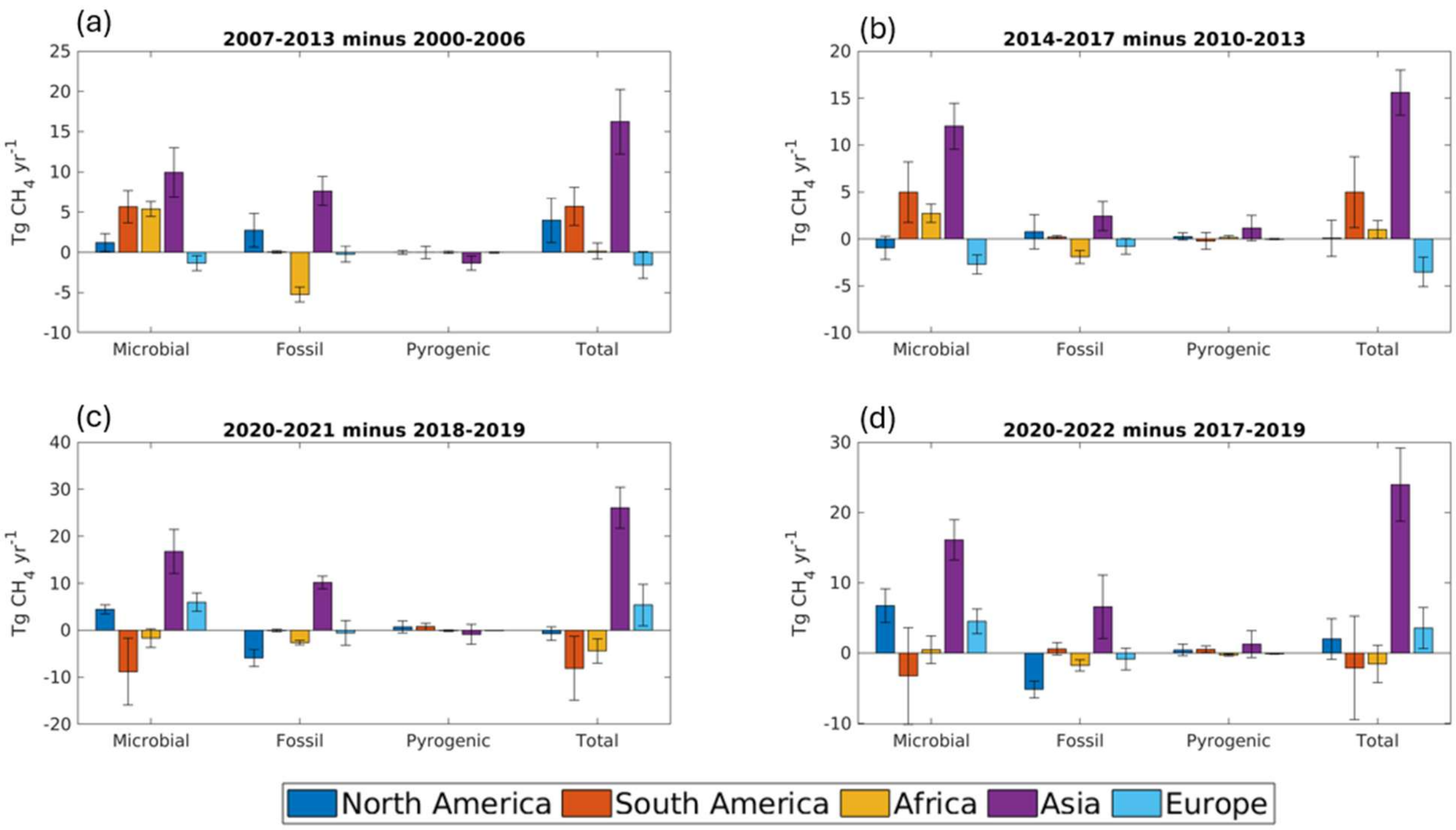

Figure S16 Differences in regional CH_4 emissions for the CH_4-only Scenario. Differences in regional CH_4 emissions between (a) 2000-2006 and 2007-2013, (b) 2010-2013 and 2014-2017, (c) 2018-2019 and 2020-2021, and (d) 2017-2019 and 2020-2022 in $TgCH_4$ yr^{-1} from North America (blue), South America (red), Africa (yellow), Asia (purple), and Europe (skyblue) continents. Uncertainty is estimated from the ensemble simulation (Supplementary Information).

(a)

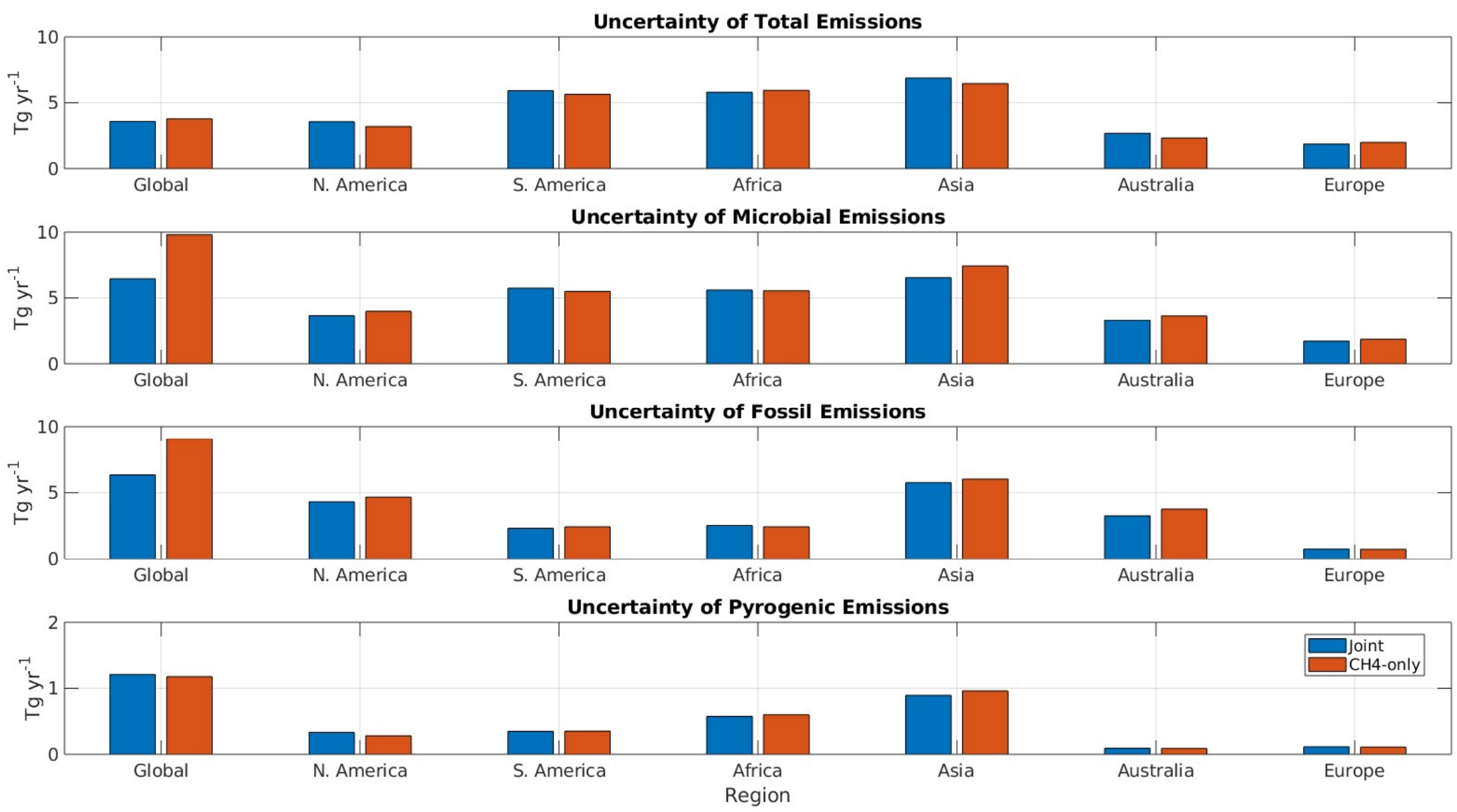

(b)

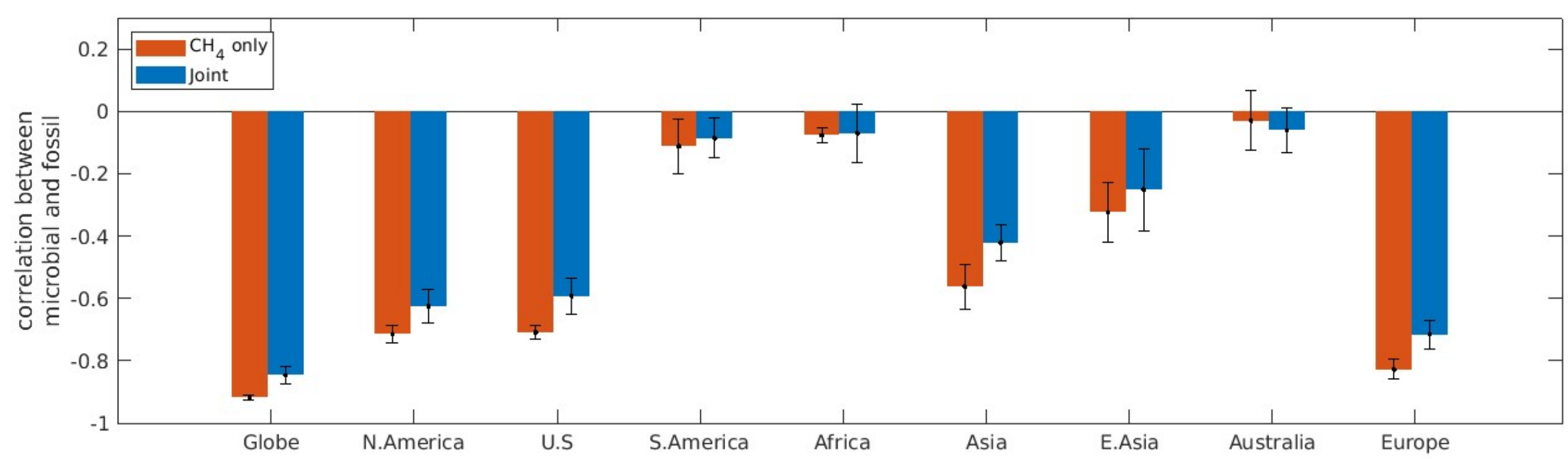

Figure S17 Spatial uncertainty and correlation between microbial and fossil emissions. (a) Global and regional uncertainty of total, microbial, fossil, and pyrogenic emissions (in $TgCH_4\ yr^{-1}$) from ensemble simulations (Supplementary Information). (b) Posterior correlation between global annual microbial and fossil methane emissions for Scenarios Joint (Blue) and CH_4-only (Red) for global and regional scales (North America, US, South America, Africa, Asia, East Asia, Australia, and Europe).

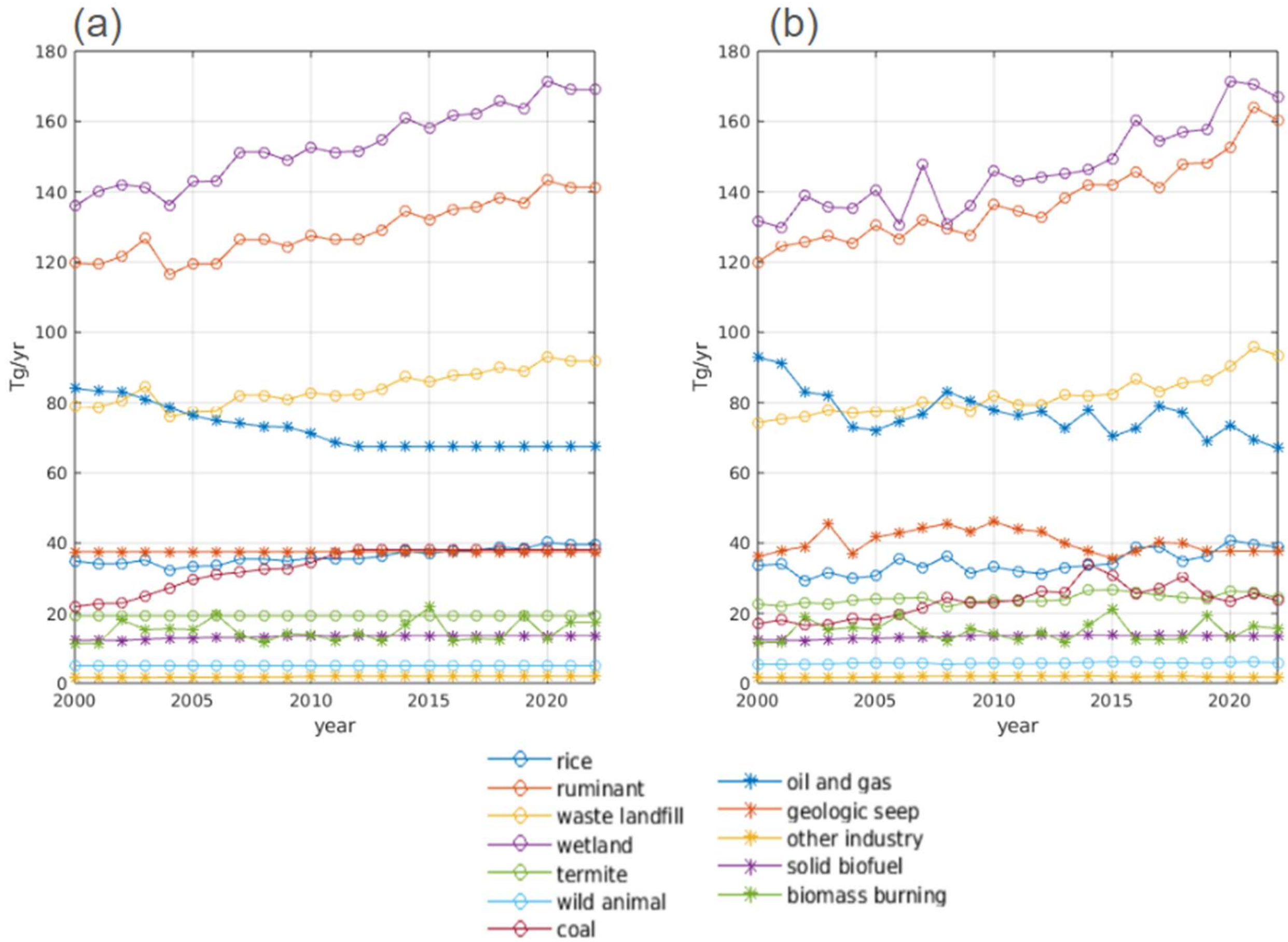

Figure S18 Global annual emissions of sources from prior and posterior simulation for the Joint scenario. Long-term annual emissions (in $TgCH_4$ yr^{-1}) of microbial, fossil, and pyrogenic sources from each source sector from (a) mass-balanced priors (same as Fig. S6b) and (b) the inversion posterior.

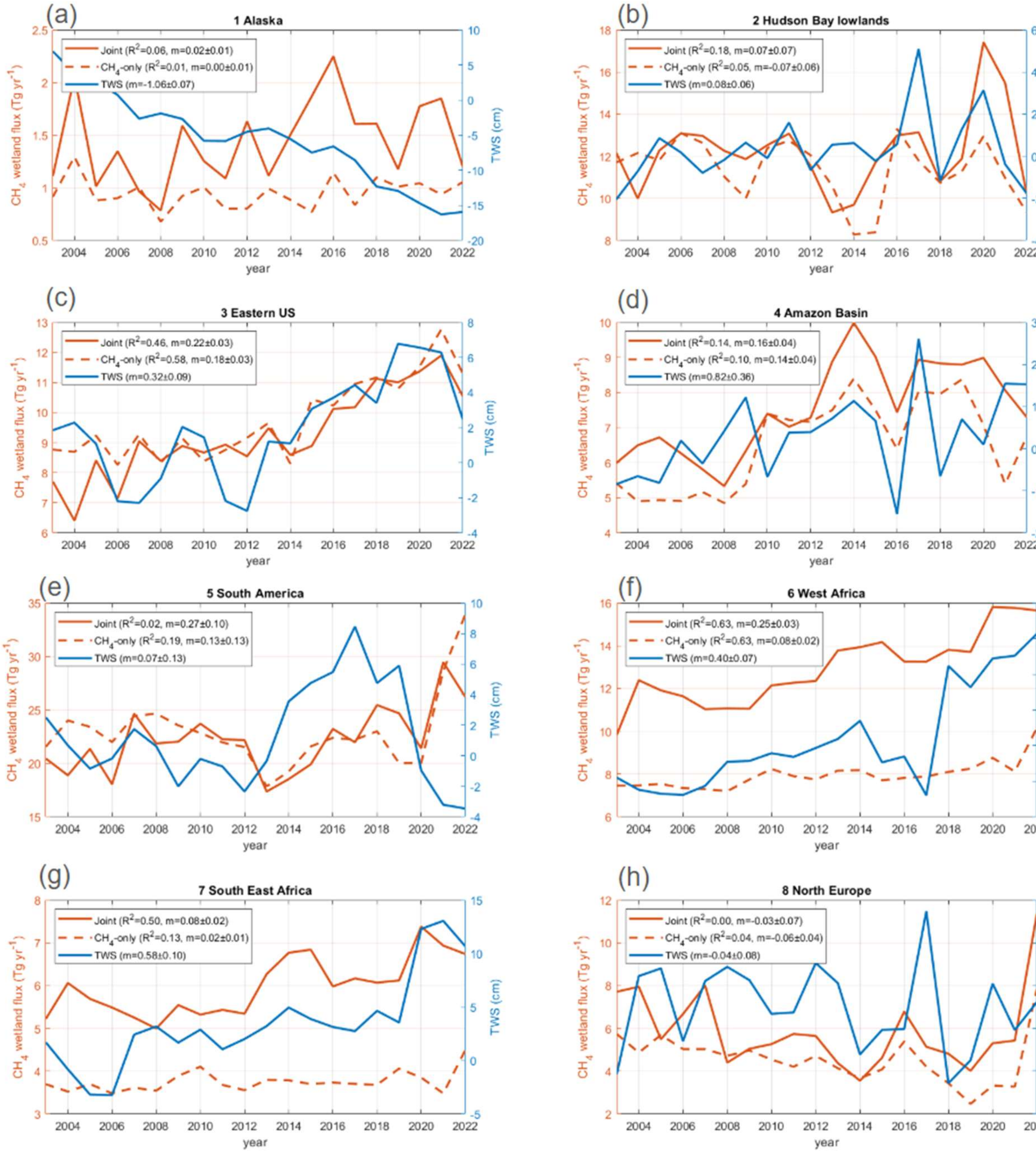

(a)
1 Alaska
Joint (R2=0.06, m=0.02±0.01)
CH4-only (R2=0.01, m=0.00±0.01)
TWS (m=-1.06±0.07)
(b)
2 Hudson Bay lowlands
Joint (R2=0.18, m=0.07±0.07)
CH4-only (R2=0.05, m=-0.07±0.06)
TWS (m=0.08±0.06)
(c)
3 Eastern US
Joint (R2=0.46, m=0.22±0.03)
CH4-only (R2=0.58, m=0.18±0.03)
TWS (m=0.32±0.09)
(d)
4 Amazon Basin
Joint (R2=0.14, m=0.16±0.04)
CH4-only (R2=0.10, m=0.14±0.04)
TWS (m=0.82±0.36)
(e)
5 South America
Joint (R2=0.02, m=0.27±0.10)
CH4-only (R2=0.19, m=0.13±0.13)
TWS (m=0.07±0.13)
(f)
6 West Africa
Joint (R2=0.63, m=0.25±0.03)
CH4-only (R2=0.63, m=0.08±0.02)
TWS (m=0.40±0.07)
(g)
7 South East Africa
Joint (R2=0.50, m=0.08±0.02)
CH4-only (R2=0.13, m=0.02±0.01)
TWS (m=0.58±0.10)
(h)
8 North Europe
Joint (R2=0.00, m=-0.03±0.07)
CH4-only (R2=0.04, m=-0.06±0.04)
TWS (m=-0.04±0.08)
CH4 wetland flux (Tg yr-1)
TWS (cm)
year

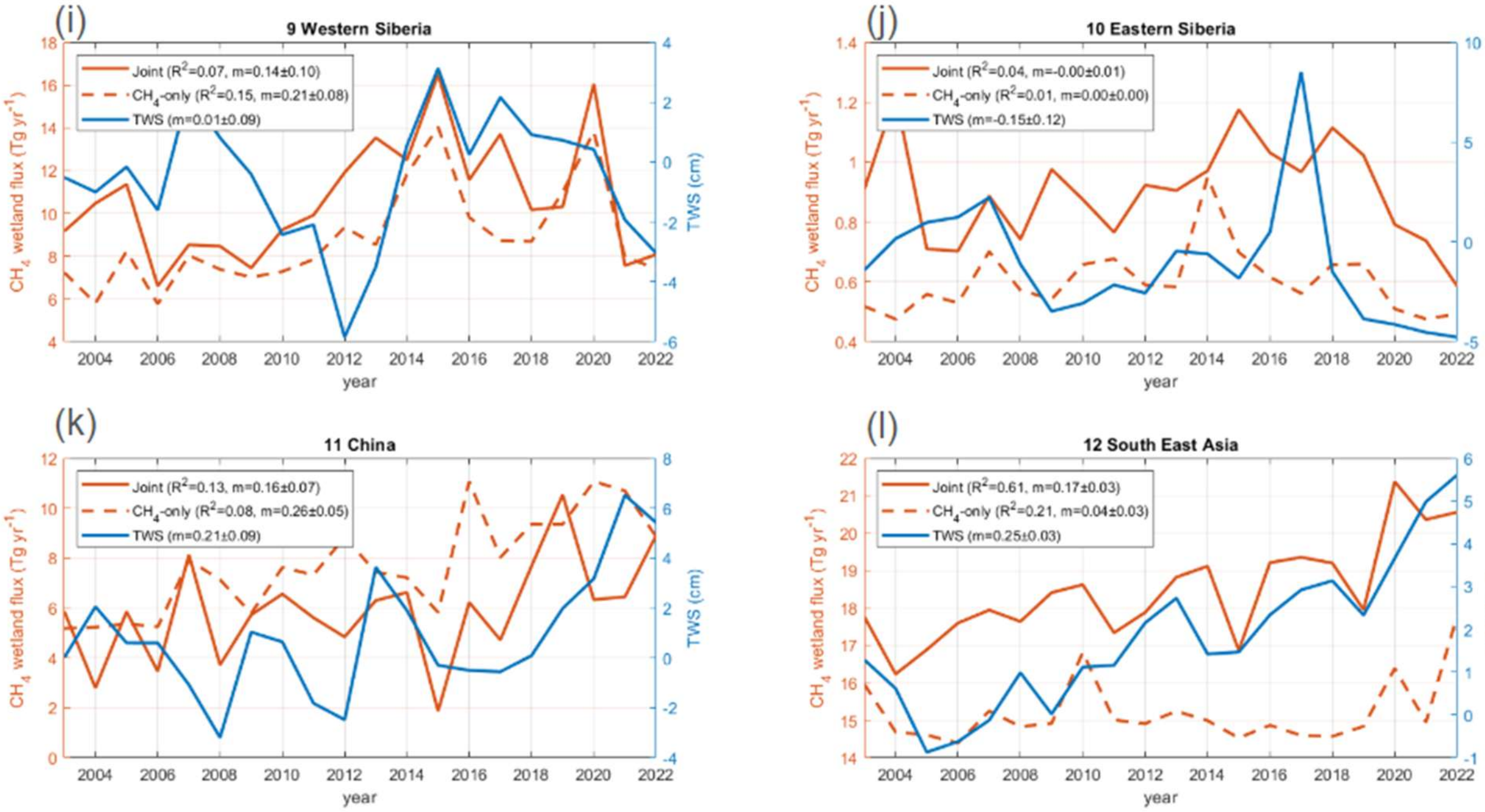

Figure S19 Temporal Variations between optimized wetland emissions and terrestrial water storage (TWS) from 2003 to 2022. A correlation analysis between regional optimized wetland CH_4 emissions (in $TgCH_4$ yr^{-1}) and TWS (in cm) from major wetland regions (Fig. 4a) for CH_4-only (red dashed) and Joint scenario (red solid). Each panel includes the long-term trend (slope, m) for each parameter and the correlation coefficients (R^2) between wetland emissions and TWS in the legend.

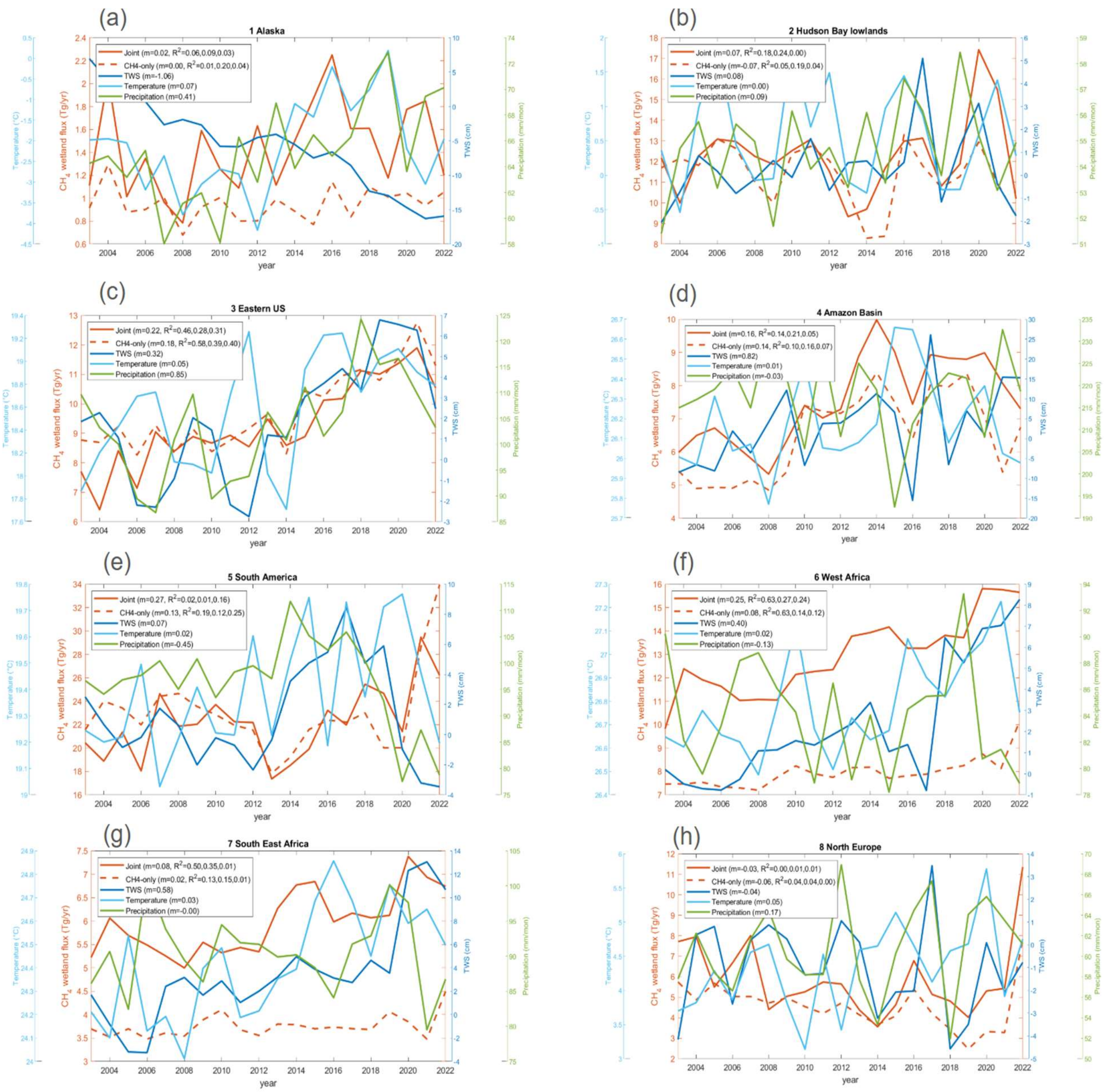
(a)
1 Alaska
Joint (m=0.02, R²=0.06,0.09,0.03)
CH4-only (m=0.00, R²=0.01,0.20,0.04)
TWS (m=-1.06)
Temperature (m=0.07)
Precipitation (m=0.41)
(b)
2 Hudson Bay lowlands
Joint (m=0.07, R²=0.18,0.24,0.00)
CH4-only (m=-0.07, R²=0.05,0.19,0.04)
TWS (m=0.08)
Temperature (m=0.00)
Precipitation (m=0.09)
(c)
3 Eastern US
Joint (m=0.22, R²=0.46,0.28,0.31)
CH4-only (m=0.18, R²=0.58,0.39,0.40)
TWS (m=0.32)
Temperature (m=0.05)
Precipitation (m=0.85)
(d)
4 Amazon Basin
Joint (m=0.16, R²=0.14,0.21,0.05)
CH4-only (m=0.14, R²=0.10,0.16,0.07)
TWS (m=0.82)
Temperature (m=0.01)
Precipitation (m=-0.03)
(e)
5 South America
Joint (m=0.27, R²=0.02,0.01,0.16)
CH4-only (m=0.13, R²=0.19,0.12,0.25)
TWS (m=0.07)
Temperature (m=0.02)
Precipitation (m=-0.45)
(f)
6 West Africa
Joint (m=0.25, R²=0.63,0.27,0.24)
CH4-only (m=0.08, R²=0.63,0.14,0.12)
TWS (m=0.40)
Temperature (m=0.02)
Precipitation (m=-0.13)
(g)
7 South East Africa
Joint (m=0.08, R²=0.50,0.35,0.01)
CH4-only (m=0.02, R²=0.13,0.15,0.01)
TWS (m=0.58)
Temperature (m=0.03)
Precipitation (m=-0.00)
(h)
8 North Europe
Joint (m=-0.03, R²=0.00,0.01,0.01)
CH4-only (m=-0.06, R²=0.04,0.04,0.00)
TWS (m=-0.04)
Temperature (m=0.05)
Precipitation (m=0.17)
CH4 wetland flux (Tg/yr)
Temperature (°C)
TWS (cm)
Precipitation (mm/mon)
year

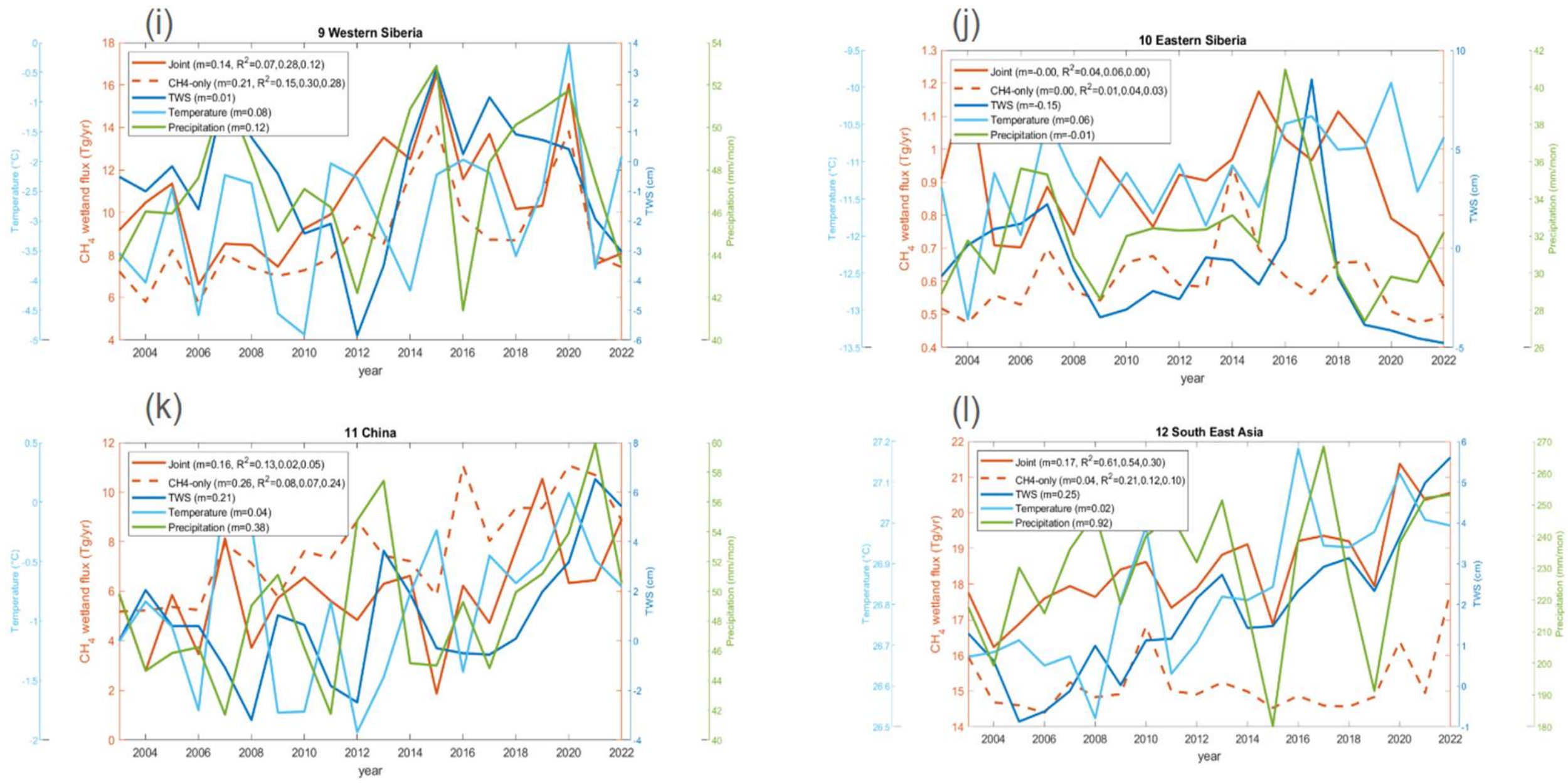

Figure S20 Temporal Variations between optimized wetland emissions and temperature, precipitation, and terrestrial water storage (TWS) from 2003 to 2022. A correlation analysis between regional optimized wetland CH_4 emissions ($TgCH_4\ yr^{-1}$) from major wetland regions (panel a in Fig. 4a) for CH_4-only (red dashed) and Joint (red solid) scenarios, soil TWS (blue, cm), 2-m air temperature (skyblue, K), and precipitation (green, mm mon^{-1}). Each panel includes the long-term trend (slope, m) for each parameter and the correlation coefficients (R^2) between wetland emissions and TWS, temperature, and precipitation, in that order, as shown in the legend.

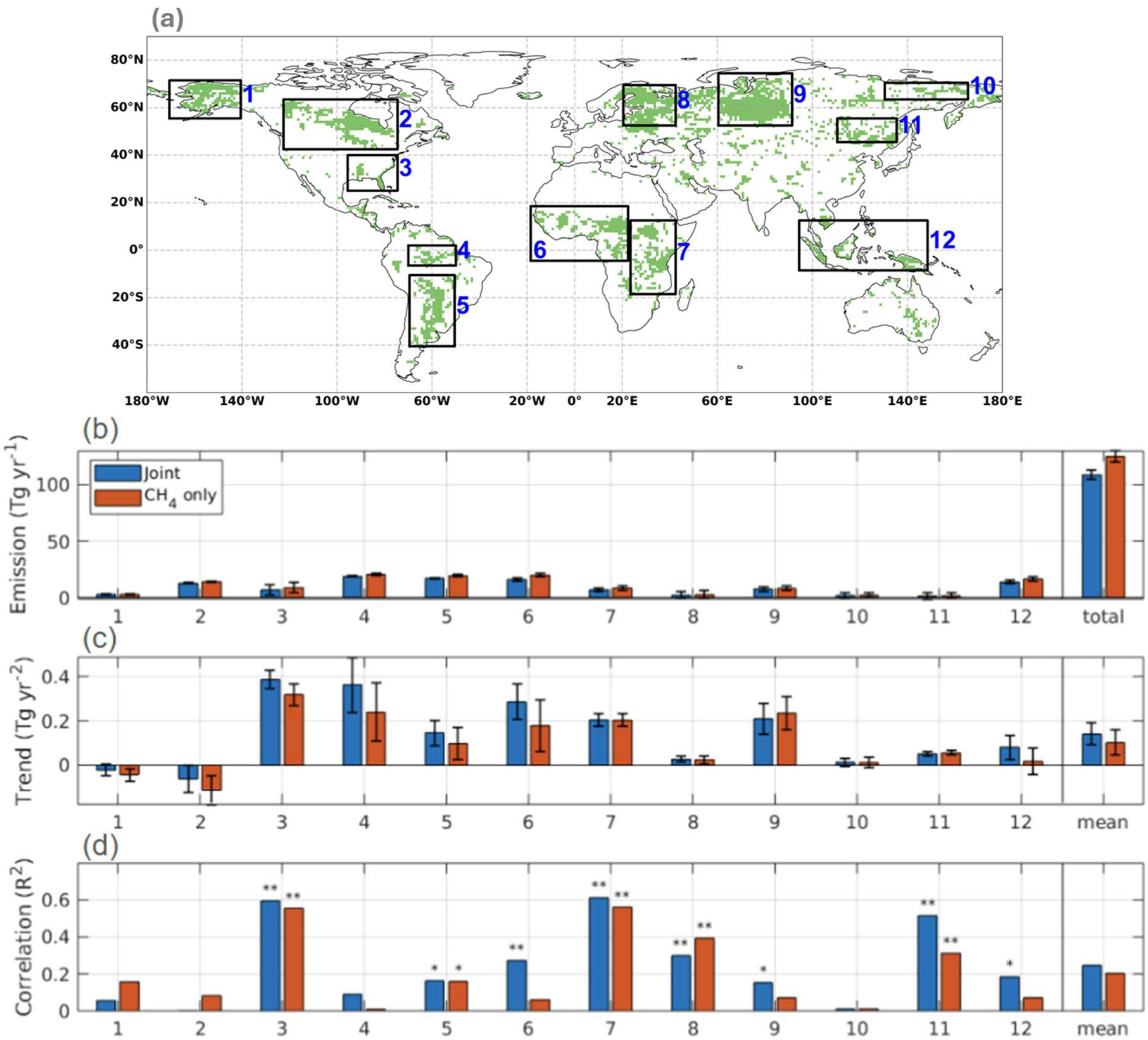

Figure S21 Mean and trends of optimized wetland CH_4 emissions using wetland priors with dynamic inundation and their correlation with terrestrial water storage from 2003 to 2022. (a) Spatial map of major wetland regions (green): 1 = Alaska, 2 = Hudson Bay, 3 = Southeast US, 4 = Amazon, 5 = South America, 6 = West Africa, 7 = Southeast Africa, 8 = Europe, 9 = West Siberia, 10 = East Siberia, 11 = North China, 12 = Southeast Asia. (b) Mean wetland emissions in $TgCH_4$ yr^{-1} from wetland regions (panel a) and total (rightmost) from inversions with (blue) and without (red) isotopic constraints. Uncertainty is estimated from the ensemble simulation (Supplementary Information). (c) Emission trends in $TgCH_4$ yr^{-2} by wetland regions (panel a) and the average (rightmost) for inversions with (blue) and without (red) isotopic constraints. Uncertainty from 1 standard deviation of the trends. (d) Coefficient of determination (R^2) between regional wetland CH_4 emissions and terrestrial water storage (cm) from major wetlands (panel a) and their R^2 mean (rightmost) for inversions with (blue) and without (red) isotopic constraints. Asterisks indicate significance (* < 0.1, ** < 0.05).

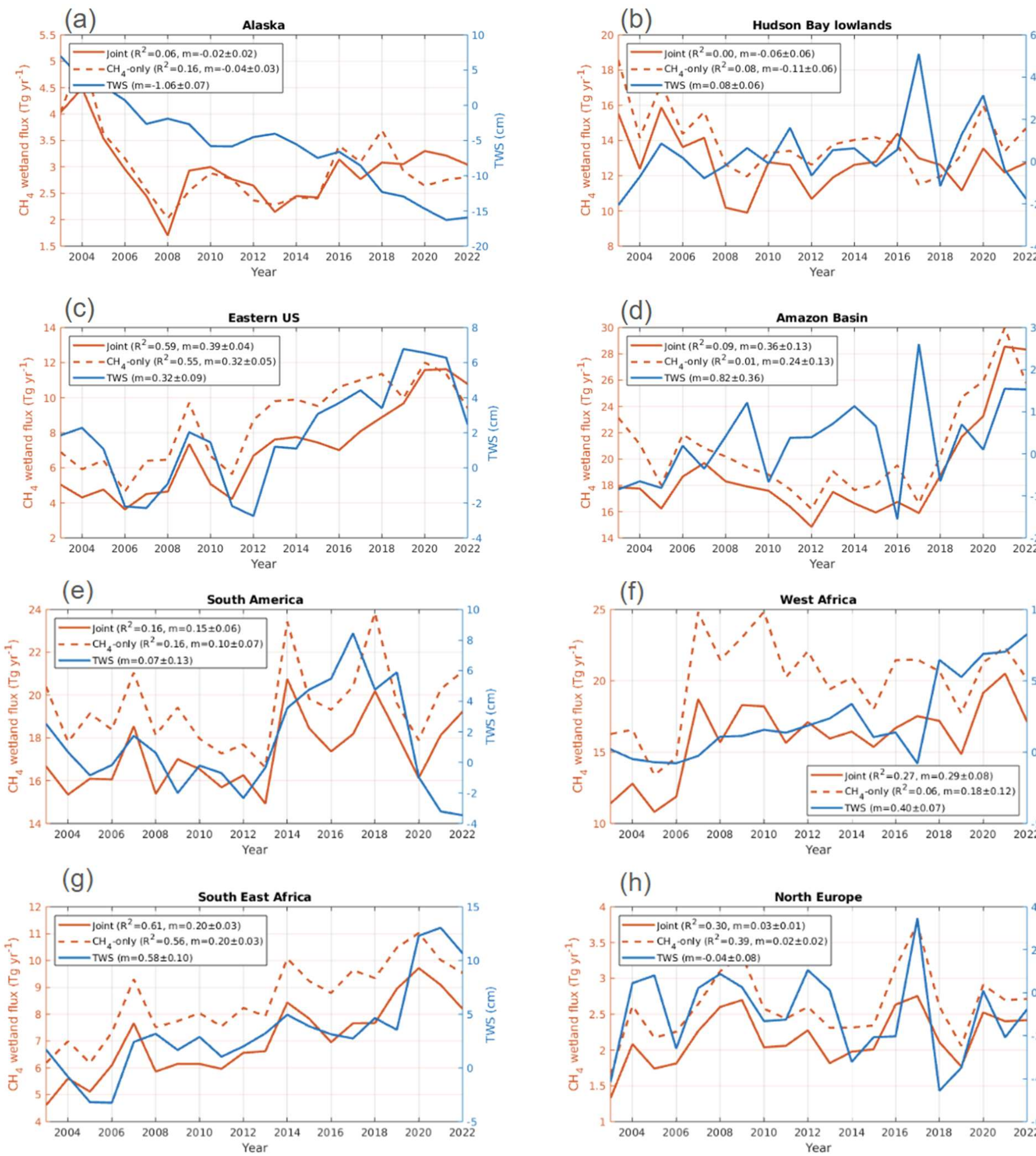
(a)
Alaska
Joint (R²=0.06, m=-0.02±0.02)
CH₄-only (R²=0.16, m=-0.04±0.03)
TWS (m=-1.06±0.07)
(b)
Hudson Bay lowlands
Joint (R²=0.00, m=-0.06±0.06)
CH₄-only (R²=0.08, m=-0.11±0.06)
TWS (m=0.08±0.06)
(c)
Eastern US
Joint (R²=0.59, m=0.39±0.04)
CH₄-only (R²=0.55, m=0.32±0.05)
TWS (m=0.32±0.09)
(d)
Amazon Basin
Joint (R²=0.09, m=0.36±0.13)
CH₄-only (R²=0.01, m=0.24±0.13)
TWS (m=0.82±0.36)
(e)
South America
Joint (R²=0.16, m=0.15±0.06)
CH₄-only (R²=0.16, m=0.10±0.07)
TWS (m=0.07±0.13)
(f)
West Africa
Joint (R²=0.27, m=0.29±0.08)
CH₄-only (R²=0.06, m=0.18±0.12)
TWS (m=0.40±0.07)
(g)
South East Africa
Joint (R²=0.61, m=0.20±0.03)
CH₄-only (R²=0.56, m=0.20±0.03)
TWS (m=0.58±0.10)
(h)
North Europe
Joint (R²=0.30, m=0.03±0.01)
CH₄-only (R²=0.39, m=0.02±0.02)
TWS (m=-0.04±0.08)
CH₄ wetland flux (Tg yr⁻¹)
TWS (cm)
Year

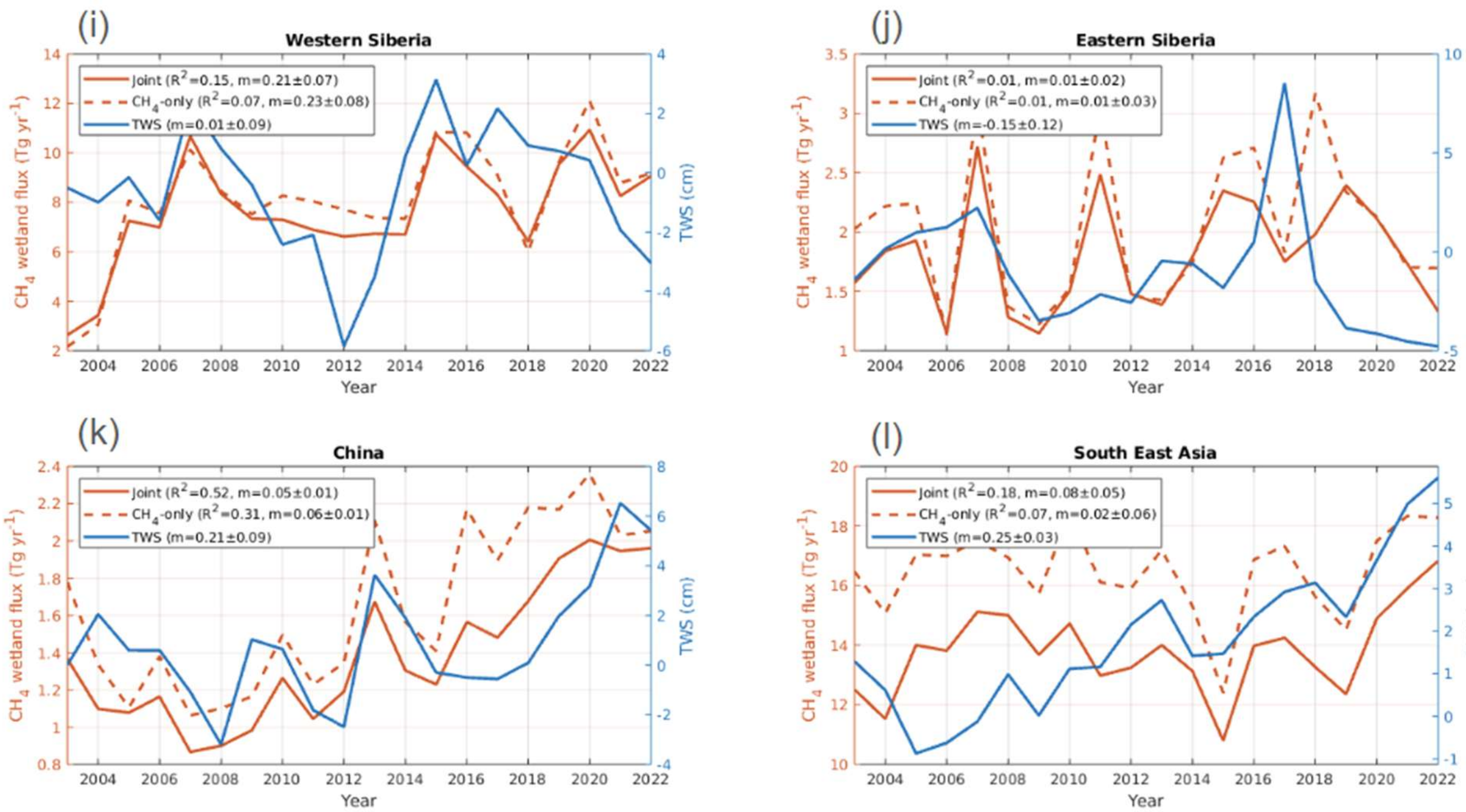

Figure S22 Temporal Variations between optimized wetland emissions with dynamic inundation and terrestrial water storage (TWS) from 2003 to 2022. A correlation analysis between regional optimized wetland CH_4 emissions using wetland priors with dynamic inundation (in TgCH_4 yr^{-1}) and TWS (in cm) from major wetland regions (Fig. 4a) for CH_4-only (red dashed) and Joint scenario (red solid). Each panel includes the long-term trend (slope, m) for each parameter and the correlation coefficients (R^2) between wetland emissions and TWS in the legend.

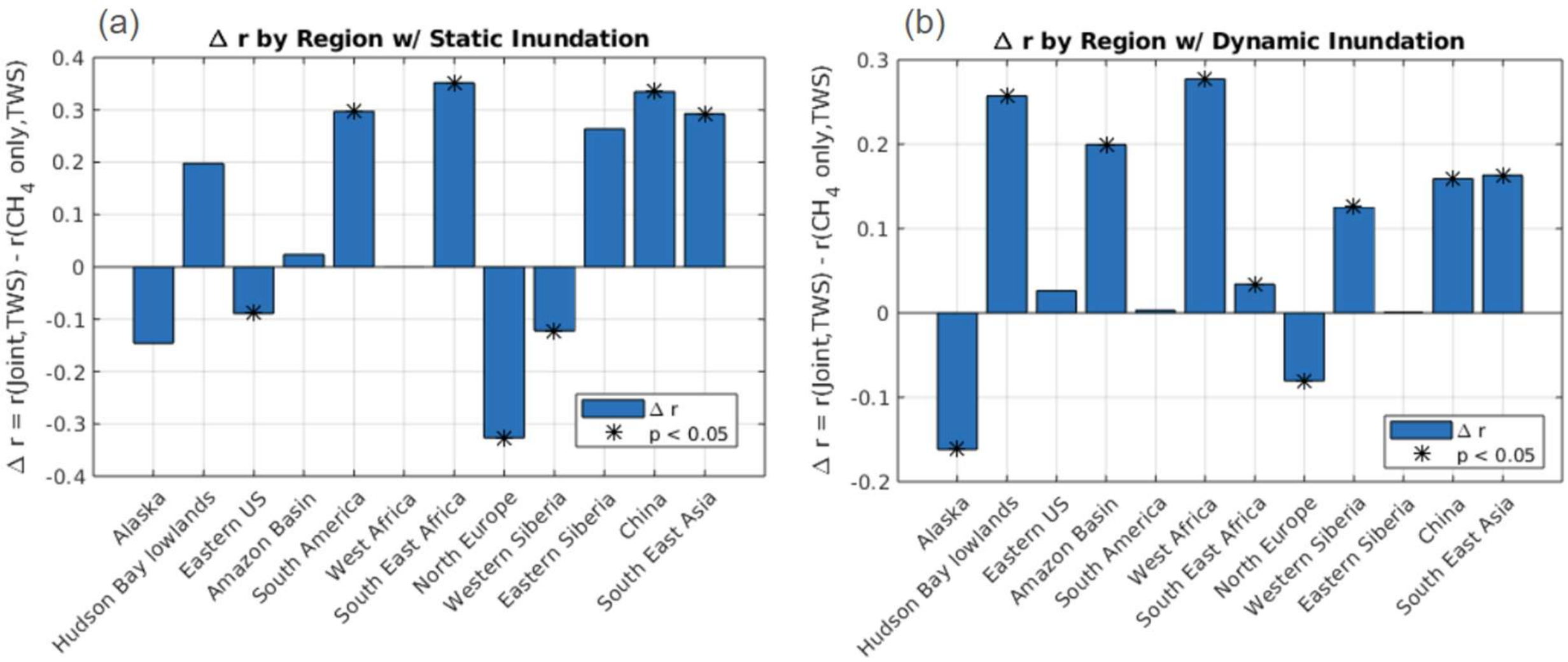

Figure S23 Differences in correlations between terrestrial water storage and optimized wetland CH_4 emissions in Joint and CH_4-only scenarios. Differences in correlation coefficients (Δr) between terrestrial water storage (TWS) and regional optimized wetland CH_4 emissions from the Joint scenario (r(Joint,TWS)) and the CH_4-only scenario (r(CH_4-only,TWS)) are shown using wetland priors derived from (a) static inundation and (b) dynamic inundation across the 12 major wetland regions (Fig. 4a). Star symbols indicate statistically significant differences in Δr ($p < 0.05$).

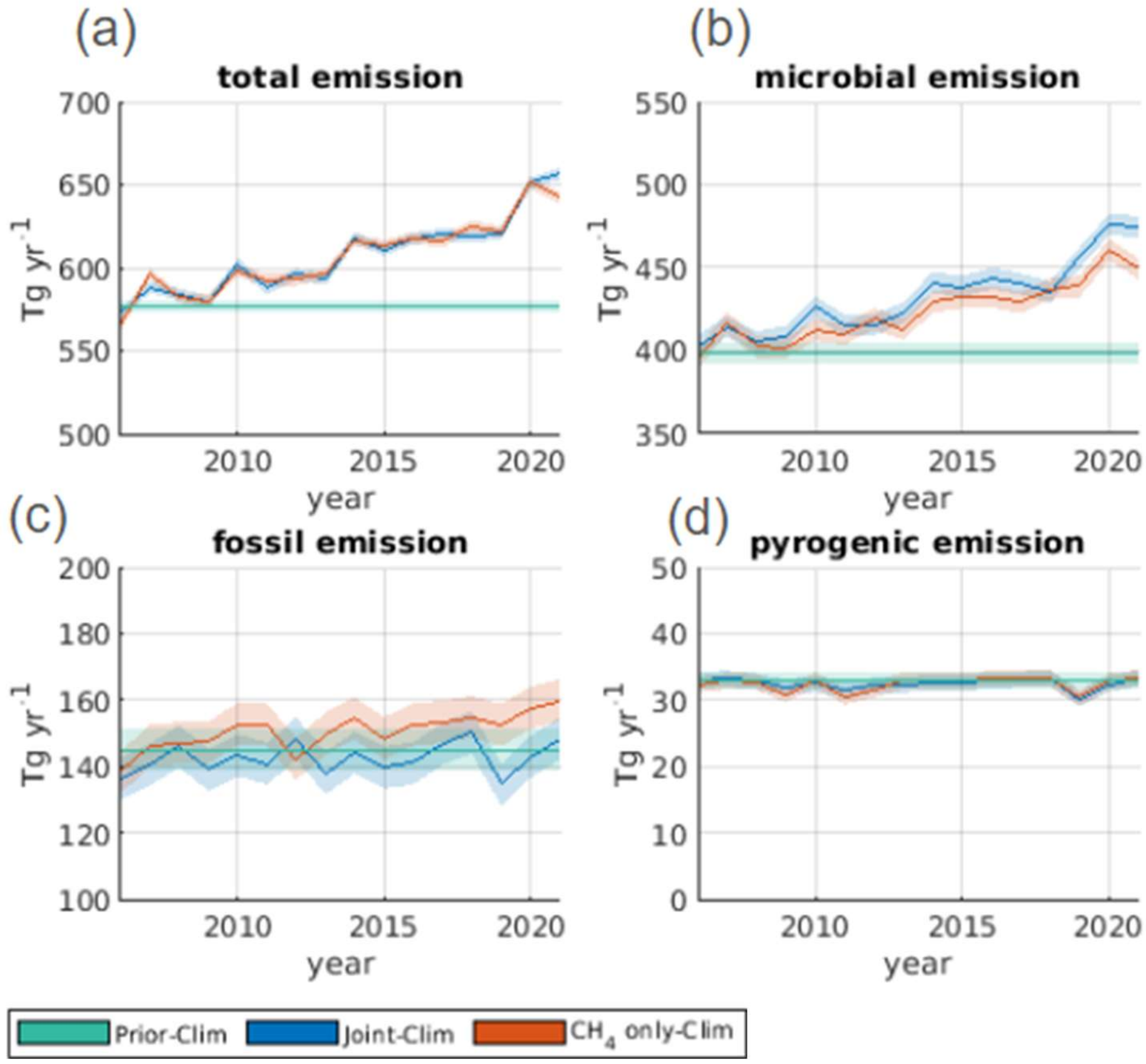

Figure S24 Global annual emissions of sources using climatological priors. (a-d) Long-term changes in CH_4 emissions ($TgCH_4$ yr^{-1}) from (a) total, (b) microbial, (c) fossil, and (d) pyrogenic sources for prior (Green), Joint+climatological prior (Blue), and CH_4-only+climatological priors (Red) scenarios during 2006-2021.

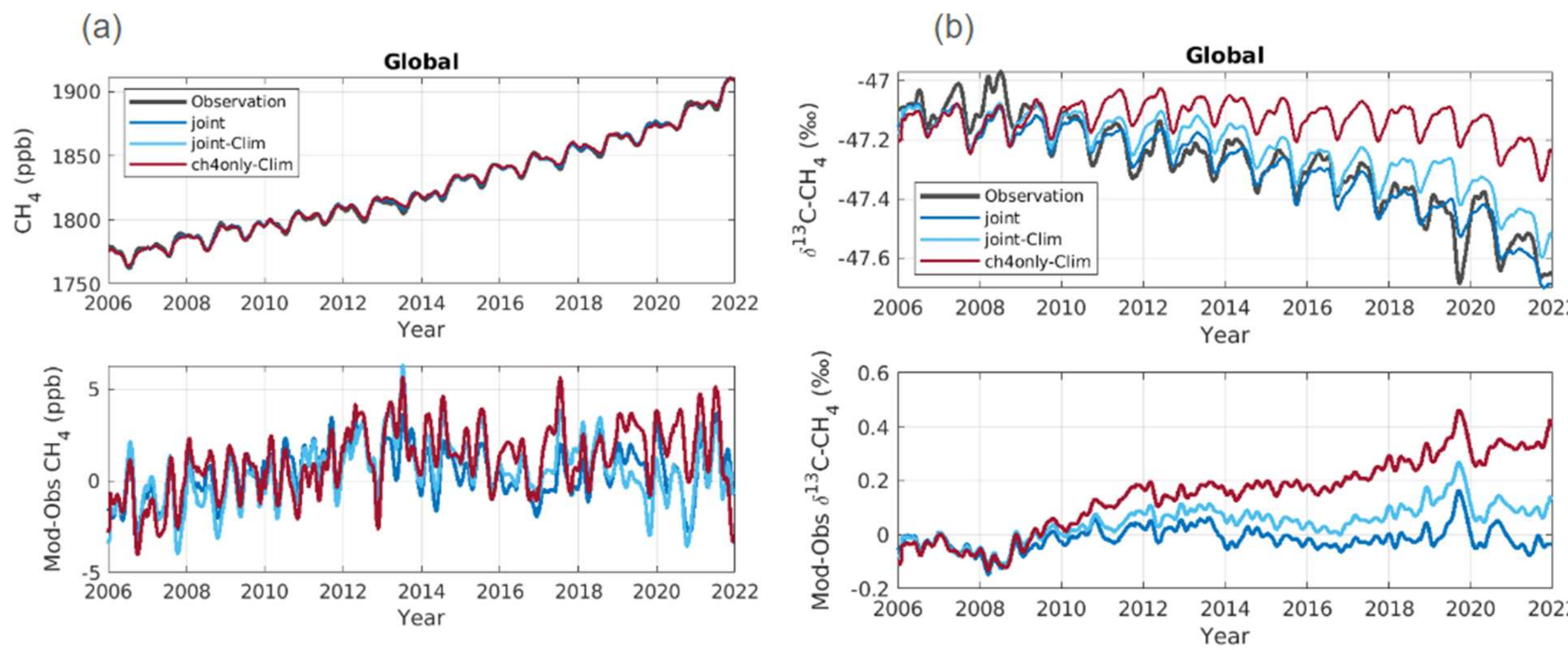

Figure S25 Global observed and simulated long-term trends in atmospheric CH_4 and $\delta^{13}C$-CH_4. Observed and simulated averaged (a) CH_4 in ppb and (b) $\delta^{13}C$-CH_4 in ‰ in 2000-2021. For each sub-plots in (a)-(b), (top) time series of averaged values and (bottom) difference between simulated and observed values for observed (black) and posterior from Joint (blue), Joint+climatological priors (skyblue), and CH_4-only+climatological priors (magenta) scenarios.

Supplementary Tables

Table S1 Review of previous atmospheric inversion papers on post-2006, post-2014, and 2020-2022 methane increase.

Table S2 Flux-weighted source signature uncertainty estimated from (Lan et al. 2021).

Table S3 Prior setup of sources.

Table S4 Setup of sinks. The coefficients C and D in the last column correspond to Eq. 2.

Table S5 Correlation Analysis between optimized wetland emissions and environmental parameters during 2003-2022. Coefficient of determination (R^2) between optimized wetland emissions from Joint (2-4' columns) and CH_4-only (5-7' columns) scenarios and terrestrial water storage (TWS) (2,5' columns), temperature (3,6' columns), and precipitation (4-7' columns) from major wetland regions (panel a in Fig. 4a).

Table S6. Model-data mismatch and selection of assimilation and validation sites for atmospheric CH_4 and $\delta^{13}C$-CH_4 data

Table S1. Review of previous 3-D atmospheric inversion papers on rapid CH_4 growth during 2007-2013, 2014-2017, and 2020-2022.

Ref.	Model	Optimizer	Observation	Cause of 2007-2013 methane increase	Cause of 2014-2017 methane increase	Cause of 2020-2022 methane increase
Chandra et al. 2021	MIROC4-ACTM	Bayesian analytic time-dependent inversion	In-situ methane	Enhanced emissions from the coal mining industry, mainly in China.<br><br>Increased ruminant emissions in the tropics, specifically in South Asia, Brazil, and North Africa.		
Drinkwater 2023	GEOS-Chem	EnKF and Bayesian inversion	In-situ methane, GOSAT			Increases in tropical CH_4 emissions are from biogenic sources, with a significant fraction from wetlands.
Feng 2023	GEOS-Chem	EnKF	GOSAT			Increasing wetland emissions and a significant reduction in OH level.
Lin 2024	PYVAR-LMDZ-SACS	Variational Bayesian inversion	In-situ methane, GOSAT			Major contribution from tropical and boreal wetland emissions associated with enhanced inundation area.
Lunt 2019	GEOS-Chem	Hierarchical Bayesian inversion method	GOSAT		Increases in tropical African CH_4 emissions align with expanded wetland extent driven by increased White Nile inflow (Not a global study; focused on tropical Africa).	
Niwa 2024	NISCOM-CH4	4DVar	In-situ methane, GOSAT			Enhanced biogenic emissions, particularly wetland, agriculture, and waste in the tropics (especially tropical South America) and northern low-latitude (particularly south asia and northern Southeast asia), and limited role of OH in the Asian region
Pandey 2017	TM5-4DVar	4DVar	GOSAT		Enhanced tropical CH_4 emissions during 2011 La Nina are attributed to increased biogenic emissions particularly wetland.	
Peng 2022		PYVAR-LMDZ-SACS	In-situ methane, GOSAT			Increase in 2020 is due to natural emission increase and OH changes.

Qu 2022	GEOS-Chem	Bayesian analytical solution	GOSAT			Increased emissions and decreased OH contribute 86% and 14% respectively, to the anomolus increase to 2020 CH_4, and half of the emissions are explained by extended wetland emissions from Africa.
Qu 2024	GEOS-Chem	Bayesian analytical solution	GOSAT		Africa accounts for 43% of the total emission increase.	2019-2022, Africa's contribution increases mainly due to anthropogenic and wetland sources.
Thanwerdas 2024	LMDz-CIF	TM5-4DVar	In-situ (methane, $\delta^{13}C$, δD)	Increases in emissions from (1) fossil sources (51 % of the net increase in emissions) and (2) agriculture and waste sources (49 %), which were slightly compensated for by a small decrease in biofuel- and biomass-burning emissions.		
Tsuruta 2017	CTE-CH_4	EnKF	In-situ methane, GOSAT	Depending on the inversion setup, the result was due to anthropogenic or biospheric fluxes. The enhanced emissions are mainly attributed to increased emissions from South American temperate, Asian temperate and Asian tropical regions		
Worden 2017	GEOS-Chem	Bayesian Markov Chain Monte Carlo	MOPITT CO	Biomass burning emissions of methane declined by 3.7 Tg CH_4 per year between 2001–2007 and 2008–2014. Updated methane budgets indicate that fossil fuels account for 12–19 Tg CH_4 per year of the recent atmospheric methane increase.		
Yin 2021	PYVAR-LMDz with LMDz-INCA	Bayesian variational inversion	In-situ CH_4 and CO, GOSAT X_{CH4}, MOPITT X_{CO}, OMI X_{HCHO}		Accelerating CH4 growth rate is mainly attributed to increased emissions particularly from tropical wetland as major contributor (35%) followed by anthropogenic emissions in China (20%).	
Zhang 2021	GEOS-Chem CTM v11.02	Bayesian analytical solution	GOSAT		Accelerated CH_4 increase is mainly attributed to anthropogenic emissions (particularly growing livestock populations) over South Asia, tropical Africa, and Brazil. They also found an insignificant contribution of OH to enhanced CH_4 concentrations.	

Table S2 Flux-weighted $\delta^{13}C$-CH_4 source signature uncertainty estimated from Lan et al. (2021)[16].

		Mean	Uncertainty
**Microbial**	**Wetland**	-61.3	0.06
	**Termite/Wild Animal**	-63.4	2.8
	**Ruminant (C3)**	-67.8	2.1
	**Ruminant (C4)**	-54.5	1.7
	**Wastewater/Landfill**	-55.6	1.7
	**Rice**	-60.5	1.1
**Fossil**	**Oil and Gas**	-44	0.5
	**Coal**	-44	1.5
	**Geologic Seep**	-49	1.5
**Pyrogenic**	**C3 vegetation**	-26.7	2.3
	**C4 vegetation**	-12.5	1.1

Table S3 Prior setup of sources.

<table>
<tr><th>Source</th><th>Source sector</th><th>Total emissions</th><th>$\delta^{13}C$-CH_4 source signatures</th></tr>
<tr><td rowspan="2">Fossil Emission</td><td>Coal, oil and natural gas, and other energy/industry</td><td>EDGAR 8.0[17]</td><td>Sherwood et al. 2017; Sherwood et al. 2021[18,19]</td></tr>
<tr><td>Geological seeps</td><td>Etiope et al. 2019[20]</td><td>Etiope et al. 2019[20]</td></tr>
<tr><td rowspan="2">Biomass and biofuel burning</td><td>Biomass burning fluxes</td><td>GFED 4.1s[21,22]</td><td>Still et al. 2003; Randerson et al. 2018[22,23]</td></tr>
<tr><td>Biofuel fluxes</td><td>EDGAR 8.0[17]</td><td>Still et al. 2003; Randerson et al. 2018[22,23]</td></tr>
<tr><td rowspan="5">Microbial Emission</td><td>Ruminants fluxes</td><td rowspan="3">EDGAR 8.0[17]</td><td>Still et al. 2003; Randerson et al. 2018[22,23]</td></tr>
<tr><td>Waste and landfills fluxes</td><td rowspan="3">Sherwood et al. 2017; Sherwood et al. 2021[18,19]</td></tr>
<tr><td>Rice fluxes</td></tr>
<tr><td>Wild animals and termites fluxes</td><td>Bergamaschi et al. 2007[24]</td></tr>
<tr><td>Wetland and soil sink</td><td>Based on Liu et al. 2020[25] and static inundation data[26]</td><td>Ganesan et al. 2018[27]</td></tr>
</table>

Table S4 Setup of sinks. The coefficients C and D in the last column correspond to Eq. 2.

Sink	Location	Distribution	Fractionation
**Loss of OH**	Troposphere	Spivakovsky et al. 2000; Montzka et al. 2011[14,15]	C = 1.0054, D = 0.00 Cantrell et al. 1990[28]
	Stratosphere	Jöckel et al. 2006[29]	
**Loss of Cl**	Troposphere	Hossaini et al. 2016[30]	C = 1.043, D = 6.46 Saueressig et al. 1995[31]
	Stratosphere	Jöckel et al. 2006[29]	
**Loss of O^1D**	Stratosphere	Jöckel et al. 2006[29]	C = 1.013, D = 0.00 Saueressig et al. 2001[32]
**Soil sink**	Terrestrial	Zhuang et al. 2013; Liu et al. 2020[25,33]	C = 1.021, D = 0.00 King et al. 1989[34]

Table S5 Correlation Analysis between optimized wetland emissions and environmental parameters during 2003-2022. Coefficient of determination (R^2) between optimized wetland emissions from Joint (2-4' columns) and CH_4-only (5-7' columns) scenarios and terrestrial water storage (TWS) (2,5' columns), temperature (3,6' columns), and precipitation (4-7' columns) from major wetland regions (panel a in Fig. 4a).

	**Joint**			**CH_4-only**		
	**TWS**	**Precipitation**	**Temperature**	**TWS**	**Precipitation**	**Temperature**
**1. Alaska**	0.063	0.029	0.089	0.011	0.037	0.203
**2. Hudson Bay lowlands**	0.178	0.001	0.235	0.050	0.043	0.189
**3. Eastern US**	0.456	0.310	0.283	0.581	0.404	0.394
**4. Amazon Basin**	0.140	0.054	0.214	0.100	0.072	0.163
**5. South America**	0.018	0.159	0.007	0.187	0.246	0.120
**6. West Africa**	0.625	0.236	0.269	0.626	0.122	0.135
**7. South East Africa**	0.503	0.010	0.353	0.128	0.005	0.146
**8. North Europe**	0.003	0.010	0.011	0.045	0.000	0.042
**9. Western Siberia**	0.073	0.116	0.283	0.154	0.276	0.302
**10. Eastern Siberia**	0.035	0.001	0.064	0.008	0.031	0.036
**11. China**	0.134	0.053	0.018	0.080	0.243	0.073
**12. South East Asia**	0.612	0.298	0.542	0.212	0.104	0.119

Table S6. Selection of assimilation and validation sites and their model-data mismatch for atmospheric (a) CH_4 and (b) $\delta^{13}C$-CH_4 data[35].

(a)

Assimilated

	**Site Code**	**Laboratory**	**Mean Latitude**	**Mean Longitude**	**Mean Elevation (MASL)**	**No. of Observation**	**Model-dat Mismatch (ppb)**
**1**	ABP ch4 surface event IPEN	IPEN	-12.77	-38.17	15	186	7.82
**2**	ABP ch4 surface event NOAA	NOAA	-12.77	-38.17	6	222	5.42
**3**	ABT ch4 surface hourly EC	EC	49.01	-122.34	93	51389	40.89
**4**	ALF ch4 aircraft event IPEN	IPEN	-8.86	-56.75	2229.37	1931	15.11
**5**	ALK ch4 aircraft event TU	TU	65.89	-152.1	2067.39	64	13.86
**6**	ALT ch4 surface event MPI-BGC	MPI-BGC	82.45	-62.51	185	432	12.85
**7**	ALT ch4 surface event NOAA	NOAA	82.45	-62.51	190	3562	13.45
**8**	ALT ch4 surface hourly EC	EC	82.45	-62.51	195	150696	12.66
**9**	AMS ch4 surface event LSCE	LSCE	-37.8	77.54	72.61	882	5.27
**10**	AMS ch4 surface hourly LSCE	LSCE	-37.8	77.54	75	12761	5
**11**	AMT ch4 surface event NOAA	NOAA	45.03	-68.68	160.11	2259	23.34
**12**	AMY ch4 surface event NOAA	NOAA	36.54	126.33	87	804	47.13
**13**	AMY ch4 surface hourly KMA	KMA	36.54	126.33	47	148695	58.15
**14**	AOA ch4 aircraft event JMA	JMA	28.22	148.17	5351.89	2746	17.9
**15**	AOC ch4 surface event NOAA	NOAA	-2.76	-24.97	12.41	234	13.61
**16**	ARH ch4 surface event NIWA	NIWA	-77.83	166.2	184	189	5
**17**	ASC ch4 surface event NOAA	NOAA	-7.97	-14.4	90	4316	8.42
**18**	ASK ch4 surface event NOAA	NOAA	23.26	5.63	2715	1984	11.21
**19**	AZR ch4 surface event NOAA	NOAA	38.76	-27.29	24	1553	17.5
**20**	AZV ch4 surface hourly NIES	NIES	54.71	73.03	160	10548	47.68
**21**	BAL ch4 surface event NOAA	NOAA	55.42	16.97	28	2566	41.28
**22**	BAO ch4 surface event NOAA	NOAA	40.05	-105	1884	3066	65.84
**23**	BCK ch4 surface hourly EC	EC	62.8	-115.92	220	76883	25.6
**24**	BGI ch4 aircraft event NOAA	NOAA	42.82	-94.41	4221.49	355	18.64
**25**	BGU ch4 surface event LSCE	LSCE	41.97	3.23	13.67	536	25.07
**26**	BHD ch4 surface event NIWA	NIWA	-41.41	174.87	85	311	6.68
**27**	BHD ch4 surface event NOAA	NOAA	-41.41	174.87	91.41	615	5.65
**28**	BIK ch4 surface event MPI-BGC	MPI-BGC	53.2	22.75	183	414	62.37
**29**	BIR ch4 surface hourly NILU	NILU	58.39	8.25	218	8317	18.88
**30**	BIS ch4 surface hourly LSCE	LSCE	44.38	-1.23	214	86947	20.91
**31**	BKT ch4 surface event NOAA	NOAA	-0.2	100.32	875	1546	28.46
**32**	BKT ch4 surface hourly BMKG	BMKG	-0.2	100.32	896	39862	56.25

**33**	BKT ch4 surface hourly EMPA	EMPA	-0.2	100.32	896.5	8061	66.56
**34**	BLK ch4 surface hourly EC	EC	64.33	-96.01	61	19836	19.02
**35**	BME ch4 surface event NOAA	NOAA	32.37	-64.65	17	777	17.21
**36**	BMW ch4 surface event NOAA	NOAA	32.26	-64.88	56.26	1899	13.99
**37**	BNE ch4 aircraft event NOAA	NOAA	40.8	-97.18	3736.54	1098	20.14
**38**	BRA ch4 surface hourly EC	EC	50.2	-104.71	630	80974	45.49
**39**	BRW ch4 surface event NOAA	NOAA	71.32	-156.6	21.18	4803	17.08
**40**	BRW ch4 surface hourly NOAA	NOAA	71.32	-156.61	27.67	94835	19.54
**41**	BRZ ch4 surface hourly NIES	NIES	56.15	84.33	248	22219	41.2
**42**	BSC ch4 surface event NOAA	NOAA	44.18	28.66	5	1356	62.46
**43**	BWD ch4 surface event NOAA	NOAA	38.93	-76.96	67.97	54	40.77
**44**	CAR ch4 aircraft event NOAA	NOAA	40.79	-104.57	4788.81	8536	15.52
**45**	CBA ch4 surface event NOAA	NOAA	55.21	-162.72	41.62	4377	13.63
**46**	CBW ch4 surface hourly ECN	ECN	51.97	4.93	200	47412	46.45
**47**	CBY ch4 surface hourly EC	EC	69.13	-105.06	47	66185	13.64
**48**	CDL ch4 surface hourly EC	EC	53.99	-105.12	630	31137	31.06
**49**	CEA ch4 aircraft event NIES	NIES	62.11	78.35	10612.34	887	42.59
**50**	CFA ch4 surface event CSIRO	CSIRO	-19.28	147.06	5	745	5.77
**51**	CGO ch4 surface event NOAA	NOAA	-40.68	144.68	164	1915	7.99
**52**	CHC ch4 surface hourly LSCE	LSCE	-16.2	-68.1	5355	49218	20.05
**53**	CHL ch4 surface event EC	EC	58.75	-94.07	95	711	58.9
**54**	CHL ch4 surface hourly EC	EC	58.74	-93.82	89	63471	52.29
**55**	CHM ch4 surface hourly EC	EC	49.69	-74.34	423	23872	28.31
**56**	CHR ch4 surface event NOAA	NOAA	1.7	-157.15	4.97	1423	13.42
**57**	CHS ch4 surface hourly NOAA	NOAA	68.51	161.53	64.4	108724	21.98
**58**	CIB ch4 surface event NOAA	NOAA	41.81	-4.93	849.99	1071	23.78
**59**	CMA ch4 aircraft event NOAA	NOAA	38.85	-74.31	3479.9	3273	20.39
**60**	CMN ch4 surface hourly IAFMS	IAFMS	44.17	10.68	2177	48861	19.07
**61**	CMN ch4 surface hourly ICOS	ICOS	44.19	10.7	2173	22346	19.58
**62**	CMN ch4 surface hourly UNIURB	UNIURB	44.17	10.68	2172	10299	29.26
**63**	CMO ch4 surface event NOAA	NOAA	45.48	-123.97	35	47	17.65
**64**	COI ch4 surface hourly NIES	NIES	43.16	145.5	94	8714	21.9
**65**	CON ch4 aircraft event NIES	NIES	4.08	142.67	11018.63	2400	15.34
**66**	CPA ch4 surface hourly EMPA	EMPA	42.64	77.07	1623	30097	29.41
**67**	CPS ch4 surface hourly EC	EC	49.82	-74.98	420.81	75148	30.87
**68**	CPT ch4 surface event NOAA	NOAA	-34.35	18.49	260	746	5
**69**	CPT ch4 surface hourly SAWS	SAWS	-34.35	18.49	260	140642	5
**70**	CRI ch4 surface event CSIRO	CSIRO	15.08	73.83	66	212	40.82
**71**	CRV ch4 surface hourly NOAA	NOAA	64.99	-147.6	643.13	237517	15.54

**72**	CRZ ch4 surface event NOAA	NOAA	-46.43	51.85	202	1862	5.7
**73**	CVO ch4 surface event MPI-BGC	MPI-BGC	16.86	-24.87	10	661	10.52
**74**	CVO ch4 surface hourly UOE	UOE	16.86	-24.87	31	53706	11.42
**75**	CYA ch4 surface event CSIRO	CSIRO	-66.28	110.52	55	766	5
**76**	DEM ch4 surface hourly NIES	NIES	59.79	70.87	126	14115	52.84
**77**	DEU ch4 surface hourly UBA	UBA	49.77	7.05	480	8442	38.65
**78**	DND ch4 aircraft event NOAA	NOAA	47.81	-98.55	3747.55	2127	18.98
**79**	DRP ch4 surface event NOAA	NOAA	-59.16	-62.59	10	641	5
**80**	DSI ch4 surface event NOAA	NOAA	20.7	116.73	8	1062	28.08
**81**	DWS ch4 surface hourly EC	EC	43.78	-79.47	218	90374	47.6
**82**	EGB ch4 surface hourly EC	EC	44.23	-79.78	270.54	112225	41.85
**83**	EIC ch4 surface event NOAA	NOAA	-27.15	-109.44	60.96	1561	6.66
**84**	ESP ch4 aircraft event NOAA	NOAA	49.41	-126.45	2746.03	5011	12.29
**85**	ESP ch4 surface event EC	EC	49.38	-126.55	79	1285	12.13
**86**	ESP ch4 surface hourly EC	EC	49.38	-126.54	47	98212	12.92
**87**	EST ch4 surface hourly EC	EC	51.67	-110.21	752.01	91009	69.52
**88**	ETL ch4 aircraft event NOAA	NOAA	54.31	-104.94	3089.39	3220	21.6
**89**	ETL ch4 surface hourly EC	EC	54.35	-104.99	598	118445	33.76
**90**	FKL ch4 surface event LSCE	LSCE	35.34	25.67	151.41	347	28.71
**91**	FKL ch4 surface hourly LSCE	LSCE	35.34	25.67	165	55471	29.94
**92**	FNE ch4 surface hourly EC	EC	58.84	-122.57	376	49095	41.4
**93**	FSD ch4 surface hourly EC	EC	49.88	-81.57	250	151131	40.37
**94**	FTL ch4 aircraft event NOAA	NOAA	-4.15	-38.28	2120.18	159	8.32
**95**	FWI ch4 aircraft event NOAA	NOAA	44.7	-91.01	4212.13	379	20.13
**96**	GAT ch4 surface hourly ICOS	ICOS	53.07	11.44	224.86	186927	35.05
**97**	GIF ch4 surface hourly LSCE	LSCE	48.71	2.15	167	50783	27.76
**98**	GMI ch4 surface event NOAA	NOAA	13.39	144.66	6.33	3228	15.56
**99**	GOZ ch4 surface event NOAA	NOAA	36.05	14.89	6	40	29.09
**100**	GPA ch4 surface event CSIRO	CSIRO	-12.25	131.04	37	107	20.87
**101**	HAT ch4 surface hourly NIES	NIES	24.06	123.81	47.3	8771	21.67
**102**	HBA ch4 surface event NOAA	NOAA	-75.61	-26.21	35	1896	6.26
**103**	HEL ch4 surface hourly ICOS	ICOS	54.18	7.88	153	1761	46.38
**104**	HFM ch4 aircraft event NOAA	NOAA	42.54	-72.17	3850.92	1676	18.58
**105**	HFM ch4 surface event NOAA	NOAA	42.54	-72.17	369	12	40
**106**	HIL ch4 aircraft event NOAA	NOAA	40.04	-87.94	4157.22	3978	20.03
**107**	HLE ch4 surface event LSCE	LSCE	32.78	78.96	4522	268	33.85
**108**	HNP ch4 surface hourly EC	EC	43.61	-79.39	97	45698	46.98
**109**	HPB ch4 surface event NOAA	NOAA	47.8	11.02	990	1549	49.79
**110**	HPB ch4 surface hourly DWD	DWD	47.8	11.02	1116	8640	47.45

**111**	HPB ch4 surface hourly ICOS	ICOS	47.8	11.02	1027.94	133719	59.45
**112**	HSU ch4 surface event NOAA	NOAA	41.05	-124.6	7.6	168	20.95
**113**	HTM ch4 surface hourly ICOS	ICOS	56.1	13.42	198.44	107785	34.83
**114**	HUN ch4 surface event NOAA	NOAA	46.95	16.65	344	2391	37.04
**115**	HUN ch4 surface hourly ELTE	ELTE	46.95	16.65	344	33597	37.44
**116**	ICE ch4 surface event NOAA	NOAA	63.4	-20.29	125.88	2323	16.51
**117**	IGR ch4 surface hourly NIES	NIES	63.19	64.42	56	15135	72.94
**118**	INU ch4 surface hourly EC	EC	68.32	-133.53	123	81970	18.69
**119**	INX ch4 surface event NOAA	NOAA	39.58	-86.42	376.73	2297	41.56
**120**	IPR ch4 surface hourly ICOS	ICOS	45.81	8.63	289.94	91991	94.6
**121**	IPR ch4 surface hourly IES	IES	45.81	8.63	239	57160	83.7
**122**	ITN ch4 surface event NOAA	NOAA	35.37	-77.39	505	228	27.96
**123**	IZO ch4 surface event NOAA	NOAA	28.3	-16.49	2377.9	2222	11.49
**124**	IZO ch4 surface hourly AEMET	AEMET	28.31	-16.5	2398.2	161589	12.63
**125**	JFJ ch4 surface event MPI-BGC	MPI-BGC	46.55	7.99	3570	327	15.98
**126**	JFJ ch4 surface hourly EMPA	EMPA	46.55	7.99	3580	122515	18.32
**127**	JFJ ch4 surface hourly ICOS	ICOS	46.55	7.99	3575	36049	16.5
**128**	JGS ch4 surface hourly KMA	KMA	33.3	126.16	71.47	64134	38.83
**129**	KAS ch4 surface hourly AGH	AGH	49.23	19.98	1991	24643	27.16
**130**	KCO ch4 surface event NOAA	NOAA	4.97	73.47	6	89	19.26
**131**	KEY ch4 surface event NOAA	NOAA	25.67	-80.18	6	1845	18.75
**132**	KIT ch4 surface hourly ICOS	ICOS	49.09	8.42	208.75	155325	31.53
**133**	KJO ch4 surface event MPI-BGC	MPI-BGC	70.85	29.24	5	175	21.46
**134**	KMP ch4 surface hourly FMI	FMI	60.2	24.96	56	64423	44.98
**135**	KRE ch4 surface hourly ICOS	ICOS	49.58	15.08	645.47	122130	31.7
**136**	KRS ch4 surface hourly NIES	NIES	58.25	82.42	143	13974	36.26
**137**	KUM ch4 surface event NOAA	NOAA	19.53	-154.83	8.78	4857	10.77
**138**	KZD ch4 surface event NOAA	NOAA	44.31	76.07	484.82	1105	31.24
**139**	KZM ch4 surface event NOAA	NOAA	43.25	77.87	2524	1110	21.33
**140**	LAU ch4 surface event NIWA	NIWA	-45.03	169.67	380	419	8.41
**141**	LEF ch4 aircraft event NOAA	NOAA	45.96	-90.19	2124.24	4789	35.82
**142**	LEF ch4 surface event NOAA	NOAA	45.94	-90.27	842.99	4412	58.68
**143**	LEF ch4 surface hourly NOAA	NOAA	45.95	-90.27	868	235783	58.39
**144**	LEW ch4 surface event NOAA	NOAA	40.94	-76.88	261	850	50.95
**145**	LIN ch4 surface hourly ICOS	ICOS	52.17	14.12	111.43	191222	33.47
**146**	LLB ch4 surface event NOAA	NOAA	54.95	-112.45	568.25	418	88.61
**147**	LLB ch4 surface hourly EC	EC	54.95	-112.47	584.37	102918	58.11
**148**	LLN ch4 surface event NOAA	NOAA	23.47	120.87	2867	1576	22.37
**149**	LMP ch4 surface event NOAA	NOAA	35.52	12.62	50	1345	21.05

**150**	LMP ch4 surface hourly ICOS	ICOS	35.52	12.63	53	12584	22.49
**151**	LPO ch4 surface event LSCE	LSCE	48.8	-3.58	20	241	29.97
**152**	LUT ch4 surface hourly ICOS	ICOS	53.4	6.35	61	20378	61.47
**153**	MAA ch4 surface event CSIRO	CSIRO	-67.62	62.87	42	860	5
**154**	MAN ch4 aircraft event IPEN	IPEN	-2.49	-59.82	2655.44	2849	16.31
**155**	MBC ch4 surface event NOAA	NOAA	76.25	-119.35	35	54	8.38
**156**	MBO ch4 surface event NOAA	NOAA	43.98	-121.69	2742.3	1914	15.17
**157**	MDM ch4 surface event ENEA	ENEA	37.88	14.03	1653	395	21.05
**158**	MEX ch4 surface event NOAA	NOAA	18.98	-97.31	4469	967	14.18
**159**	MHD ch4 surface event LSCE	LSCE	53.33	-9.9	29.24	580	15.06
**160**	MHD ch4 surface event NOAA	NOAA	53.33	-9.9	26.1	2037	16.32
**161**	MHD ch4 surface hourly LSCE	LSCE	53.33	-9.9	29	24117	14.64
**162**	MID ch4 surface event NOAA	NOAA	28.21	-177.37	10.42	2392	11.11
**163**	MKN ch4 surface event NOAA	NOAA	-0.06	37.3	3649	309	18.33
**164**	MLO ch4 surface event NOAA	NOAA	19.53	-155.58	3419.6	5201	11.04
**165**	MLO ch4 surface hourly NOAA	NOAA	19.54	-155.58	3437	61011	11.93
**166**	MNM ch4 surface hourly JMA	JMA	24.29	153.98	27.1	168068	12.53
**167**	MQA ch4 surface event CSIRO	CSIRO	-54.48	158.97	13	923	5
**168**	MSH ch4 surface event NOAA	NOAA	41.66	-70.5	78.3	515	30.39
**169**	MVY ch4 surface event NOAA	NOAA	41.33	-70.57	16	210	26.47
**170**	MWO ch4 surface event NOAA	NOAA	34.22	-118.06	1775.23	4441	40.13
**171**	NAT ch4 surface event NOAA	NOAA	-5.64	-35.23	49.96	747	9.68
**172**	NGL ch4 surface hourly UBA	UBA	53.17	13.03	71.4	8717	32.57
**173**	NHA ch4 aircraft event NOAA	NOAA	42.92	-70.56	3159.38	4256	19.6
**174**	NMB ch4 surface event MPI-BGC	MPI-BGC	-23.58	15.03	456	282	9.15
**175**	NMB ch4 surface event NOAA	NOAA	-23.58	15.03	461	1344	10.09
**176**	NOR ch4 surface hourly ICOS	ICOS	60.09	17.48	109.2	113340	35.12
**177**	NOV ch4 aircraft event NIES	NIES	55	83	3059.76	3094	28.07
**178**	NOY ch4 surface hourly NIES	NIES	63.43	75.78	151	13575	61.96
**179**	NSK ch4 aircraft event NOAA	NOAA	58.8	-104.6	2913.52	45	11.52
**180**	NWP ch4 surface event TU	TU	-4.09	152.2	20	3600	25.23
**181**	NWR ch4 surface event NOAA	NOAA	40.05	-105.58	3526.12	6285	16.79
**182**	NYA ch4 surface event TU	TU	78.92	11.93	43	926	15.45
**183**	OBN ch4 surface event NOAA	NOAA	55.1	36.6	484	451	56.81
**184**	OHP ch4 surface hourly LSCE	LSCE	43.92	5.75	637.38	54626	27.85
**185**	OIL ch4 aircraft event NOAA	NOAA	41.28	-88.94	4228.96	424	18.43
**186**	OPE ch4 surface hourly ICOS	ICOS	48.56	5.5	450.8	124659	27.66
**187**	ORL ch4 aircraft event LSCE	LSCE	47.83	2.5	1332.2	1937	26.04
**188**	OXK ch4 surface event MPI-BGC	MPI-BGC	50.03	11.81	1022	596	24.16

**189**	OXK ch4 surface event NOAA	NOAA	50.03	11.81	1181.3	1203	32.96
**190**	OXK ch4 surface hourly ICOS	ICOS	50.03	11.81	1114.01	47602	29.45
**191**	PAL ch4 surface event NOAA	NOAA	67.97	24.12	570	1739	29.67
**192**	PAL ch4 surface hourly FMI	FMI	67.97	24.12	567	131491	27.59
**193**	PAL ch4 surface hourly ICOS	ICOS	67.97	24.12	577	33955	29.31
**194**	PCO ch4 surface event NOAA	NOAA	38.47	-28.4	2230	12	29.01
**195**	PDI ch4 surface hourly EMPA	EMPA	21.57	103.52	1478	7037	46.13
**196**	PDI ch4 surface hourly VNMHA	VNMHA	21.57	103.52	1478	40926	46.65
**197**	PDM ch4 surface event LSCE	LSCE	42.94	0.14	2879.63	422	20.83
**198**	PDM ch4 surface hourly LSCE	LSCE	42.94	0.14	2887	29118	14.16
**199**	PFA ch4 aircraft event NOAA	NOAA	64.98	-148.12	3578.54	4942	13.67
**200**	PIP ch4 aircraft event TU	TU	37.81	141.35	1911.49	2044	27.21
**201**	POC ch4 surface event NIWA	NIWA	0.99	126.45	25.85	350	18.78
**202**	POC ch4 surface event NOAA	NOAA	-1.31	-114.59	20	7298	19.11
**203**	PRS ch4 surface hourly RSE	RSE	45.94	7.71	3490	77559	21.5
**204**	PSA ch4 surface event NOAA	NOAA	-64.77	-64.05	15	2386	5.82
**205**	PTA ch4 surface event NOAA	NOAA	38.95	-123.73	22	947	28.51
**206**	PUY ch4 surface event LSCE	LSCE	45.77	2.97	1469.32	650	21.26
**207**	PUY ch4 surface hourly ICOS	ICOS	45.77	2.97	1475	42227	18.72
**208**	PUY ch4 surface hourly LSCE	LSCE	45.77	2.97	1475	49795	19.65
**209**	RBA ch4 aircraft event IPEN	IPEN	-9.2	-66.35	2320.43	2266	17.3
**210**	RGL ch4 surface hourly UNIVBRIS	UNIVBRIS	52	-2.54	273.96	73326	28.96
**211**	RPB ch4 surface event NOAA	NOAA	13.16	-59.43	20	2388	12.03
**212**	RTA ch4 aircraft event NOAA	NOAA	-21.24	-159.8	3178.36	3262	6.7
**213**	RUN ch4 surface hourly ICOS	ICOS	-21.08	55.38	2160	25300	5
**214**	RYO ch4 surface hourly JMA	JMA	39.03	141.82	280	170510	24.19
**215**	SAC ch4 surface hourly ICOS	ICOS	48.72	2.14	221.47	91926	30.05
**216**	SAN ch4 aircraft event IPEN	IPEN	-2.85	-54.96	2118.78	2387	25.27
**217**	SAN ch4 aircraft event NOAA	NOAA	-2.86	-54.86	1979.62	333	25.84
**218**	SCA ch4 aircraft event NOAA	NOAA	32.85	-79.5	4133.68	3844	17.62
**219**	SCS ch4 surface event NOAA	NOAA	11.88	110.41	20	678	24.42
**220**	SCT ch4 surface event NOAA	NOAA	33.41	-81.83	420	3334	32.62
**221**	SCT ch4 surface hourly NOAA	NOAA	33.41	-81.83	420.2	145029	29.77
**222**	SDZ ch4 surface event NOAA	NOAA	40.65	117.12	298	584	62.62
**223**	SEY ch4 surface event NOAA	NOAA	-4.68	55.53	7	2201	14.43
**224**	SGP ch4 aircraft event NOAA	NOAA	36.63	-97.51	2299.24	7722	33.52
**225**	SGP ch4 surface event NOAA	NOAA	36.67	-97.49	374	2461	57.81
**226**	SHM ch4 surface event NOAA	NOAA	52.72	174.11	28	2334	15.51

**227**	SIS ch4 surface event CSIRO	CSIRO	60.09	-1.25	33	103	16.81
**228**	SIS ch4 surface event MPI-BGC	MPI-BGC	60.09	-1.25	30	850	23.81
**229**	SMO ch4 surface event NOAA	NOAA	-14.25	-170.57	53.52	4457	7.48
**230**	SMR ch4 surface hourly ICOS	ICOS	61.85	24.29	250.18	118630	41.75
**231**	SOD ch4 surface hourly FMI	FMI	67.36	26.64	227	45822	35.74
**232**	SPO ch4 surface event NOAA	NOAA	-89.98	-24.8	2817.19	3589	5.87
**233**	SSL ch4 surface hourly UBA	UBA	47.92	7.92	1217	8273	22.36
**234**	STE ch4 surface hourly ICOS	ICOS	53.04	8.46	164.66	88652	56.81
**235**	STM ch4 surface event NOAA	NOAA	66	2	6.17	2344	19.26
**236**	STR ch4 surface event NOAA	NOAA	37.76	-122.45	486	6800	40.97
**237**	SUM ch4 surface event NOAA	NOAA	72.6	-38.42	3214.54	2136	13.43
**238**	SUR ch4 aircraft event NIES	NIES	61.2	73.2	2907.9	3062	31.41
**239**	SVB ch4 surface hourly ICOS	ICOS	64.26	19.77	324.62	105509	35.37
**240**	SVV ch4 surface hourly NIES	NIES	51.33	82.13	534.37	11085	120.75
**241**	SYO ch4 surface event NOAA	NOAA	-69	39.58	17.06	1143	6.14
**242**	TAB ch4 aircraft event IPEN	IPEN	-5.64	-69.85	2236.73	464	22.02
**243**	TAC ch4 surface event NOAA	NOAA	52.52	1.14	236	103	41.57
**244**	TAC ch4 surface hourly UNIVBRIS	UNIVBRIS	52.52	1.14	176.09	68199	35.78
**245**	TAO ch4 surface hourly EC	EC	43.66	-79.4	100	16708	47.43
**246**	TAP ch4 surface event NOAA	NOAA	36.73	126.13	21	3324	42.35
**247**	TDA ch4 aircraft event TU	TU	36.02	135.65	7084.95	2690	30.77
**248**	TEF ch4 aircraft event IPEN	IPEN	-3.48	-66.04	2172.76	646	22.7
**249**	TER ch4 surface event MGO	MGO	69.2	35.1	42	918	23.84
**250**	TGC ch4 aircraft event NOAA	NOAA	27.69	-96.75	3934.71	2984	18.64
**251**	THD ch4 aircraft event NOAA	NOAA	41.09	-124.22	3849.95	2734	17.88
**252**	THD ch4 surface event NOAA	NOAA	41.05	-124.15	112	1505	19.78
**253**	TIK ch4 surface event MGO	MGO	71.59	128.92	18	241	27.17
**254**	TIK ch4 surface event NOAA	NOAA	71.6	128.89	29	652	21.84
**255**	TIK ch4 surface hourly FMI	FMI	71.6	128.89	29	75491	21.94
**256**	TLL ch4 surface hourly DMC	DMC	-30.17	-70.8	2159	48055	15.19
**257**	TLL ch4 surface hourly EMPA	EMPA	-30.17	-70.8	2225	7406	9.79
**258**	TMD ch4 surface event NOAA	NOAA	39.58	-77.49	673.91	141	41.37
**259**	TOH ch4 surface hourly ICOS	ICOS	51.81	10.53	886.74	125927	29.23
**260**	TPD ch4 surface hourly EC	EC	42.64	-80.56	266	67411	43.03
**261**	TRM ch4 surface event LSCE	LSCE	-15.88	54.52	10	105	9.71
**262**	TRN ch4 surface hourly ICOS	ICOS	47.96	2.11	214.7	143295	29.21
**263**	TRN ch4 surface hourly LSCE	LSCE	47.96	2.11	311	27395	26.7
**264**	TTA ch4 surface hourly UOB	UOB	56.56	-2.99	622	40878	19.94

**265**	ULB ch4 aircraft event NOAA	NOAA	47.4	106.04	2713.23	552	17.43
**266**	ULD ch4 surface hourly KMA	KMA	37.48	130.9	220.9	54305	37.34
**267**	USH ch4 surface event NOAA	NOAA	-54.85	-68.31	32	1374	5
**268**	UTA ch4 surface event NOAA	NOAA	39.9	-113.72	1332	2460	21
**269**	UTO ch4 surface hourly FMI	FMI	59.78	21.37	64	43561	43.17
**270**	UTO ch4 surface hourly ICOS	ICOS	59.78	21.37	65	28781	47.65
**271**	UUM ch4 surface event NOAA	NOAA	44.45	111.1	1012	2266	27.86
**272**	VGN ch4 surface hourly NIES	NIES	54.5	62.32	277	15037	29.39
**273**	VKV ch4 surface hourly MGO	MGO	59.95	30.7	76	25870	76.57
**274**	WBI ch4 aircraft event NOAA	NOAA	41.85	-91.44	4177.05	3474	21.11
**275**	WBI ch4 surface event NOAA	NOAA	41.72	-91.35	620.6	3814	43.29
**276**	WGC ch4 aircraft event NOAA	NOAA	38.18	-119.8	901.79	165	29.07
**277**	WGC ch4 surface event NOAA	NOAA	38.26	-121.49	93.39	2933	120.92
**278**	WGC ch4 surface hourly NOAA	NOAA	38.26	-121.49	285.25	343102	89.43
**279**	WIS ch4 surface event NOAA	NOAA	30.64	34.85	401.92	2361	25.06
**280**	WKT ch4 surface event NOAA	NOAA	31.31	-97.33	617.72	4060	47.39
**281**	WLG ch4 surface event NOAA	NOAA	36.28	100.9	3823.87	2418	15.28
**282**	WPC ch4 surface event NOAA	NOAA	0.79	151.25	10	552	14.59
**283**	WSA ch4 surface event EC	EC	43.93	-60.02	30	1182	19.78
**284**	WSA ch4 surface hourly EC	EC	43.93	-60.01	30	98715	18.07
**285**	YAK ch4 surface hourly NIES	NIES	62.09	129.36	341	10654	27.94
**286**	YON ch4 surface hourly JMA	JMA	24.47	123.01	50	168060	24.97
**287**	ZEP ch4 surface event NOAA	NOAA	78.91	11.89	479	2528	16.16
**288**	ZEP ch4 surface hourly ICOS	ICOS	78.91	11.89	489	31923	16.87
**289**	ZEP ch4 surface hourly NILU	NILU	78.91	11.89	489	68159	32.58
**290**	ZGT ch4 surface hourly UBA	UBA	54.43	12.73	7.5	8360	36.14
**291**	ZSF ch4 surface hourly UBA	UBA	47.42	10.98	2670	8287	27.1
**292**	ZSF ch4 surface hourly UBAG	UBAG	47.42	10.98	2670	148212	21
**Unassimilated**							
	**Site Code**	**Laboratory**	**Mean Latitude**	**Mean Longitude**	**Mean Elevation (MASL)**	**No. of Observation**	**Model-dat Mismatch (ppb)**
1	AAO ch4 aircraft event NOAA	NOAA	40.14	-88.58	1966.82	3271	N/A
2	ABT ch4 surface hourly EC	EC	49.01	-122.34	93	51389	N/A
3	ACG ch4 aircraft event NOAA	NOAA	67.81	-154.92	3129	1765	N/A
4	ACT ch4 aircraft event NOAA	NOAA	37.52	-87.69	2195.32	1486	N/A
5	ALT ch4 surface event MPI-BGC	MPI-BGC	82.45	-62.51	185	432	N/A
6	ALT ch4 surface hourly EC	EC	82.45	-62.51	195	150696	N/A
7	AMS ch4 surface hourly LSCE	LSCE	-37.8	77.54	75	12761	N/A

**8**	AMY ch4 surface hourly KMA	KMA	36.54	126.33	47	148695	N/A
**9**	AZV ch4 surface hourly NIES	NIES	54.71	73.03	147.62	10548	N/A
**10**	BAO ch4 aircraft event NOAA	NOAA	40.05	-105.01	3094.69	12	N/A
**11**	BAO ch4 surface hourly NOAA	NOAA	40.05	-105	1719.16	4178	N/A
**12**	BCK ch4 surface hourly EC	EC	62.8	-115.92	220	76883	N/A
**13**	BIK ch4 surface event MPI-BGC	MPI-BGC	53.2	22.75	183	414	N/A
**14**	BIR ch4 surface hourly NILU	NILU	58.39	8.25	218	8317	N/A
**15**	BIS ch4 surface hourly LSCE	LSCE	44.38	-1.23	214	86947	N/A
**16**	BKT ch4 surface hourly BMKG	BMKG	-0.2	100.32	896	39862	N/A
**17**	BKT ch4 surface hourly EMPA	EMPA	-0.2	100.32	896.5	8061	N/A
**18**	BLK ch4 surface hourly EC	EC	64.33	-96.01	61	19836	N/A
**19**	BRA ch4 surface hourly EC	EC	50.2	-104.71	630	80974	N/A
**20**	BRW ch4 surface hourly NOAA	NOAA	71.32	-156.61	27.69	94835	N/A
**21**	BRZ ch4 surface hourly NIES	NIES	56.15	84.33	200.48	22219	N/A
**22**	CAR ch4 aircraft event NOAA	NOAA	40.9	-104.8	5059.95	8536	N/A
**23**	CBW ch4 surface hourly ECN	ECN	51.97	4.93	82.54	47412	N/A
**24**	CBY ch4 surface hourly EC	EC	69.13	-105.06	47	66185	N/A
**25**	CDL ch4 surface hourly EC	EC	53.99	-105.12	630	31137	N/A
**26**	CGO ch4 surface highfreq AGAGE	AGAGE	-40.68	144.69	151.92	183484	N/A
**27**	CHC ch4 surface hourly LSCE	LSCE	-16.2	-68.1	5355	49218	N/A
**28**	CHL ch4 surface hourly EC	EC	58.74	-93.82	89	63471	N/A
**29**	CHM ch4 surface hourly EC	EC	49.69	-74.34	423	23872	N/A
**30**	CHS ch4 surface hourly NOAA	NOAA	68.51	161.53	46.19	108724	N/A
**31**	CMN ch4 surface hourly IAFMS	IAFMS	44.17	10.68	2177	48861	N/A
**32**	CMN ch4 surface hourly ICOS	ICOS	44.19	10.7	2173	22346	N/A
**33**	CMN ch4 surface hourly UNIURB	UNIURB	44.17	10.68	2172	10299	N/A
**34**	COB ch4 aircraft event NOAA	NOAA	42	-88.47	3172.08	640	N/A
**35**	COI ch4 surface hourly NIES	NIES	43.16	145.5	94	8714	N/A
**36**	CPA ch4 surface hourly EMPA	EMPA	42.64	77.07	1623	30097	N/A
**37**	CPS ch4 surface hourly EC	EC	49.82	-74.98	420.99	75148	N/A
**38**	CPT ch4 surface hourly SAWS	SAWS	-34.35	18.49	260	140642	N/A
**39**	CRV ch4 aircraft event NOAA	NOAA	64.4	-149.68	1446.07	2451	N/A
**40**	CRV ch4 surface event NOAA	NOAA	64.99	-147.6	643.13	1648	N/A
**41**	CRV ch4 surface hourly NOAA	NOAA	64.99	-147.6	628.18	237517	N/A
**42**	CVO ch4 surface event MPI-BGC	MPI-BGC	16.86	-24.87	10	661	N/A
**43**	CVO ch4 surface hourly UOE	UOE	16.86	-24.87	31	53706	N/A
**44**	DEM ch4 surface hourly NIES	NIES	59.79	70.87	115.55	14115	N/A
**45**	DEU ch4 surface hourly UBA	UBA	49.77	7.05	480	8442	N/A

**46**	DWS ch4 surface hourly EC	EC	43.78	-79.47	218	90374	N/A
**47**	EGB ch4 surface hourly EC	EC	44.23	-79.78	270.55	112225	N/A
**48**	ESP ch4 surface hourly EC	EC	49.38	-126.54	47	98212	N/A
**49**	EST ch4 surface hourly EC	EC	51.67	-110.21	752.2	91009	N/A
**50**	ETL ch4 surface hourly EC	EC	54.35	-104.99	598	118445	N/A
**51**	FAM ch4 aircraft event NOAA	NOAA	52.56	0.82	715.5	22	N/A
**52**	FKL ch4 surface hourly LSCE	LSCE	35.34	25.67	165	55471	N/A
**53**	FNE ch4 surface hourly EC	EC	58.84	-122.57	376	49095	N/A
**54**	FSD ch4 surface hourly EC	EC	49.88	-81.57	250	151131	N/A
**55**	GAT ch4 surface hourly ICOS	ICOS	53.07	11.44	225.63	186927	N/A
**56**	GIF ch4 surface hourly LSCE	LSCE	48.71	2.15	167	50783	N/A
**57**	GSN ch4 surface hourly NIER	NIER	33.15	126.12	144	7576	N/A
**58**	GVN ch4 surface event MPI-BGC	MPI-BGC	-70.67	-8.27	40	165	N/A
**59**	HAA ch4 aircraft event NOAA	NOAA	21.23	-158.91	3977.4	2343	N/A
**60**	HAT ch4 surface hourly NIES	NIES	24.06	123.81	47.3	8771	N/A
**61**	HEL ch4 surface hourly ICOS	ICOS	54.18	7.88	153	1761	N/A
**62**	HIP ch4 aircraft event NOAA	NOAA	14.03	-91.36	5292.98	1384	N/A
**63**	HNP ch4 surface hourly EC	EC	43.61	-79.39	97	45698	N/A
**64**	HOW ch4 aircraft event NOAA	NOAA	42.2	-68.13	1421.03	29	N/A
**65**	HPB ch4 surface hourly DWD	DWD	47.8	11.02	1107	8640	N/A
**66**	HPB ch4 surface hourly ICOS	ICOS	47.8	11.02	1027.3	133719	N/A
**67**	HTM ch4 surface hourly ICOS	ICOS	56.1	13.42	198.4	107785	N/A
**68**	HUN ch4 surface hourly ELTE	ELTE	46.95	16.65	344	33597	N/A
**69**	IGR ch4 surface hourly NIES	NIES	63.19	64.42	42.65	15135	N/A
**70**	INU ch4 surface hourly EC	EC	68.32	-133.53	123	81970	N/A
**71**	INX ch4 aircraft event NOAA	NOAA	39.87	-86.2	1006.47	350	N/A
**72**	INX ch4 surface event NOAA	NOAA	39.82	-86.03	352.16	2297	N/A
**73**	IPR ch4 surface hourly ICOS	ICOS	45.81	8.63	290.08	91991	N/A
**74**	IPR ch4 surface hourly IES	IES	45.81	8.63	239	57160	N/A
**75**	IZO ch4 surface hourly AEMET	AEMET	28.31	-16.5	2397.55	161589	N/A
**76**	JFJ ch4 surface event MPI-BGC	MPI-BGC	46.55	7.99	3570	327	N/A
**77**	JFJ ch4 surface hourly EMPA	EMPA	46.55	7.99	3580	122515	N/A
**78**	JFJ ch4 surface hourly ICOS	ICOS	46.55	7.99	3575	36049	N/A
**79**	JGS ch4 surface hourly KMA	KMA	33.3	126.16	71.47	64134	N/A
**80**	KAS ch4 surface hourly AGH	AGH	49.23	19.98	1991	24643	N/A
**81**	KIT ch4 surface hourly ICOS	ICOS	49.09	8.42	207.44	155325	N/A
**82**	KMP ch4 surface hourly FMI	FMI	60.2	24.96	56	64423	N/A
**83**	KRE ch4 surface hourly ICOS	ICOS	49.58	15.08	645.88	122130	N/A
**84**	KRS ch4 surface hourly NIES	NIES	58.25	82.42	125.24	13974	N/A

**85**	LAC ch4 surface event NOAA	NOAA	34.07	-118.21	230	839	N/A
**86**	LAU ch4 surface hourly NIWA	NIWA	-45.04	169.68	380	1187	N/A
**87**	LEF ch4 aircraft event NOAA	NOAA	45.93	-90.27	2536.44	4789	N/A
**88**	LEF ch4 surface hourly NOAA	NOAA	45.95	-90.27	635.89	235783	N/A
**89**	LIN ch4 surface hourly ICOS	ICOS	52.17	14.12	111.04	191222	N/A
**90**	LLB ch4 surface hourly EC	EC	54.95	-112.47	584.57	102918	N/A
**91**	LMP ch4 surface event ENEA	ENEA	35.52	12.63	47	1031	N/A
**92**	LMP ch4 surface hourly ENEA	ENEA	35.52	12.63	47	1042	N/A
**93**	LMP ch4 surface hourly ICOS	ICOS	35.52	12.63	53	12584	N/A
**94**	LUT ch4 surface hourly ICOS	ICOS	53.4	6.35	61	20378	N/A
**95**	MCI ch4 aircraft event NOAA	NOAA	41.05	-89.88	1247.36	128	N/A
**96**	MHD ch4 surface highfreq AGAGE	AGAGE	53.33	-9.9	25	9765	N/A
**97**	MHD ch4 surface hourly LSCE	LSCE	53.33	-9.9	29	24117	N/A
**98**	MLO ch4 surface hourly NOAA	NOAA	19.54	-155.58	3437	61011	N/A
**99**	MNM ch4 surface hourly JMA	JMA	24.29	153.98	27.1	168068	N/A
**100**	MRC ch4 aircraft event NOAA	NOAA	41.7	-76.22	1264.79	77	N/A
**101**	MRC ch4 surface event NOAA	NOAA	41.53	-76.27	622.87	874	N/A
**102**	NEB ch4 surface event NOAA	NOAA	39.32	-76.58	110.95	82	N/A
**103**	NGL ch4 surface hourly UBA	UBA	53.17	13.03	71.4	8717	N/A
**104**	NGM ch4 surface event TU	TU	-1.85	149.81	20	629	N/A
**105**	NOR ch4 surface hourly ICOS	ICOS	60.09	17.48	109.33	113340	N/A
**106**	NOY ch4 surface hourly NIES	NIES	63.43	75.78	138.35	13575	N/A
**107**	NSA ch4 aircraft event NOAA	NOAA	69	-151.06	1467.96	378	N/A
**108**	NWB ch4 surface event NOAA	NOAA	39.34	-76.69	189.89	53	N/A
**109**	NWF ch4 surface event NOAA	NOAA	40.03	-105.43	3067.68	880	N/A
**110**	OHP ch4 surface hourly LSCE	LSCE	43.92	5.75	637.65	54626	N/A
**111**	OPE ch4 surface hourly ICOS	ICOS	48.56	5.5	449.9	124659	N/A
**112**	ORC ch4 aircraft minute NOAA	NOAA	-46.21	-72.31	7297.29	6108	N/A
**113**	OXK ch4 surface hourly ICOS	ICOS	50.03	11.81	1113.65	47602	N/A
**114**	PAL ch4 surface hourly FMI	FMI	67.97	24.12	567	131491	N/A
**115**	PAL ch4 surface hourly ICOS	ICOS	67.97	24.12	577	33955	N/A
**116**	PAO ch4 surface event NOAA	NOAA	18.23	-72.79	10	177	N/A
**117**	PDI ch4 surface hourly EMPA	EMPA	21.57	103.52	1478	7037	N/A
**118**	PDI ch4 surface hourly VNMHA	VNMHA	21.57	103.52	1478	40926	N/A
**119**	PDM ch4 surface hourly LSCE	LSCE	42.94	0.14	2887	29118	N/A
**120**	PRS ch4 surface hourly RSE	RSE	45.94	7.71	3490	77559	N/A
**121**	PUY ch4 surface hourly ICOS	ICOS	45.77	2.97	1475	42227	N/A
**122**	PUY ch4 surface hourly LSCE	LSCE	45.77	2.97	1475	49795	N/A

**123**	RGL ch4 surface hourly UNIVBRIS	UNIVBRIS	52	-2.54	275.89	73326	N/A
**124**	RPB ch4 surface highfreq AGAGE	AGAGE	13.16	-59.43	42	10858	N/A
**125**	RUN ch4 surface hourly ICOS	ICOS	-21.08	55.38	2160	25300	N/A
**126**	RYO ch4 surface hourly JMA	JMA	39.03	141.82	280	170510	N/A
**127**	S2K ch4 aircraft event NOAA	NOAA	-23.92	29.37	4337.18	172	N/A
**128**	SAC ch4 surface hourly ICOS	ICOS	48.72	2.14	221.79	91926	N/A
**129**	SCT ch4 surface hourly NOAA	NOAA	33.41	-81.83	235.25	145029	N/A
**130**	SGP ch4 aircraft event NOAA	NOAA	36.8	-97.5	4459.11	7722	N/A
**131**	SGP ch4 surface event NOAA	NOAA	36.61	-97.49	328.07	2461	N/A
**132**	SMO ch4 surface highfreq AGAGE	AGAGE	-14.25	-170.56	77	11671	N/A
**133**	SMR ch4 surface hourly ICOS	ICOS	61.85	24.29	250.56	118630	N/A
**134**	SOD ch4 surface hourly FMI	FMI	67.36	26.64	227	45822	N/A
**135**	SSL ch4 surface hourly UBA	UBA	47.92	7.92	1217	8273	N/A
**136**	SSL ch4 surface hourly UBAG	UBAG	47.9	7.92	1215.29	140689	N/A
**137**	STE ch4 surface hourly ICOS	ICOS	53.04	8.46	164.87	88652	N/A
**138**	STR ch4 surface event NOAA	NOAA	37.76	-122.45	370	6800	N/A
**139**	SVB ch4 surface hourly ICOS	ICOS	64.26	19.77	325.08	105509	N/A
**140**	SVV ch4 surface hourly NIES	NIES	51.33	82.13	534.49	11085	N/A
**141**	TAC ch4 surface hourly UNIVBRIS	UNIVBRIS	52.52	1.14	178.43	68199	N/A
**142**	TAO ch4 surface hourly EC	EC	43.66	-79.4	100	16708	N/A
**143**	THD ch4 surface highfreq AGAGE	AGAGE	41.05	-124.15	140	11287	N/A
**144**	TIK ch4 surface hourly FMI	FMI	71.6	128.89	29	75491	N/A
**145**	TLL ch4 surface hourly DMC	DMC	-30.17	-70.8	2159	48055	N/A
**146**	TLL ch4 surface hourly EMPA	EMPA	-30.17	-70.8	2225	7406	N/A
**147**	TOH ch4 surface hourly ICOS	ICOS	51.81	10.53	886.58	125927	N/A
**148**	TOM ch4 aircraft event NOAA	NOAA	11.22	-82.32	5281.65	1229	N/A
**149**	TPD ch4 surface hourly EC	EC	42.64	-80.56	266	67411	N/A
**150**	TPI ch4 surface event NOAA	NOAA	10.38	114.37	9	249	N/A
**151**	TRN ch4 surface hourly ICOS	ICOS	47.96	2.11	214.48	143295	N/A
**152**	TRN ch4 surface hourly LSCE	LSCE	47.96	2.11	311	27395	N/A
**153**	TTA ch4 surface hourly UOB	UOB	56.56	-2.99	622	40878	N/A
**154**	ULD ch4 surface hourly KMA	KMA	37.48	130.9	220.9	54305	N/A
**155**	UTO ch4 surface hourly FMI	FMI	59.78	21.37	64	43561	N/A
**156**	UTO ch4 surface hourly ICOS	ICOS	59.78	21.37	65	28781	N/A
**157**	VGN ch4 surface hourly NIES	NIES	54.5	62.32	252	15037	N/A
**158**	VKV ch4 surface hourly MGO	MGO	59.95	30.7	76	25870	N/A
**159**	VRS ch4 surface event MPI-BGC	MPI-BGC	81.58	-16.64	24	94	N/A

**160**	WGC ch4 surface hourly NOAA	NOAA	38.26	-121.49	185.76	343102	N/A
**161**	WKT ch4 surface event NOAA	NOAA	31.32	-97.32	260.26	4060	N/A
**162**	WSA ch4 surface hourly EC	EC	43.93	-60.01	30	98715	N/A
**163**	YAK ch4 surface hourly NIES	NIES	62.09	129.36	302.77	10654	N/A
**164**	YON ch4 surface hourly JMA	JMA	24.47	123.01	50	168060	N/A
**165**	ZEP ch4 surface hourly ICOS	ICOS	78.91	11.89	489	31923	N/A
**166**	ZEP ch4 surface hourly NILU	NILU	78.91	11.89	489	68159	N/A
**167**	ZGT ch4 surface hourly UBA	UBA	54.43	12.73	7.5	8360	N/A
**168**	ZOT ch4 surface event MPI-BGC	MPI-BGC	60.75	89.38	111	764	N/A
**169**	ZSF ch4 surface hourly UBA	UBA	47.42	10.98	2670	8287	N/A
**170**	ZSF ch4 surface hourly UBAG	UBAG	47.42	10.98	2670	148212	N/A

(b)

**Assimilated**							
	**Site Code**	**Laboratory**	**Mean Latitude**	**Mean Longitude**	**Mean Elevation (MASL)**	**No. of Observation**	**Model-dat Mismatch (‰)**
**1**	ALK ch4 aircraft event TU	TU	65.88	-152.16	2092.95	63	0.18
**2**	ALT ch4 surface event MPI-BGC	MPI-BGC	82.45	-62.51	185	220	0.28
**3**	ALT ch4 surface event NOAA/INSTAAR	NOAA/INSTAAR	82.45	-62.51	190	1809	0.1
**4**	AMY ch4 surface event NOAA/INSTAAR	NOAA/INSTAAR	36.54	126.33	87	335	0.08
**5**	ARH ch4 surface event NIWA	NIWA	-77.83	166.2	184	189	0.08
**6**	ASC ch4 surface event NOAA/INSTAAR	NOAA/INSTAAR	-7.97	-14.4	90	2277	0.12
**7**	AZR ch4 surface event NOAA/INSTAAR	NOAA/INSTAAR	38.76	-27.33	24	729	0.09
**8**	BAL ch4 surface event NOAA/INSTAAR	NOAA/INSTAAR	55.35	17.22	28	394	0.15
**9**	BHD ch4 surface event NIWA	NIWA	-41.41	174.87	85	311	0.08
**10**	BHD ch4 surface event NOAA/INSTAAR	NOAA/INSTAAR	-41.41	174.87	92.46	238	0.1
**11**	BIK ch4 surface event MPI-BGC	MPI-BGC	53.2	22.75	183	139	0.31
**12**	BMW ch4 surface event NOAA/INSTAAR	NOAA/INSTAAR	32.26	-64.88	51.3	2	0.15
**13**	BRW ch4 surface event NOAA/INSTAAR	NOAA/INSTAAR	71.32	-156.61	21.66	2544	0.11
**14**	BSC ch4 surface event NOAA/INSTAAR	NOAA/INSTAAR	44.18	28.66	5	20	0.09
**15**	CBA ch4 surface event NOAA/INSTAAR	NOAA/INSTAAR	55.21	-162.72	57.04	1591	0.1
**16**	CGO ch4 surface event NOAA/INSTAAR	NOAA/INSTAAR	-40.68	144.68	164	1050	0.09
**17**	CHR ch4 surface event NOAA/INSTAAR	NOAA/INSTAAR	1.7	-157.15	5	12	0.08
**18**	CIB ch4 surface event NOAA/INSTAAR	NOAA/INSTAAR	41.81	-4.93	850	2	0.1
**19**	CON ch4 aircraft event TU	TU	9.02	134.97	11131.5	414	0.18
**20**	CVO ch4 surface event MPI-BGC	MPI-BGC	16.86	-24.87	10	413	0.27
**21**	DRP ch4 surface event NOAA/INSTAAR	NOAA/INSTAAR	-57.25	-64.26	10	2	0.1
**22**	EIC ch4 surface event NOAA/INSTAAR	NOAA/INSTAAR	-27.16	-109.43	69	150	0.08

**23**	GMI ch4 surface event NOAA/INSTAAR	NOAA/INSTAAR	13.39	144.66	5	336	0.11
**24**	HSU ch4 surface event NOAA/INSTAAR	NOAA/INSTAAR	41.05	-124.58	7.6	2	0.08
**25**	INX ch4 surface event NOAA/INSTAAR	NOAA/INSTAAR	39.58	-86.42	376.73	30	0.09
**26**	JFJ ch4 surface event MPI-BGC	MPI-BGC	46.55	7.99	3570	158	0.3
**27**	KCO ch4 surface event NOAA/INSTAAR	NOAA/INSTAAR	4.97	73.47	6	4	0.08
**28**	KJO ch4 surface event MPI-BGC	MPI-BGC	70.85	29.24	5	169	0.26
**29**	KUM ch4 surface event NOAA/INSTAAR	NOAA/INSTAAR	19.54	-154.84	7.44	1431	0.12
**30**	LLB ch4 surface event NOAA/INSTAAR	NOAA/INSTAAR	54.95	-112.45	572.1	253	0.12
**31**	LLN ch4 surface event NOAA/INSTAAR	NOAA/INSTAAR	23.47	120.87	2867	2	0.1
**32**	MEX ch4 surface event NOAA/INSTAAR	NOAA/INSTAAR	18.98	-97.31	4469	655	0.14
**33**	MHD ch4 surface event NOAA/INSTAAR	NOAA/INSTAAR	53.32	-9.9	26.14	1065	0.12
**34**	MID ch4 surface event NOAA/INSTAAR	NOAA/INSTAAR	28.21	-177.38	13.78	405	0.09
**35**	MLO ch4 surface event NOAA/INSTAAR	NOAA/INSTAAR	19.53	-155.57	3419.3	2739	0.11
**36**	NMB ch4 surface event MPI-BGC	MPI-BGC	-23.58	15.03	456	244	0.26
**37**	NWR ch4 surface event NOAA/INSTAAR	NOAA/INSTAAR	40.05	-105.58	3526	1396	0.1
**38**	NYA ch4 surface event TU	TU	78.92	11.93	43	900	0.16
**39**	OXK ch4 surface event MPI-BGC	MPI-BGC	50.03	11.81	1022	82	0.31
**40**	PFA ch4 aircraft event NOAA/INSTAAR	NOAA/INSTAAR	64.85	-148.7	2981.09	1339	0.11
**41**	POC ch4 surface event NIWA	NIWA	0.71	133.13	24.17	270	0.16
**42**	PSA ch4 surface event NOAA/INSTAAR	NOAA/INSTAAR	-64.77	-64.05	15	9	0.08
**43**	SDZ ch4 surface event NOAA/INSTAAR	NOAA/INSTAAR	40.65	117.12	298	6	0.1
**44**	SGP ch4 aircraft event NOAA/INSTAAR	NOAA/INSTAAR	36.61	-97.49	2407.51	12	0.1
**45**	SIS ch4 surface event MPI-BGC	MPI-BGC	60.09	-1.25	30	447	0.29
**46**	SMO ch4 surface event NOAA/INSTAAR	NOAA/INSTAAR	-14.25	-170.57	53.35	2516	0.1
**47**	SPO ch4 surface event NOAA/INSTAAR	NOAA/INSTAAR	-89.98	-24.8	2817.21	2125	0.1
**48**	SUM ch4 surface event NOAA/INSTAAR	NOAA/INSTAAR	72.6	-38.42	3214.54	762	0.1
**49**	TAC ch4 surface event NOAA/INSTAAR	NOAA/INSTAAR	52.52	1.14	236	73	0.1
**50**	TAP ch4 surface event NOAA/INSTAAR	NOAA/INSTAAR	36.73	126.13	21	955	0.1
**51**	WGC ch4 surface event NOAA/INSTAAR	NOAA/INSTAAR	38.26	-121.49	91.1	65	0.12
**52**	WLG ch4 surface event NOAA/INSTAAR	NOAA/INSTAAR	36.29	100.9	3815	1038	0.1
**53**	WPC ch4 surface event NOAA/INSTAAR	NOAA/INSTAAR	1.42	150.92	10	222	0.1
**54**	ZEP ch4 surface event NOAA/INSTAAR	NOAA/INSTAAR	78.91	11.89	479	935	0.1
**Unassimilated**							
	**Site Code**	**Laboratory**	**Mean Latitude**	**Mean Longitude**	**Mean Elevation (MASL)**	**No. of Observation**	**Model-dat Mismatch (‰)**
**1**	ACG ch4 aircraft event NOAA/INSTAAR	NOAA/INSTAAR	68.38	-152.32	2735.44	649	N/A
**2**	ACT ch4 aircraft event NOAA/INSTAAR	NOAA/INSTAAR	37.68	-87.69	2119.47	1267	N/A
**3**	CRV ch4 aircraft event NOAA/INSTAAR	NOAA/INSTAAR	65.59	-150.75	1402.76	1722	N/A

**4**	CRV ch4 surface event NOAA/INSTAAR	NOAA/INSTAAR	64.99	-147.6	643.13	1056	N/A
**5**	CVO ch4 surface event MPI-BGC	MPI-BGC	16.86	-24.87	10	413	N/A
**6**	GVN ch4 surface event MPI-BGC	MPI-BGC	-70.67	-8.27	40	114	N/A
**7**	HIP ch4 aircraft event NOAA/INSTAAR	NOAA/INSTAAR	18.16	-94.78	4776.61	463	N/A
**8**	INX ch4 aircraft event NOAA/INSTAAR	NOAA/INSTAAR	39.86	-86.06	801.16	149	N/A
**9**	INX ch4 surface event NOAA/INSTAAR	NOAA/INSTAAR	39.77	-86.1	267.92	30	N/A
**10**	JFJ ch4 surface event MPI-BGC	MPI-BGC	46.55	7.99	3570	158	N/A
**11**	MRC ch4 aircraft event NOAA/INSTAAR	NOAA/INSTAAR	41.73	-76.26	1285.3	65	N/A
**12**	MRC ch4 surface event NOAA/INSTAAR	NOAA/INSTAAR	41.53	-76.27	623.56	704	N/A
**13**	NGM ch4 surface event TU	TU	-0.94	148.54	20	466	N/A
**14**	NSA ch4 aircraft event NOAA/INSTAAR	NOAA/INSTAAR	70.02	-151.02	1083.82	112	N/A
**15**	TOM ch4 aircraft event NOAA/INSTAAR	NOAA/INSTAAR	11.52	-79.49	5228.24	1085	N/A
**16**	VRS ch4 surface event MPI-BGC	MPI-BGC	81.58	-16.64	24	23	N/A
**17**	ZOT ch4 surface event MPI-BGC	MPI-BGC	60.75	89.38	111	527	N/A

References

1. Tans, P. P. A note on isotopic ratios and the global atmospheric methane budget. *Global Biogeochem. Cycles* **11**, 77–81 (1997).
2. Lan, X. *et al.* Improved constraints on global methane emissions and sinks using $\delta^{13}C\text{-}CH_4$. *Global Biogeochem. Cycles* (2021).
3. Thanwerdas, J., Saunois, M., Berchet, A., Pison, I. & Bousquet, P. Investigation of the renewed methane growth post-2007 with high-resolution 3-D variational inverse modeling and isotopic constraints. *Atmos. Chem. Phys.* (2024) doi:10.5194/acp-24-2129-2024.
4. Dasgupta, B. *et al.* Global methane emission estimates from a dual-isotope inversion: New constraints from $\delta D\text{-}CH_4$. *EGUsphere* 1–26 (2025) doi:10.5194/egusphere-2025-5571.
5. Basu, S. *et al.* Estimating emissions of methane consistent with atmospheric measurements of methane and $\delta^{13}C$ of methane. *Atmos. Chem. Phys.* **22**, 15351–15377 (2022).
6. Rodell, M. & Reager, J. T. Water cycle science enabled by the GRACE and GRACE-FO satellite missions. *Nat Water* **1**, 47–59 (2023).
7. Hersbach, H. *et al.* The ERA5 global reanalysis. *Quart. J. Roy. Meteor. Soc.* **146**, 1999–2049 (2020).
8. Muñoz-Sabater, J. *et al.* ERA5-Land: A state-of-the-art global reanalysis dataset for land applications. *Earth system science data* **13**, 4349–4383 (2021).
9. Zhang, Z. *et al.* Emerging role of wetland methane emissions in driving 21st century climate change. *Proc. Natl. Acad. Sci. U. S. A.* **114**, 9647–9652 (2017).
10. Williams, E. J. The comparison of regression variables. *J. R. Stat. Soc. Series B Stat. Methodol.* **21**, 396–399 (1959).
11. Diedenhofen, B. & Musch, J. Cocor: A comprehensive solution for the statistical comparison of correlations. *PLoS One* **10**, e0121945 (2015).
12. Peng, S. *et al.* Wetland emission and atmospheric sink changes explain methane growth in 2020. *Nature* **612**, 477–482 (2022).
13. Liu, G. *et al.* Trends in the seasonal amplitude of atmospheric methane. *Nature* **641**, 660–665 (2025).
14. Spivakovsky, C. M. *et al.* Three-dimensional climatological distribution of tropospheric OH: Update and evaluation. *J. Geophys. Res. D: Atmos.* **105**, 8931–8980 (2000).
15. Montzka, S. A. *et al.* Small interannual variability of global atmospheric hydroxyl. *Science* **331**, 67–69 (2011).
16. Lan, X. *et al.* Improved Constraints on Global Methane Emissions and Sinks using $\delta^{13}C\text{-}CH_4$. *Global Biogeochem. Cycles* (2021). doi:10.1029/2021gb007000.
17. Crippa, M. *et al.* GHG emissions of all world countries, Publications Office of the European Union, Luxembourg, 2023, doi:10.2760/953322, JRC134504.
18. Sherwood, O. A., Schwietzke, S., Arling, V. A. & Etiope, G. Global inventory of gas geochemistry data from fossil fuel, microbial and burning sources, version 2017. *Earth System Science Data* **9**, 639–656 (2017).
19. Sherwood, O. A., Schwietzke, S. & Lan, X. Global $\delta^{13}C\text{-}CH_4$ source signature inventory 2020. *Monitoring Laboratory Data Repository.* doi:10.15138/qn55-e011 (2021).
20. Etiope, G., Ciotoli, G., Schwietzke, S. & Schoell, M. Gridded maps of geological methane emissions and their isotopic signature. *Earth System Science Data* **11**, 1–22 (2019).
21. Van Der Werf, G. R. *et al.* Global fire emissions estimates during 1997--2016. *Earth System Science Data* **9**, 697–720 (2017).
22. Randerson, J. T., Werf, G. R. van der, Giglio, L., Collatz, G. J. & Kasibhatla., P. S. Global Fire Emissions Database, Version 4.1 (GFEDv4). *ORNL DAAC, Oak Ridge, Tennessee, USA* (2018).
23. Still, C. J., Berry, J. A., Collatz, G. J. & DeFries, R. S. Global distribution of C 3 and C 4 vegetation: Carbon cycle implications. *Global Biogeochem. Cycles* **17**, 6–1–6–14 (2003).
24. Bergamaschi, P. *et al.* Satellite chartography of atmospheric methane from SCIAMACHY on board ENVISAT: 2. Evaluation based on inverse model simulations. *J. Geophys. Res.* **112**, (2007).

25. Liu, L. *et al.* Uncertainty Quantification of Global Net Methane Emissions from Terrestrial Ecosystems Using a Mechanistically-based Biogeochemistry Model. *Journal of Geophysical Research: Biogeosciences* **125(6)**, e2019JG005428. (2020).
26. Matthews E. and Fung, I. Methane emission from natural wetlands: Global distribution, area, and environmental characteristics of sources. *Global Biogeochem. Cycles* **1**, 61–86 (1987).
27. Ganesan, A. L. *et al.* Spatially Resolved Isotopic Source Signatures of Wetland Methane Emissions. *Geophys. Res. Lett.* **45**, 3737–3745 (2018).
28. Cantrell, C. A. *et al.* Carbon kinetic isotope effect in the oxidation of methane by the hydroxyl radical. *J. Geophys. Res. D: Atmos.* **95**, 22455–22462 (1990).
29. Jöckel, P. *et al.* The atmospheric chemistry general circulation model ECHAM5/MESSy1: consistent simulation of ozone from the surface to the mesosphere. *Atmos. Chem. Phys.* **6**, 5067–5104 (2006).
30. Hossaini, R. *et al.* A global model of tropospheric chlorine chemistry: Organic versus inorganic sources and impact on methane oxidation. *J. Geophys. Res. D: Atmos.* **121**, 14–271 (2016).
31. Saueressig, G., Bergamaschi, P., Crowley, J. N., Fischer, H. & Harris, G. W. Carbon kinetic isotope effect in the reaction of CH_4 with Cl atoms. *Geophys. Res. Lett.* **22**, 1225–1228 (1995).
32. Saueressig, G. *et al.* Carbon 13 and D kinetic isotope effects in the reactions of CH4 with O (1 D) and OH: new laboratory measurements and their implications for the isotopic composition of stratospheric methane. *J. Geophys. Res. D: Atmos.* **106**, 23127–23138 (2001).
33. Zhuang, Q. *et al.* Response of global soil consumption of atmospheric methane to changes in atmospheric climate and nitrogen deposition. *Global Biogeochem. Cycles* **27**, 650–663 (2013).
34. King, S. L., Quay, P. D. & Lansdown, J. M. The $^{13}C/^{12}C$ kinetic isotope effect for soil oxidation of methane at ambient atmospheric concentrations. *J. Geophys. Res.* **94**, 18273–18277 (1989).
35. Lan, X. *et al.* Database of Methane (CH_4) Abundance and Its Stable Carbon Isotope (δ13C-CH4) Composition from Atmospheric Measurements Used Infor CarbonTracker-CH_4 2023. (2023).

Supplementary Note 1. TM5 Zoom 4DVAR User Guide

1. System Requirement

To run the TM5 Zoom 4DVAR system, the following software dependencies are required:

- Python ≥ 3.6
- UDUNITS ≥ 1.12
- HDF5 ≥ 1.14
- netCDF-C ≥ 4.9
- netCDF-Fortran ≥ 4.6
- makedepf90
- A Fortran 2003-compatible compiler (e.g., Intel, GNU)
- Accelerated linear algebra library with BLAS/LAPACK interface
- TM5 Zoom 4DVAR source code
  https://sourceforge.net/p/tm5/cy3_4dvar/ci/default/tree/

Because TM5 uses OpenMP parallelization and heavy input data, execution on a high-performance computing (HPC) system is required.

2. Installation

Clone the TM5 Zoom 4DVAR repository:

hg clone http://hg.code.sf.net/p/tm5/cy3_4dvar tm5-cy3_4dvar

Typical download time is <10 minutes.

3. Demonstration Workflow

3.1 Input Data

The TM5 Zoom 4DVAR methane-isotope configuration requires the following inputs:

Emissions and source signatures

- Microbial, fossil, and pyrogenic methane sources
- See manuscript and Supplementary Information (SI) for details

Sink processes

- Chemical loss via OH, Cl, and $O(^1D)$
- Soil methane uptake
- See manuscript and SI for configuration details

Atmospheric observations

- In situ CH_4 and $\delta^{13}C$-CH_4 measurements
- Public archive:
  https://doi.org/10.15138/T4MZ-2Z29

Meteorology

- ERA5 meteorological fields
- See manuscript and SI for details

3.2 Demonstration of one-year forward simulation (Year 2000)

a) **Prepare a main .rc configuration file**
   Example:
   https://sourceforge.net/p/tm5/cy3_4dvar/ci/default/tree/user/nasa/rc/ids/glb3x2-ch4c13-inversion-full-cantrell.rc
   Note: This file links to multiple dependent .rc files. Ensure all referenced files are accessible.

b) **Run a forward simulation using the system-specific wrapper script:**

- Example: ./fwd-SYSTEM.py --start 2000 1 1 --end 2001 1 1 --walltime 480 --setuptime 120 --split Y initiate ../rc/ids/glb3x2-ch4c13-inversion-full-cantrell.rc
- Example wrapper scripts are available under:

https://sourceforge.net/p/tm5/cy3_4dvar/ci/default/tree/user/nasa/scripts/

These wrapper scripts are system specific. Some scripts (and associated rc files) already exist in the source tree. For a new system, several new rc files need to be written (e.g., machine_SYSTEM.rc, folders_SYSTEM.rc) and new scripts need to be written.

3.3 Demonstration of one-year inversion simulation (Year 2000)

a) **Prepare a main .rc configuration file**
Example:
https://sourceforge.net/p/tm5/cy3_4dvar/ci/default/tree/user/nasa/rc/ids/glb3x2-ch4c13-inversion-full-cantrell.rc
Note: This file links to multiple dependent .rc files. Ensure all referenced files are accessible.

b) Run the 4DVAR inversion:

- Example: ./var4d-SYSTEM.py --start 2000 1 1 --end 2001 1 1 --walltime 480 --setuptime 120 --split Y initiate ../rc/ids/glb3x2-ch4c13-inversion-full-cantrell.rc
- Example wrapper scripts are available under:
  https://sourceforge.net/p/tm5/cy3_4dvar/ci/default/tree/user/nasa/scripts/

These wrapper scripts are system specific. Some scripts (and associated rc files) already exist in the source tree. For a new system, several new rc files need to be written (e.g., machine_SYSTEM.rc, folders_SYSTEM.rc) and new scripts need to be written.

4. Key Configuration Files

The following configuration files are critical when running the methane–isotope system with our input datasets:

- Directory:
  https://sourceforge.net/p/tm5/cy3_4dvar/ci/default/tree/user/nasa/rc/include/
- *Operating system setup*
  Example: jobs_ch4.rc
- *Observation setup*
  Example: ch4c13_obs_parameters.rc
- *Prior emissions, source signatures, and sink setup*
  Example: ch4c13_emission_parameters.rc
- *Prior errors and sink isotope fractionation setup*
  Example: ch4c13_parameters.rc

For more information about the TM5 Zoom 4DVAR system, please reach out to Dr. Sourish Basu at sourish@umd.edu.
