# Supplementary material for: Isotopic constraints in methane inversions reveal larger trends in wetland emissions with improved linkage to terrestrial water storage
Source: Nat Commun. 2026 Jun 29;17:8112. doi: 10.1038/s41467-026-74777-4 (PMC13458832; doi:10.1038/s41467-026-74777-4)
Supplement: Supplementary file 3 [file 41467_2026_74777_MOESM3_ESM.pdf]

nature portfolio

Peer Review File

Isotopic constraints in methane inversions reveal larger trends in wetland emissions with improved linkage to terrestrial water storage

Corresponding Author: Dr Youmi Oh

Parts of this Peer Review File have been redacted as indicated to remove third-party material.

This file contains all reviewer reports in order by version, followed by all author rebuttals in order by version.

Attachments originally included by the reviewers as part of their assessment can be found at the end of this file.

Version 0:

Reviewer comments:

Reviewer #1

(Remarks to the Author)
General Comment
This manuscript presents a comprehensive and scientifically valuable study that integrates isotopic constraints into atmospheric methane inversion modeling to separate microbial, fossil, and pyrogenic CH_4 sources over the past two decades. The work is characterized by a substantial observational dataset, robust methodological design, and clear articulation of results. The use of ^{13}C-CH_4 data to improve source attribution, particularly in relation to wetland emissions and their link to terrestrial water storage, is innovative and contributes significantly to our understanding of natural versus anthropogenic CH_4 drivers. The data analysis appears sound, though I note that inversion modeling is not my primary area of expertise. Specific comments are shown below.

L63-66
I am curious about the rationale for selecting the time period 2000-2022. Why was the year 2000 chosen as the starting point?

L69-73
This data is impressive, but you seem not to have cited references for it.

L114-117
Excellent work! Standardizing the data significantly enhances the credibility of the results. Furthermore, your selection of sites in Fig S5 is very scientific, providing nearly uniform global coverage.

L148-150
I do not follow the logic here. Why is the increase in fossil fuels over temperate Eurasia attributed to coal consumption in China? Temperate Eurasia encompasses more countries than just China; this attribution is perplexing.

L157-158
Similar to the previous comment. I do not understand the reasoning behind your speculated cause. The speculation here seems somewhat tenuous.

L171
What sensitivity test are you referring to? Please specify.

L177-179
This is a very interesting result!

L199-201
I agree with this viewpoint; isotopes are excellent for source tracing! This is a strong aspect of the experimental design.

L207
Why is the period 2003-2022 used here? Your previous analysis appears to cover 2000-2022. What is the reason for changing the time interval for the correlation study?

L227-231
What do you mean by "statistically improved correlations"? In my understanding, demonstrating a statistically significant difference between multiple R^2 values from different methods (e.g., across groups) would be required to claim a statistical improvement. Simply observing an increase in R^2 at a single location does not, in my opinion, constitute a statistical improvement. I welcome your explanation if my understanding is incorrect.

L251
While I understand the term "bottom-up estimates" as it is a standard technical term, it may be unfamiliar to some readers.

L255-261
In my view, the Conclusion section does not need to be this detailed. You should focus on summarizing the most important and overarching results or viewpoints as your conclusions.

(Remarks on code availability)

Reviewer #2

(Remarks to the Author)
I co-reviewed this manuscript with one of the reviewers who provided the listed reports. This is part of the Nature Communications initiative to facilitate training in peer review and to provide appropriate recognition for Early Career Researchers who co-review manuscripts.

(Remarks on code availability)

Reviewer #3

(Remarks to the Author)
I co-reviewed this manuscript with one of the reviewers who provided the listed reports. This is part of the Nature Communications initiative to facilitate training in peer review and to provide appropriate recognition for Early Career Researchers who co-review manuscripts.

(Remarks on code availability)

Reviewer #4

(Remarks to the Author)
The manuscript by Oh and coauthors presents a global-scale evaluation of the trends and broad grouped sources of methane emissions in the past two decades. It is an important paper, with novel contributions linked to the incorporation of stable isotope observations into their models. The authors provide a higher resolution assessment of the underlying causes of recent trends, identifying a greater contribution of global wetlands to increasing CH_4 emissions. My expertise is restricted to methane cycling in wetlands and aquatic ecosystems, and applications of stable isotope methods, so I am not qualified to assess some aspects of this manuscript (e.g., the nuts and bolts of the atmospheric inversion model techniques), though for the aspects for which I am comfortable, I found the methods used and associated interpretations to be strong. The paper is clearly written.

My only major critique is the organization of the paper. The authors obviously submitted this to a journal with limited word count, and then resubmitted here without revising the structure prior to resubmission. For this reason, I found the flow of the text and placement of some key information hard to follow. My major critiques are therefore related to the organization of the paper. At this journal, you have the luxury of a larger word count, and this should be leveraged to bring in more key materials from the supporting information. This would do the reader a major service.

Line by line comments:

L63. In the supp figure and tables cited in the first few paragraphs, what is original and what is previously published here? It is written throughout as if this is all original material, so why is it placed in the introduction, without the proper context you would add if it were in the results section? These issues make the introduction harder to follow, because the source (and underlying methods) liked to these results is unclear.

Also related to this info – in fig S2, the soil is brown, but the caption says pink. Did I miss something, or is this a mistake?

L64. The three different periods are unclear from figure 1A. Add lines above in the figure or something to make it more obvious.

L72. Conclusion sentence to first pgph?

L105. “extracted from shale gas reserves” – reference needed for statement.

L108-111. This is methods info that pops up out of nowhere, before the study aims introduced.

L114. Fig S4 would be nice to see in main paper.

L120. What is this online method, why not methods section proper?

L121-126. I assume these are outputs plotted in figure 1/ If so, reference them. It is weird to see them defined so late and after figure 1 was introduced. Consider reorganizing this.

L130. Fig S8 was not referenced to in proper sequence (i.e., skipped from S7 to S9).

L131-139. Figure S9-10 referred to here, but very complicated and not well explained. Why plot latitudinally and why so complicated, without a detailed explanation?Explain the latitudinal trend. Assimilated vs. unassimilated not defined/explained.

L159. Is this fossil and pyrogenic? Fig 3m shows Asian fossil emissions highest, so interpretation as not totally clear to me.

L165. And N. America? High trend in fig 3n and 3o.

L176. Fig. S15 caption. Define K, E, too.

L187. The term decorrelation is unclear to me. How does the correlation analysis show what you are stating? Either I have confused myself, or you need another sentence in this section to expand your explanation.

L209-211. “fractional contribution of wetland emissions”… I am a bit lost here. Where did this info come from? A half sentence more would help. Reference?

L229. The only region with a sign inversion between methods is Hudson Bay. This was intriguing. Worth adding a sentence to note it and explain why.

L257. “A potential changes in” – grammar.

L278. Much of this material needs to be in the main text methods section.

(Remarks on code availability)

Version 1:

Reviewer comments:

Reviewer #1

(Remarks to the Author)
The authors well addressed all my concerns. I have no more suggestions.

(Remarks on code availability)

Reviewer #2

(Remarks to the Author)
I co-reviewed this manuscript with one of the reviewers who provided the listed reports. This is part of the Nature Communications initiative to facilitate training in peer review and to provide appropriate recognition for Early Career Researchers who co-review manuscripts.

(Remarks on code availability)

Reviewer #3

(Remarks to the Author)
My concerns have been adequately addressed by the authors, and in my judgement publication is warranted at this time.

(Remarks on code availability)

Open Access This Peer Review File is licensed under a Creative Commons Attribution 4.0 International License, which permits use, sharing, adaptation, distribution and reproduction in any medium or format, as long as you give appropriate credit to the original author(s) and the source, provide a link to the Creative Commons license, and indicate if changes were made.
In cases where reviewers are anonymous, credit should be given to 'Anonymous Referee' and the source.
The images or other third party material in this Peer Review File are included in the article's Creative Commons license, unless indicated otherwise in a credit line to the material. If material is not included in the article's Creative Commons license and your intended use is not permitted by statutory regulation or exceeds the permitted use, you will need to obtain permission directly from the copyright holder.
To view a copy of this license, visit https://creativecommons.org/licenses/by/4.0/

[Point-By-Point Response to the Reviewers' Comments]

Reviewer #1 (Remarks to the Author):

General Comment

This manuscript presents a comprehensive and scientifically valuable study that integrates isotopic constraints into atmospheric methane inversion modeling to separate microbial, fossil, and pyrogenic CH_4 sources over the past two decades. The work is characterized by a substantial observational dataset, robust methodological design, and clear articulation of results. The use of ^{13}C-CH_4 data to improve source attribution, particularly in relation to wetland emissions and their link to terrestrial water storage, is innovative and contributes significantly to our understanding of natural versus anthropogenic CH_4 drivers. The data analysis appears sound, though I note that inversion modeling is not my primary area of expertise. Specific comments are shown below.

We thank the reviewer for their thoughtful and positive assessment of our manuscript. We appreciate the recognition of the novelty and significance of integrating isotopic constraints into 3-D atmospheric methane inversions, particularly for improving source attribution and understanding wetland contributions linked to terrestrial water storage. We are also grateful for the reviewer's acknowledgement of the extensive observational dataset and robust methodological framework. We carefully address the specific comments below and have revised the manuscript accordingly to improve clarity and strengthen the presentation of our results.

L63-66

I am curious about the rationale for selecting the time period 2000-2022. Why was the year 2000 chosen as the starting point?

We selected this time period because there aren't many measurements of $\delta^{13}C$-CH_4 available before 2000, although we did have a spin-up year (1999). In case other readers have the same question, we have added mention of this in Line 119: This time period after 2000 was selected due to the availability of $\delta^{13}C$-CH_4 measurements.

L69-73

This data is impressive, but you seem not to have cited references for it.

We added a reference (Saunois et al. 2025) and revised the text based on Reviewer 1 and 4's comments on not presenting the results in the introduction part (Lines 71-75).

L114-117

Excellent work! Standardizing the data significantly enhances the credibility of the results. Furthermore, your selection of sites in Fig S5 is very scientific, providing nearly uniform global coverage.

Thank you for this comment. We moved details on our observation setup from SI to the main text in Lines 362-388 and added Table S6 to provide more detailed information on each site.

L148-150
I do not follow the logic here. Why is the increase in fossil fuels over temperate Eurasia attributed to coal consumption in China? Temperate Eurasia encompasses more countries than just China; this attribution is perplexing.

We thank the reviewer for pointing out this confusion and have revised the text as described below to add more information and qualified our statements (Lines 166-171). Because the in situ methane network is sparse, we do not have the ability to distinguish between emissions from China vs. Central Asia.

but fossil fuel emissions increased mainly in Temperate Eurasia (8±3 Tg yr^{-1}) (Fig. 3l), likely driven partly by increased coal production and use in China[35]. Coal production in China tripled between 2000 and 2013 and supplied 70% of China's energy needs international energy agency[36,37]. Despite significant investments in non-carbon energy sources, China's total coal supply has increased by 268% since 2000[36,37]. Oil and gas production also rose during this period in parts of Central Asia, and although these emissions are estimated to be smaller than those from Chinese coal production, they could also contribute to our estimated trend[36].

L157-158
Similar to the previous comment. I do not understand the reasoning behind your speculated cause. The speculation here seems somewhat tenuous.

We agree with the reviewer. We hope the following text improves the discussion about microbial emissions from the Tropics (Lines 178-184).

Given that regions such as South America and Africa host extensive wetlands alongside growing populations, it is plausible that a combination of natural and anthropogenic processes has led to increases in tropical microbial emissions, consistent with previous studies[6,9]. During 2015-2016, biomass burning emissions increased by ~10 Tg yr^{-1} due to large fires in mostly tropical Asia[38,39]. Our inverse model results are broadly consistent with independent estimates of fire emissions during 2015-2016 from tropical Asia[38,39], and studies emphasizing the dominant role of tropical wetlands[15–17].

L171
What sensitivity test are you referring to? Please specify.

This sensitivity referred to our 'Joint-OH' scenario that incorporated long-term changes in tropospheric OH (Fig. S7). To incorporate your and other reviewers' comments on more detail of

the Joint-OH scenario, we moved the majority of the section on the ‘Potential Role of Long-Term Trends in OH’ to the main text (Lines 198-214).

L177-179
This is a very interesting result!

Thank you for this comment.

L199-201
I agree with this viewpoint; isotopes are excellent for source tracing! This is a strong aspect of the experimental design.

Thank you for this comment.

L207
Why is the period 2003-2022 used here? Your previous analysis appears to cover 2000-2022. What is the reason for changing the time interval for the correlation study?

We chose this period because of the availability of the GRACE terrestrial water storage data (added Lines 242-243). GRACE was launched in 2002, and 2003 was the first full year of data. This period covers some of the low-growth period of atmospheric methane while also capturing the resumed growth that started in 2006 and accelerated in more recent years.

L227-231
What do you mean by "statistically improved correlations"? In my understanding, demonstrating a statistically significant difference between multiple R^2 values from different methods (e.g., across groups) would be required to claim a statistical improvement. Simply observing an increase in R^2 at a single location does not, in my opinion, constitute a statistical improvement. I welcome your explanation if my understanding is incorrect.

We addressed this comment by adding the Cocor framework and Williams’ test to statistically verify if the correlation is improved by adding isotope information (Lines 271-273 and Figure S23).

L251
While I understand the term "bottom-up estimates" as it is a standard technical term, it may be unfamiliar to some readers.

We eliminated the use of “bottom-up” to avoid confusion and just refer to “inventories” throughout the manuscript.

L255-261
In my view, the Conclusion section does not need to be this detailed. You should focus on summarizing the most important and overarching results or viewpoints as your conclusions.

Thank you for the comment. Following your advice, we significantly shortened conclusion section (Lines 286-319).

Reviewer #2 and 3 (Remarks to the Author):

I co-reviewed this manuscript with one of the reviewers who provided the listed reports. This is part of the Nature Communications initiative to facilitate training in peer review and to provide appropriate recognition for Early Career Researchers who co-review manuscripts.

The presented manuscript, entitled "Isotopic constraints in methane inversions reveal larger wetland emissions with improved linkage to terrestrial water storage", integrates isotopic constraints into an updated global atmospheric methane inversion system – CarbonTracker-CH4 to better partition global CH_4 source types and to track their temporal dynamics. The authors validated inversions with/without stable carbon isotope ratio ($\delta^{13}C–CH_4$) using a harmonized database of global atmospheric CH_4 and $\delta^{13}C–CH_4$ measurements. They found that the inclusion of isotopic information significantly improved constraints on microbial versus fossil CH_4 sources and uncovered the geographic distribution of increases in CH_4 sources. The study shows that wetland emissions are larger than previously estimated. However, the correlation between regional wetland CH4 emissions and terrestrial water storage (TWS) varies within major wetlands. The manuscript is scientifically sound and clearly presented. Clarifications are needed regarding the impacts of wetland priors and isotopic attribution on inversions. With these adjustments, the study will represent a valuable and influential contribution to Nature Communications and to the broader atmospheric and biogeochemical science community.

We thank the reviewers for their valuable and helpful comments. We have thoroughly addressed your major points by (1) running additional inversion scenarios using alternative wetland priors with dynamic inundation and (2) expanding the discussion of isotopic attribution in the inversion framework. Further details are provided in the point-by-point responses below.

Major comments:

1, The $\delta^{13}C–CH_4$ were mostly measured at North America and Europe sites. How would the sparse $\delta^{13}C–CH_4$ measurements in continental South America, Africa, and Australia affect the spatial attribution of microbial and fossil sources in those regions? Lines 187-189 and Fig. S18 tried to address this question partially at the continental scale. The joint scenario simulated continued decreasing fossil emissions and relatively stable pyrogenic emissions in Africa. The simulated fossil emissions in the joint scenarios are higher than in the CH4-only scenario in South America and Australia. Would you suggest more stable carbon isotope sites in these continents?

$\delta^{13}C–CH_4$ measurements remain relatively sparse over continental regions over the tropics in particular (Fig. S5). This reflects both the logistical challenges of maintaining isotope monitoring

at remote sites as well as the fundamental difficulty of consistently measuring δ^{13}C–CH_4 which requires NOAA to make these measurements only on a fraction of its global network. Nonetheless, within 30°S - 30°N, we include 30 sites operated by NOAA, MPI-BGC, TU, and NIWA that provide regular, high-precision δ^{13}C–CH_4 observations (Table S6). Collectively, these tropical and sub-tropical sites contribute approximately 15,000 in-situ surface measurements between 2000 and 2022, about one-third of the total δ^{13}C–CH_4 dataset used here. Thus, despite uneven spatial coverage, the δ^{13}C–CH_4 dataset we use provides substantial information for source attribution in the tropics, particularly for **long-term trends**. This is reflected in our correlation analyses with terrestrial water storage: regardless of wetland priors, isotopic inversions yield larger wetland emission trends and stronger correlations with terrestrial water storage (Figs. 4 and S19–23).

We further acknowledge that the correlation analysis shown in Fig. S18 (now Fig. S17) could be interpreted as indicating limited isotopic benefit for South America, Africa, and Australia. However, this metric is based on **annual-mean emissions** and is not intended to assess attribution of long-term trends (Fig. 4). Specifically, we evaluated posterior correlations between microbial and fossil sources using annual-mean emission changes from our 100-member ensemble of independent inversions. Because uncertainties in annual-mean emissions are very small (Fig. S17a), correlations that vary within these uncertainties can yield only modest apparent improvements in correlation.

To address this comment in the manuscript, we added additional explanation of the correlation analysis and its interpretation in Supplementary Information and emphasized the need for expanded isotope measurements, especially in the tropics and Arctic, in Lines 309-310.

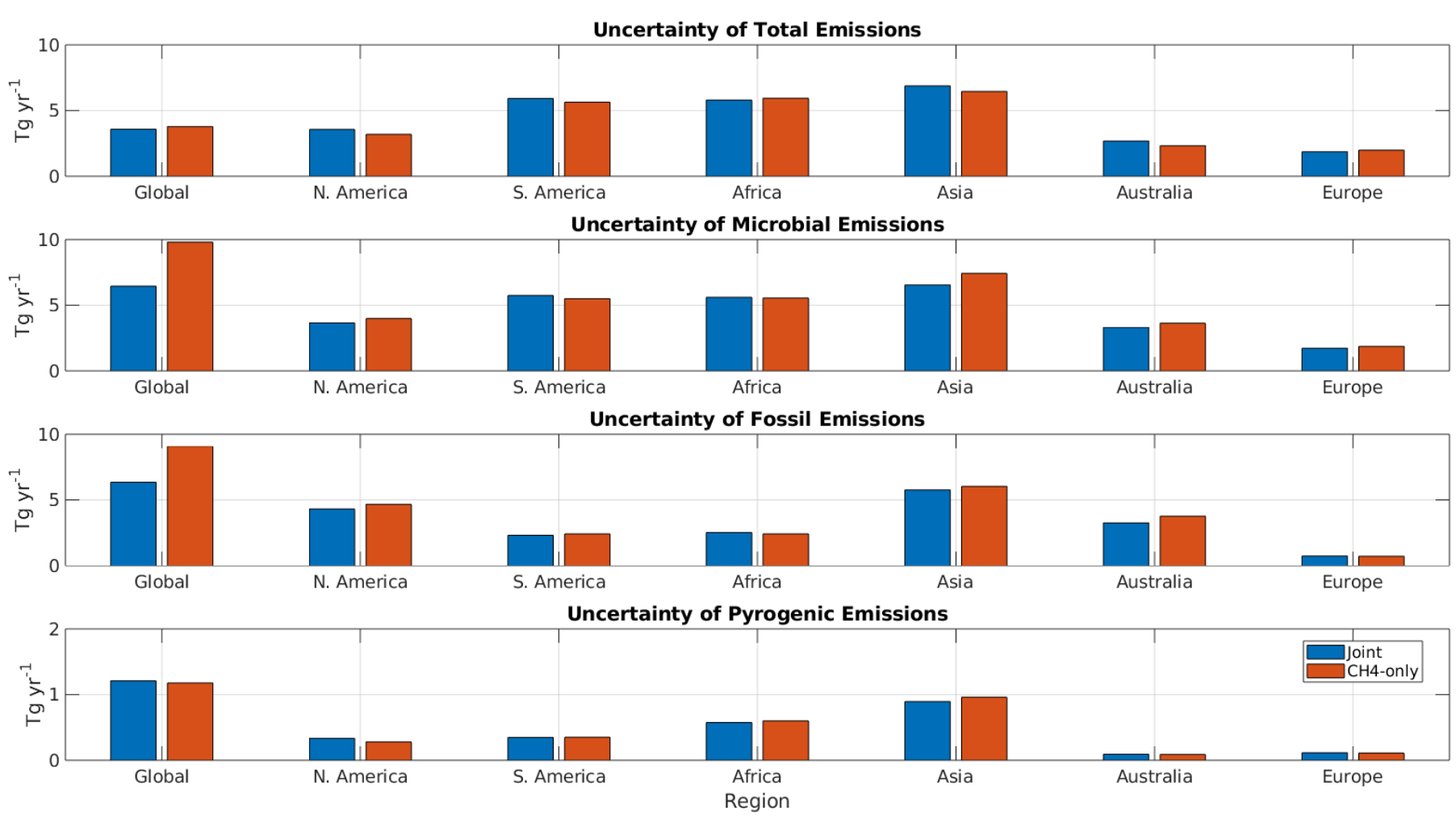

Figure S17a. Uncertainties in total, microbial, fossil, and pyrogenic emissions estimated from 100 ensemble simulations.

Also in the supporting materials line 171-172, you "forced the assimilation to closely match remote marine background sites, while some sites were given a very large model-data mismatch because they are likely influenced by strong local sources". It could be helpful to name the sites (or some example sites) that were not used in assimilation.

We apologize for the misleading wording in the original text. We did not "force" the inversion to closely match only a subset of observations; rather, remote marine background sites **exhibit** weak local gradients, which leads to smaller prior standard deviations and consequently smaller prescribed model-data mismatch values in the R matrix (Eq. 4). This model-data mismatch method is widely used and is commonly referred to as the "residual method" (Chen et al., 2022; Heald et al., 2004). To improve transparency, we added Table S6 listing all sites used for assimilation and validation, along with their corresponding model–data mismatch values for both CH_4 and $\delta^{13}C$–CH_4.

Chen, Z., Jacob, D. J., Nesser, H., Sulprizio, M. P., Lorente, A., Varon, D. J., Lu, X., Shen, L., Qu, Z., Penn, E., & Yu, X. (2022). Methane emissions from China: a high-resolution inversion of TROPOMI satellite observations. *Atmospheric Chemistry and Physics*, *22*(16), 10809–10826.

Heald, C. L., Jacob, D. J., Jones, D. B. A., Palmer, P. I., Logan, J. A., Streets, D. G., Sachse, G. W., Gille, J. C., Hoffman, R. N., & Nehrkorn, T. (2004). Comparative inverse analysis of satellite (MOPITT) and aircraft (TRACE-P) observations to estimate Asian sources of carbon monoxide: COMPARATIVE INVERSE ANALYSIS. *Journal of Geophysical Research*, *109*(D23). https://doi.org/10.1029/2004jd005185

2, How will the wetland emission priors affect the spatial pattern of microbial emissions in assimulations? The prior wetland fluxes are derived from a single process model (TEM) with a static wetland distribution. Please discuss potential biases from static wetland area assumptions and whether results change when dynamic wetland datasets are used, especially when linking wetland emissions with TWS.

To more fully evaluate the effects of alternative wetland priors, we conducted additional atmospheric inversions using wetland emissions from the **process-based LPJ-wsl model with dynamic inundation** for both the Joint and CH_4-only scenarios. These new experiments show that the CH_4-only scenario is more sensitive to wetland prior assumptions when estimating total wetland emissions than the Joint scenario, emphasizing the value of $\delta^{13}C$–CH_4 observations as constraints on fluxes. Moreover, regardless of the wetland prior used, the Joint scenarios that assimilate isotopic constraints consistently simulates larger increasing trends in wetland emissions and stronger correlations with terrestrial water storage. We incorporated these new analyses into the section "Linking Changes in Wetland CH_4 Emissions to Environmental Changes with Isotopic Constraints" We have also added an additional panel to Fig. 4 (e) showing correlations when using LPJ-wsl as a prior. (Lines 237-284; Figs. 4 and S19-23).

In Southeast US (region 3) and South America (region 5), the emission trends are stronger in the joint scenario than in the CH4-only scenario, whereas the correlations with TWS are opposite. Please explain other possible environmental factors that could drive the stronger trends in these regions and their implications.

The fact that R^2 values for the southeast US are ~0.5 ($p<0.05$) and only ~0.1 for South America ($p>>0.05$) certainly indicates that other factors are at play in driving wetland emissions trends. However, the most important point to recognize here (also see Fig. S19 c,e) is that a) there is

essentially no, or low, correlation for South America, and b) the trends between Joint and CH_4-only are not statistically different at one sigma (Fig 4c), which led slightly higher correlation for the Joint scenario using alternative wetland priors.

Minor comments:

1, Line 94-95, are there limitations or differences in the estimation of global and regional CH4 emissions in the referenced studies due to a lack of observational constraints on the stable isotope ratio? Explaining such limitations or differences, if any, can help the audience understand the reason for including $\delta^{13}C–CH_4$ constraints in source partitioning.
Following reviewer's comment, we added Lines 99-101: This lack of isotopic constraints may lead to biased source partitioning and a higher sensitivity to prior assumptions when attributing changes among different sources.

2, Line 96-111, this paragraph makes a strong point of utilizing isotopic information in the presented inversion framework with minor impacts of uncertainties in isotopic source signatures.

Thank you for your comment.

3, Line 124-125, the three experimental scenarios substantially support the argument of this manuscript. Were the changes in the tropospheric OH scaling factor applied universally to all grids? Please clarify how the scaling factor was applied spatially.

Yes, the OH scaling factor for a given year (Fig. S7) was applied to the entire troposphere (all grid cells in three dimensions), and the text is revised accordingly in Line 131. Because of this globally uniform approach, we retained the Spivakovsky et al seasonal and spatial patterns of OH and did not test the sensitivity of the LMDz-INCA OH spatial patterns, only their global mean annual variability. The universally applied OH factor could cause uncertainty in our assumptions; however, one key result brought out in our revised manuscript is that the changed OH in the Joint-OH scenario only significantly impacts the total source, and thus total sink (to match CH4 observations) and has only a marginal impact on the isotope-based partitioning of fossil and microbial fluxes (as normalized by total fluxes) (Lines 198-214).

4, Line 137-139, What is the regional shift? Please provide more descriptions of Fig. S10 and explicitly explain the regional shift. Fig. S10 shows CH4-only inversion underestimates $\delta^{13}CCH_4$, and the underestimation intensifies in the Tropics and Polar Southern Hemisphere after 2015. Do you mean the shift is needed in these two regions?

Since the CH_4-only scenario does not match the latitudinal gradients of isotope data, regional shift (ex. Move more microbial emissions from temperate to tropical regions) is needed to match the observed regional changes in methane isotope values (Fig. S11). We added more details on the model-data comparison part to address your and Reviewer 4's comments (Lines 139-154).

5, Line 174-176, please provide a few sentences summarizing the changes in the spatial distribution of fossil and microbial sources in the Joint+OH scenario.

To incorporate all reviewers' comments on more details of the Joint-OH scenario, we moved the section on the 'Potential Role of Long-Term Trends in OH' to the main text and added more details (Lines 198-214).

6. Line 413-415, Figure 1 seems to show monthly data between 2000 and 2021, not 2022. Did I interpret it wrong? Also in the supporting materials line 196-199, you described eight 5-year inversions with only the first and last year overlapping between periods. However, the interval between the starting years is only four years (including the start and end years). If the interval were 5 years as described, then there would be 4 years of overlap (first two years and last two years). Please correct or clarify it.

Thanks for the correction. We revised Figure 1 to add 2022.
The interval between the starting years is 3 years in fact, as we are not using the first and last year of the simulation for spin-up and spin-down periods (Lines 398-401). Please find Figure R2 below.

[REDACTED]

Figure R2. A schematic of the time splitting of our inversions (Figure 2a of Basu et al. (2022)).

7, Supporting materials line 122-123, "larger emissions increases occurring in the tropics", Asian tropics?

It increased throughout the tropical zone, where the OH concentrations are high (Fig. S7b).

Reviewer #4 (Remarks to the Author):

The manuscript by Oh and coauthors presents a global-scale evaluation of the trends and broad grouped sources of methane emissions in the past two decades. It is an important paper, with novel contributions linked to the incorporation of stable isotope observations into their models. The authors provide a higher resolution assessment of the underlying causes of recent trends, identifying a greater contribution of global wetlands to increasing CH4 emissions. My expertise is restricted to methane cycling in wetlands and aquatic ecosystems, and applications of stable isotope methods, so I am not qualified to assess some aspects of this manuscript (e.g., the nuts and bolts of the atmospheric inversion model techniques), though for the aspects for which I am comfortable, I found the methods used and associated interpretations to be strong. The paper is clearly written.

My only major critique is the organization of the paper. The authors obviously submitted this to a journal with limited word count, and then resubmitted here without revising the structure prior to resubmission. For this reason, I found the flow of the text and placement of some key information hard to follow. My major critiques are therefore related to the organization of the paper. At this journal, you have the luxury of a larger word count, and this should be leveraged to bring in more key materials from the supporting information. This would do the reader a major service.

We thank Reviewer #4 for their thoughtful evaluation of the manuscript. To address the concerns regarding manuscript organization and flow, we substantially revised the structure of the paper by moving much of the methodological detail and supporting analyses from the Supplementary Information into the main text. As a result, the main-text length increased from approximately 2,900 to 5,000 words. We also added clarifying material throughout the manuscript to improve narrative cohesion and to help readers more easily follow the logic of the analyses and results.

Line by line comments:

L63. In the supp figure and tables cited in the first few paragraphs, what is original and what is previously published here? It is written throughout as if this is all original material, so why is it placed in the introduction, without the proper context you would add if it were in the results section? These issues make the introduction harder to follow, because the source (and underlying methods) liked to these results is unclear.

Tables S1 and S2 were all dealt with previously published material and are referenced correctly. We realize that the source of Figure S1 was confusing, so thanks for pointing this out. The Fig. S1 caption now reads

(a) Pie chart of global mean CH_4 emissions from anthropogenic and natural microbial, fossil, and pyrogenic source sectors based on the results from the "Joint" inversion presented in this study.

Although these results have not yet been published in any peer-reviewed journal, they are already available, including in Fig. S1 on the CarbonTracker-CH_4 website (carbontracker.noaa.gov). CarbonTracker-CH_4 is a routinely released product from NOAA's Global Monitoring Laboratory, updated annually.

Also related to this info – in fig S2, the soil is brown, but the caption says pink. Did I miss something, or is this a mistake?

Thank you for the correction. The caption and figure in Fig. S2 are revised.

L64. The three different periods are unclear from figure 1A. Add lines above in the figure or something to make it more obvious.

Yes, we added shaded regions above the figure in Figure 1a.

L72. Conclusion sentence to first pgph?

We removed the exact portion of different source sectors and cited previously published papers on the general sources and sinks of the global methane budget (Lines 71-75).

L105. “extracted from shale gas reserves” – reference needed for statement.

We added the reference accordingly (Riddell-Young et al., PNAS, 2026).

L108-111. This is methods info that pops up out of nowhere, before the study aims introduced.

We moved those sentences to the next paragraphs on methods (Lines 130-136).

L114. Fig S4 would be nice to see in main paper.

Thank you for your comment. We considered adding this figure in the main paper, but decided not to, as we did not see other atmospheric inversion papers describing their inversion framework as a figure in their main manuscript (Please refer to papers in Table S1). However, to incorporate the reviewer’s comment on more description of our methods, we added details in Lines 118-121 and moved the majority of our methods from SI to the main text (Lines 321-421).

L120. What is this online method, why not methods section proper?

We revised the online method and incorporated SI methods into this section (Lines 321-421).

L121-126. I assume these are outputs plotted in figure 1/ If so, reference them. It is weird to see them defined so late and after figure 1 was introduced. Consider reorganizing this.

It was most efficient to include both observations and model simulation results in the same Figure. We refer to the observed CH_4 and $\delta^{13}C$–CH_4 in the introduction, and only refer to the model simulation components of Fig. 1 later in the paper. To help clarify this, we added ‘black line in’ when we refer to Fig. 1 in the introduction (Lines 67 and 96).

L130. Fig S8 was not referenced to in proper sequence (i.e., skipped from S7 to S9).

Thank you for the comment. We revised the figure order accordingly.

L131-139. Figure S9-10 referred to here, but very complicated and not well explained. Why plot latitudinally and why so complicated, without a detailed explanation? Explain the latitudinal trend. Assimilated vs. unassimilated not defined/explained.

Thank you for the comment. We have added more description in the caption of Figs. S9-10 and in the main text (Lines 139-154). We hope this increases the interpretability of the model-data comparison part.

L159. Is this fossil and pyrogenic? Fig 3m shows Asian fossil emissions highest, so interpretation as not totally clear to me.

During 2014-2017, fossil emissions from Asia still showed an increase; however, the total global fossil changes were compensated by decreases in North America and Europe; thus, we described in Line 175 that the contribution from fossil emissions is minor. In contrast, the total size of pyrogenic emissions is ~25 Tg/yr in our simulation, so about ~10 Tg/yr increase in emissions from tropical Asia was a noticeable change that we decided to describe in Lines 181-182.

L165. And N. America? High trend in fig 3n and 3o.

We revised to add the contribution of the increase in microbial emissions from N. America as well (Line 189).

L176. Fig. S15 caption. Define K, E, too.

KIE refers to the kinetic isotope effect, but this figure has now been replaced following the revisions to the OH section (Lines 198-214).

L187. The term correlation is unclear to me. How does the correlation analysis show what you are stating? Either I have confused myself, or you need another sentence in this section to expand your explanation.

We added more details on the explanation of the correlation (Lines 232-235 and Supplementary Information).

L209-211. “fractional contribution of wetland emissions”… I am a bit lost here. Where did this info come from? A half sentence more would help. Reference?

We added a reference and added more description in the sentence (Lines 243-247).

L229. The only region with a sign inversion between methods is Hudson Bay. This was intriguing. Worth adding a sentence to note it and explain why.

We assume the reviewer is referring to the sign difference in the trends of wetland methane emissions between the Joint and CH_4-only inversions in the Hudson Bay region (Fig. 4b). To further investigate this and address comments from Reviewers 2 and 3, we conducted additional analyses using alternative priors with dynamic inundation. In these tests, both the Joint and CH_4-only inversions show negative trends, suggesting that the sign inversion of trends

is sensitive to the choice of prior emissions. Because our manuscript focuses on the broader patterns that remain consistent across different priors, we did not add additional discussion of this pattern.

L257. “A potential changes in” – grammar.

Thank you for your comment. The related text on the OH section is now summarized in Lines 299-301.

L278. Much of this material needs to be in the main text methods section.

Following the reviewer’s comment, we moved the majority of the SI methods into the main text methods section (Lines 321-421).

The presented manuscript, entitled “*Isotopic constraints in methane inversions reveal larger wetland emissions with improved linkage to terrestrial water storage*”, integrates isotopic constraints into an updated global atmospheric methane inversion system – CarbonTracker-CH_4 to better partition global CH_4 source types and to track their temporal dynamics. The authors validated inversions with/without stable carbon isotope ratio ($\delta^{13}C–CH_4$) using a harmonized database of global atmospheric CH_4 and $\delta^{13}C–CH_4$ measurements. They found that the inclusion of isotopic information significantly improved constraints on microbial versus fossil CH_4 sources and uncovered the geographic distribution of increases in CH_4 sources. The study shows that wetland emissions are larger than previously estimated. However, the correlation between regional wetland CH4 emissions and terrestrial water storage (TWS) varies within major wetlands. The manuscript is scientifically sound and clearly presented. Clarifications are needed regarding the impacts of wetland priors and isotopic attribution on inversions. With these adjustments, the study will represent a valuable and influential contribution to Nature Communications and to the broader atmospheric and biogeochemical science community.

Major comments:

1, The $\delta^{13}C–CH_4$ were mostly measured at North America and Europe sites. How would the sparse $\delta^{13}C–CH_4$ measurements in continental South America, Africa, and Australia affect the spatial attribution of microbial and fossil sources in those regions? Lines 187-189 and Fig. S18 tried to address this question partially at the continental scale. The joint scenario simulated continued decreasing fossil emissions and relatively stable pyrogenic emissions in Africa. The simulated fossil emissions in the joint scenarios are higher than in the CH_4-only scenario in South America and Australia. Would you suggest more stable carbon isotope sites in these continents? Also in the supporting materials line 171-172, you “forced the assimilation to closely match remote marine background sites, while some sites were given a very large model-data mismatch because they are likely influenced by strong local sources”. It could be helpful to name the sites (or some example sites) that were not used in assimilation.

2, How will the wetland emission priors affect the spatial pattern of microbial emissions in assimulations? The prior wetland fluxes are derived from a single process model (TEM) with a static wetland distribution. Please discuss potential biases from static wetland area assumptions and whether results change when dynamic wetland datasets are used, especially when linking wetland emissions with TWS. In Southeast US (region 3) and South America (region 5), the emission trends are stronger in the joint scenario than in the CH_4-only scenario, whereas the correlations with TWS are opposite. Please explain other possible environmental factors that could drive the stronger trends in these regions and their implications.

Minor comments:

1, Line 94-95, are there limitations or differences in the estimation of global and regional CH_4 emissions in the referenced studies due to a lack of observational constraints on the stable isotope ratio? Explaining such limitations or differences, if any, can help the audience understand the reason for including $\delta^{13}C–CH_4$ constraints in source partitioning.

2, Line 96-111, this paragraph makes a strong point of utilizing isotopic information in the presented inversion framework with minor impacts of uncertainties in isotopic source signatures.

3, Line 124-125, the three experimental scenarios substantially support the argument of this manuscript. Were the changes in the tropospheric OH scaling factor applied universally to all grids? Please clarify how the scaling factor was applied spatially.

4, Line 137-139, What is the regional shift? Please provide more descriptions of Fig. S10 and explicitly explain the regional shift. Fig. S10 shows CH4-only inversion underestimates $\delta^{13}C$–CH_4, and the underestimation intensifies in the Tropics and Polar Southern Hemisphere after 2015. Do you mean the shift is needed in these two regions?

5, Line 174-176, please provide a few sentences summarizing the changes in the spatial distribution of fossil and microbial sources in the Joint+OH scenario.

6. Line 413-415, Figure 1 seems to show monthly data between 2000 and 2021, not 2022. Did I interpret it wrong? Also in the supporting materials line 196-199, you described eight 5-year inversions with only the first and last year overlapping between periods. However, the interval between the starting years is only four years (including the start and end years). If the interval were 5 years as described, then there would be 4 years of overlap (first two years and last two years). Please correct or clarify it.

7, Supporting materials line 122-123, “larger emissions increases occurring in the tropics”, Asian tropics?

The authors have thoughtfully addressed all of my comments. I appreciate the detailed responses and the revisions made to improve the manuscript. In particular, the inclusion of additional inversion scenarios using alternative wetland priors with dynamic inundation has substantially strengthened the study.

The results provide stronger support for advances in methane source attribution using isotopic constraints, demonstrating that CH_4-only inversions are more sensitive to wetland prior assumptions when estimating total wetland emissions. Additionally, the findings reinforce the manuscript's conclusion that both Joint and CH_4-only inversion scenarios indicate increasing trends in wetland emissions, regardless of whether static or dynamic inundation priors are used.

Overall, I believe the manuscript is significantly improved and suitable for publication in Nature Communications.
